# Transcriptomics Reveals the Mevalonate and Cholesterol Pathways Blocking as Part of the Bacterial Cyclodipeptides Cytotoxic Effects in HeLa Cells of Human Cervix Adenocarcinoma

**DOI:** 10.3389/fonc.2022.790537

**Published:** 2022-03-14

**Authors:** Pedro E. Lázaro-Mixteco, José M. González-Coronel, Laura Hernández-Padilla, Lorena Martínez-Alcantar, Enrique Martínez-Carranza, Jesús Salvador López-Bucio, Ángel A. Guevara-García, Jesús Campos-García

**Affiliations:** ^1^ Laboratorio de Biotecnología Microbiana, Instituto de Investigaciones Químico-Biológicas, Universidad Michoacana de San Nicolás de Hidalgo, Morelia, Mexico; ^2^ Departamento de Biología Molecular de Plantas, Instituto de Biotecnología, Universidad Nacional Autónoma de México, Cuernavaca, Mexico; ^3^ CONACYT-UMSNH, Instituto de Investigaciones Químico-Biológicas, Universidad Michoacana de San Nicolás de Hidalgo, Morelia, Mexico

**Keywords:** cervical cancer, cyclodipeptides, genetic expression, signaling pathways, transcriptome analysis, cholesterol metabolism

## Abstract

The incidence of human cervix adenocarcinoma (CC) caused by papillomavirus genome integration into the host chromosome is the third most common cancer among women. Bacterial cyclodipeptides (CDPs) exert cytotoxic effects in human cervical cancer HeLa cells, primarily by blocking the PI3K/Akt/mTOR pathway, but downstream responses comprising gene expression remain unstudied. Seeking to understand the cytotoxic and anti-proliferative effects of CDPs in HeLa cells, a global RNA-Seq analysis was performed. This strategy permitted the identification of 151 differentially expressed genes (DEGs), which were either up- or down-regulated in response to CDPs exposure. Database analysis, including Gene Ontology (COG), and the Kyoto Encyclopedia of Genes and Genomes (KEGG), revealed differential gene expression on cancer transduction signals, and metabolic pathways, for which, expression profiles were modified by the CDPs exposure. Bioinformatics confirmed the impact of CDPs in the differential expression of genes from signal transduction pathways such as PI3K-Akt, mTOR, FoxO, Wnt, MAPK, P53, TGF-β, Notch, apoptosis, EMT, and CSC. Additionally, the CDPs exposure modified the expression of cancer-related transcription factors involved in the regulation of processes such as epigenetics, DNA splicing, and damage response. Interestingly, transcriptomic analysis revealed the participation of genes of the mevalonate and cholesterol biosynthesis pathways; in agreement with this observation, total cholesterol diminished, confirming the blockage of the cholesterol synthesis by the exposure of HeLa cells to CDPs. Interestingly, the expression of some genes of the mevalonate and cholesterol synthesis such as *HMGS1*, *HMGCR*, *IDI1*, *SQLE*, *MSMO1*, *SREBF1*, and *SOAT1* was up-regulated by CDPs exposure. Accordingly, metabolites of the mevalonate pathway were accumulated in cultures treated with CDPs. This finding further suggests that the metabolism of cholesterol is crucial for the occurrence of CC, and the blockade of the sterol synthesis as an anti-proliferative mechanism of the bacterial CDPs, represents a reasonable chemotherapeutic drug target to explore. Our transcriptomic study supports the anti-neoplastic effects of bacterial CDPs in HeLa cells shown previously, providing new insights into the transduction signals, transcription factors and metabolic pathways, such as mevalonate and cholesterol that are impacted by the CDPs and highlights its potential as anti-neoplastic drugs.

## Introduction

Human cervical cancer (CC) is the third leading cause of cancer death among women around the world, along with breast, lung and colorectal cancers (1) and the second leading cause of death in America. CC arises in normal cervical epithelium as a progressive disease over the course of years, originated by human papillomavirus (HPV) infection. HPV infection results in host genome alterations, leading to the silencing of some tumor-suppressor factors and the induction of aberrant function of tumor-promoting factors (2). These oncogenic factors then drive neoplastic progression. The severity of the outcomes of CC development depends on the specific subtype of the HPV infection. The oncoproteins E5, E6, and E7, encoded by the HPV genome, are the major drivers of oncogenesis in the normal cervical epithelium (3), disrupting the normal function of the histocompatibility complex I (MHC class I), P53, Rb, Notch1, Wnt, MAPK, PI3K/Akt/mTOR, STAT-associated pathways, as well as HSPs such as Hsp90, Hsp70, and Hsp27 (4), which are central players controlling normal cellular growth, differentiation, and immune function (5, 6).

The PI3K/Akt/mTOR signaling pathway is involved in several biological processes such as cell survival, apoptosis, and tumor development and progression (7–9). In this signaling pathway, the mammalian target of rapamycin (mTOR) protein-kinase is a master regulator that acts through two complexes: mTORC1 and mTORC2, playing pivotal roles in the induction of tumor growth (10), where aberrant activation of their components is associated with many cancer types (11–13). mTORC2 is activated by growth factors (14, 15) and is considered important for the maximum activation of Akt by phosphorylation at the serine-473 residue (16), which contributes to tumor pathogenesis (17). Indeed, mTORC1 inhibitors, like rapamycin and other rapalogs, initially showed some promise in treating cancers, but their chronic administration resulted in drug resistance due to feedback activation of the PI3K/Akt pathway by mTORC2 (18, 19). Therefore, simultaneous targeting of downstream mTORC1 and mTORC2 signaling pathways would enhance the efficacy of drugs blocking the upstream tumor-initiating pathways (20–22). Extensive efforts are currently underway to develop potent inhibitors that could simultaneously target both the mTORC1 and mTORC2 signaling pathways (21, 23, 24).

Searching for novel and natural molecules with anticancer activity is always in progress, as natural molecules are considered more target-specific than their synthetic counterparts. Specifically, bacterial cyclodipeptides (CDPs) have been proposed as compounds with strong pharmaceutical potential for cancer treatment (25). CDPs have recently drawn attention for their antiproliferative and cytotoxic effects on cancerous cell lines (24–30). CDPs possess intrinsic physiological advantages compared to other molecules due to higher stability, protease resistance, and conformational rigidity, all these factors increase their ability to specifically interact with biological targets, making them more promising than their linear counterparts (31, 32).

Recently we reported that CDPs isolated from the *Pseudomonas aeruginosa* PAO1 strain composed mostly of cyclo (l-Pro- l-Tyr), cyclo (l-Pro- l-Val), and cyclo (l-Pro- l-Phe), suppress the proliferation of human adenocarcinoma HeLa and CaCo-2 cell lines (29). In HeLa cells, CDPs arrest the cell cycle at the G0–G1 transition by blocking the PI3K/Akt/mTOR pathway, inhibiting the mTORC1/mTORC2 complexes in a TSC1/TSC2-dependent manner. The effects of which lead to inhibition of the phosphorylation of both the Akt-S473 and S6k-T389 protein kinases (24). In addition, the CDPs inhibit protein kinases from multiple signaling pathways involved in survival, proliferation, invasiveness, apoptosis, autophagy, and energy metabolism, such as Ras/Raf/MEK/ERK1/2, PI3K/JNK/PKA, p27Kip1/CDK1/survivin, MAPK, HIF-1, Wnt/β-catenin, HSP27, EMT, CSCs, and most likely receptors, such as EGF/ErbB2/HGF/Met (24). Thus, the antiproliferative effect of the bacterial CDPs may aid to identify the crosstalk of the signaling pathways dysregulated in HeLa cell line.

The proteomic study performed in HeLa cells after CDPs exposure evidenced the blockage of the PI3K/Akt/mTOR, as well as other pathways with a short time of exposure, but it reverted at longer time periods, suggesting inhibition of signal translation of protein kinases. However, the response to longer time periods indicates that subsequent responses related to gene expression could be occurring. The reversion in protein expression/phosphorylation was observed in Vimetin, N-Cadherin, E-Cadherin, VE-Cadherin, MUC1, PCNA, CD31, CD44, CD45, EpCAM, Rb, p27Kip1, Ki67, and HIF-1α (24). In addition, over-expression of the HIF-1α protein was also observed in the CDPs-exposed HeLa cells. This protein is a component of the HIF-1 suppressor, a master regulator of elements involved in glycolysis, and which is dysregulated in tumorigenesis and invasiveness. It is known that the regulation of HIF-1 is closely related to the PI3K/Akt/mTOR pathway, and it has even been shown that Akt and HIF-1 interact synergistically during the development of cancer (33). Additionally, data suggest that the PI3K/Akt/mTOR and HIF-1 pathways crosstalk is implicated in mouse melanoma development and that CDPs targeted these pathways (25).

On the other hand, cholesterol is a lipidic molecule that plays essential roles in fluidity and integrity of membranes and is also involved in regulation of multiple cellular functions such as endocytosis, membrane trafficking, and signaling. Thus, sterol homeostasis in eukaryote cells is essential for embryonic development and tumorigenesis (34). Studies indicate that in cancer cells, the cholesterol synthesis is enhanced, increasing the serum cholesterol levels, which is associated with increased risk for cancer development. Clinical trials using inhibitors (such as statins) of the 3-hydroxy-3-methylglutaryl coenzyme A reductase (HMGCR; the rate-limiting enzyme in the mevalonate and cholesterol synthetic pathway), have showed beneficial and nonbeneficial results. Thus, sterols and downstream products of the mevalonate pathway have a key role in cell proliferation, signaling, protein synthesis, and cell-cycle progression (35). Blocking the mevalonate pathway through inhibition of HMGCR cause apoptosis, by increasing intracellular ROS and P38 activation, and suppressing activation of the Akt and Erk pathways by reducing the metabolic products downstream of the HMGCR reaction, thereby activating diverse small GTP-binding proteins of the Ras and Rho families (36). Anticancer properties of stains (i.e. simvastatin, lovastatin, atorvastatin, provastain) have been explained through pleiotropic effects, impacting on protein prenylation, proliferation and migration, Ras signaling inhibition, and inducing apoptosis by PI3K/Akt/mTOR pathway (35).

Despite the reported effects of bacterial CDPs inhibiting the growth of cancer cells, an in-depth exploration of the mechanisms of action of these drugs is required in order to understand their cytotoxic and antiproliferative effects. The aim of the present study was to perform a RNA-Seq transcriptomic profiling of the gene expression using the HeLa cell line as a human CC model. This study also seeks to identify the up- and downstream elements targeted by the antineoplastic effect of the bacterial CDPs, such as those of the mevalonate and cholesterol pathways.

## Materials and Methods

### Chemicals, Reagents and Cell Culture

Chemicals and reagents included are Dulbecco’s modified Eagle’s medium (DMEM; Sigma-Aldrich), fetal bovine serum (FBS; Gibco Life Technology), and trypsin solution (Sigma Life Science). Cyclodipeptides were obtained from *P. aeruginosa* PAO1 cells-free supernatant as previously described (37). CDPs were dissolved in a DMSO-water ratio of 1:3 to prepare stock solutions (100 mg/mL).

The HeLa human cancer cell line was obtained from the American Type Culture Collection (ATCC, Manassas, VA, USA), which contained mutated the H-Ras oncogene and low-level expression of the P53 tumor suppressor protein. Cells were cultured in complete media [DMEM supplemented with 10% (v/v) FBS, 100 units/mL of penicillin, 40 μg/mL of streptomycin, and 1 μg/mL of amphotericin B (Sigma-Aldrich Co.)]. Cell culture media were changed twice a week and maintained at 37°C under 80% humidity, and incubated in an atmosphere of 5% CO_2_ to confluency. Cells were then trypsin-treated, counted using a hemocytometer chamber, and used for subsequent assays. Cell cultures and other procedures were performed in class II biological safety cabinets. For RNA-Seq and RT-qPCR analysis, HeLa cells were incubated in DMEM complete medium containing the CDPs mixture at 0.01 mg/mL for 15 min and 4 h, along the untreated controls. Afterwards, cells were pelleted by centrifugation for total RNA extraction.

### RNA Extraction, Preparation of cDNA Library and RNA-Seq

Total RNA was isolated using TRIzol reagent (Thermo-Fisher Scientific). Total RNA was treated with DNase (Thermo Fischer Scientific). cDNA libraries were generated using the Illumina TruSeq RNA Sample Preparation Kit according to the manufacturer’s instructions. Transcriptome sequencing was conducted using NextSeq 500 System (Illumina, Inc.). A configuration for pair-end reads with a 75 bp read length was used. RNA-seq Massive sequencing was carried out in the “Unidad Universitaria de Secuenciación Masiva y Bioinformática” (UUSMB), at the Instituto de Biotecnología, UNAM, Cuernavaca, Mor., México. The Sequence Read Archive (SRA) were loaded in the Bioproject ID PRJNA725963 (https://www.ncbi.nlm.nih.gov/sra).

### Bioinformatics Analysis and Heatmaps of RNA-Seq Data

Quality Control (QC) of raw reads was performed using FASTQC software and contamination and adapter removal was carried out using in-house Perl scripts designed by the UUSMB. Because adaptor sequence was present at the three-prime end of some reads, these were further trimmed using CUTADAPT version 0.9.5 with a minimum overlap of two and a minimum length of thirty two (38). Reads were then aligned back to the *Homo sapiens* genome assembly GRCh38.p13 using Bowtie2 version 2.3.4.3 (39). Sam and bed files were generated using SAMtools version 1.9 (40) and BEDTools version 2.27.1 (41). Read counts for each gene were quantified using coverage Bed in BEDTools2 version 2.27.1 (41). DEG analysis was performed using R Bioconductor tool NOISeq (42). Pairwise comparisons among each sample type (Control vs 15 min CDPs-treatment, Control vs 4 h CDPs treatment, and 15 min vs 4 h CDPs-treatments, respectively) were performed. To determine DEGs, a False Discovery Rate (FDR) of adjusted *P* ≤0.05 was used. To generate heatmaps of the top DEGs in RNA-Seq procedure across the samples, the pheatmap v1.0.12 package in R (43), and the Clustergrammer-web Visualization server were utilized (44).

### Gene Ontology and Pathway Enrichment Analyses

Functional Gene Ontology (COG) and Pathway enrichment analysis of the co-modified DEGs were conducted using bioinformatic tools with automated interpretation of genomic data, which perform statistical and network analysis on biological hierarchical vocabularies: the PANTHER classification (45), KEGG resource (46), Pathway Commons web-server (47), WikiPathways database (48), and ShinyGO v0.61 tool (49). COG functional categories falling under biological processes, with a q-value ≤ 0.05 following hypergeometric testing were considered significantly over-represented. In the hierarchy of COG, a gene can be represented in more than one category because of the functional versatility of genes, but only once within each category.

### Protein–Protein Interaction Network (PPI) and Transcription Factor Prediction

The STRING (version 11.0, http://www.string-db.org/) database web-server application was used to predict whether gene-encoded proteins interacted with each other. A PPI network was constructed for the DEGs identified in the current study. The minimum required interaction score parameters were set at the medium confidence level (50). In order to perform TF enrichment analysis, the ChEA3 database web-server application was implemented on the co-modified DEGs to obtain differential TFs and a transcription regulatory network (TF-target network) was constructed (51). ChEA3 covers 1632 site-specific TFs and offers a selection of six primary reference gene set libraries, generated from various sources of distinct data: 1) GTEx and ARCHS4 libraries containing TF-gene co-expression RNA-seq data, 2) ENCODE, Literature ChIP-seq and ReMap containing TF-target associations from ChIP-seq experiments, and 3) The Mean Rank integration method was selected and individual enrichment outcomes for each library are consequently integrated; thus, producing an improved composite rank of potentially implicated prioritized TFs (51).

### RT-qPCR and RT-PCR Analysis

Total RNA treated with DNase (Thermo Fischer Scientific) was utilized to obtain the first cDNA strand by using Superscript II Reverse Transcriptase (Thermo Fischer Scientific) and oligo-dT primer in the reaction volume of 30 μl for 3 μg of RNA material, according to the manufacturer’s instructions. RT-qPCR was performed on a LightCycler Nano (Roche, Basel, Switzerland) and the amplification was carried out using 75 ng cDNA for each reaction based on the Power SYBR Green PCR Master Mix (Thermo Fisher Scientific) as fluorescent probe. After an initial denaturation step of 10 min at 95°C, the product was routinely examined using a dissociation curve, and the amount of transcript was compared with the relative Ct method with glyceraldehyde 3-phosphate dehydrogenase (GAPDH) as internal reference control. The 2^−ΔΔ Cq^ method was utilized for analysis of the experimental data of the genes *ATRX*, *BCL6*, *EGR3*, *COL6A1*, *ANKDR12*, and *DGR8*.

For the mRNA expression of the *HMGS1*, *HMGCR*, *SREBF1*, *IDI1*, *MSMO1*, *SQLE*, *ACAT1*, and *RHOA* genes, semi-quantitative RT-PCR was carried out using 50 ng cDNA obtained with ImProm II-Reverse Transcriptase reagent kit (Promega, Q4100) and amplified using PCR Platinum Super Mix High Fidelity (Invitrogen). PCR was performed using the Bio Rad T100TM Thermal Cycler at 94°C for 3 min, followed by cycles of 15 sec at 94°C, 15 sec at 54°, 60° or 64°C and 15 sec at 72°C; samples were taken at different cycles for DNA quantitation. The products were examined using amplification curves, the amounts of transcript were obtained at 20 cycles of the exponential amplification curve and expressed as relative units using the Image J software. Oligonucleotides sequences utilized are shown in **Supplementary Table S1**.

### Determination of Cholesterol and Metabolites of Mevalonate Pathway

For cholesterol and mevalonate metabolites analysis, cells-free supernatants of HeLa cultures grown as described above were used. 10 mL of cell-free supernatants and cells-pellets were lyophilized and separately extracted with 1 mL methanol. After filtering the samples were dried and dissolved in 200 µL ethanol/methanol (1:1). Cholesterol was determined by spectrophotometry analyzer at 505 nm as described by the provider using the Cholesterol SL-234-60 determination Kit (Sekisui Diagnostics). Metabolites of mevalonate pathway were determined on cell-free supernatant samples by quantitation of organic acids accumulated, such as 3-hydroxyhex-4-enoic acid by GC-MS as described by Pitt et al. (52), with some modifications (52). Briefly, samples extracted with methanol were injected in GC-MS (GC; Agilent 6850 Series II equipped with MS-5973); using a Zebron ZB-WAXplus column, 30 m length, I.D. 0.25 mm, Film 0.25 mm (Phenomenex), sample injection was performed at a splitless mode at temperature of 280°C. The oven temperature was programmed to start at 70°C, maintained in isothermal for 3 min, then increased to 250°C at a rate of 10°C/min, and then an isothermal for 8 min. Compounds were identified by SIM method using the ion fragmentation profiles described for organic acids such as trans-3-hydroxyhex-4-enoic (M+ 274: m/z 73, 143, 147, 157, 259); 3,5-dihydroxyhexanoic 1,5 lactone (M+ 202: m/z 73, 101, 145, 187); trans-5-hydroxyhex-2-enoic (M+ 274: m/z 73, 117, 147, 230, 259); 4-hydroxy-6-methyl-2-pyrone (M+ 198: m/z 73, 170, 183, 198); 5-hydroxy-3-ketohexanoic (M+ 319: m/z 73, 117, 147, 275, 304) (52). Quantitation was performed using the relative values of the peak areas in chromatograms by using GC-MS equipment.

### Statistical Analysis

False discovery rate (FDR) filtering and *P* values *≥ 0.95* were used to identify the mRNAs expression that were significantly different between untreated and CDPs-exposed HeLa cells. These differentially expressed mRNAs were identified through fold change filtering. For correlation analysis, data obtained from RNAseq were analyzed by correlation analysis of response variables (treatments) *vs* data of expression intensity for each DEG (cases) using the STATISTICA software (Data Analysis Software System 8.0; Stat Soft Inc). Other data were statistically analyzed using GraphPad Prism 6.0 software (GraphPad Software).

## Results

### RNA-Sequencing Data

Gene expression profiles from HeLa cells were determined at a dose of 0.1 mg/mL of CDPs for 15 min and 4 h exposure times, conditions previously established to induce apoptosis and differential protein expression profiles (24, 29). After removing reads with adapters, unknown nucleotide sequences, and low-quality reads of sequencing, a total of 10.3 GB of clean data was acquired. As shown in **Table 1A**, the Q20 and Q30 percentages (accuracy rate of base identification > 99.9% and error rate of sequencing < 1%) were more than 95.3% and 92.9%, respectively. A total of 43.3 million of 76 nt clean reads were generated using sequences from randomly primed cDNA libraries, prepared from polyA+ fractions of HeLa RNA. Of these sequences, 40.3 million reads representing more than 93% of the reference human genome, annotated as exons using Bowtie2 (39), were aligned. A total of 37.8 million reads, representing more than 87%, were uniquely mapped. The multiple mapped alignments were only 1,328,907 reads, representing less than 4.5% (**Table 1B**). The different expression levels were analyzed, normalizing the mapped reads in the samples. FPKM (Fragments Per Kilobase of transcript) was an indicator of gene expression; a fold change >1.49 and FDR < 0.01 were taken as the screening criteria in the process of gene detection/selection. Analysis of those < 43.3 million randomly derived sequence reads aligned to the human genome, revealed that this approach is reliable for studying gene expression changes as a result of the antiproliferative effects of bacterial CDPs in the HeLa cell line of human cervical cancer, in a global transcriptome-wide fashion.

**Table 1A:** Summary of sequencing data.

Sample	Clean reads	Clean bases (bp)	GC content	Q20 bases	Q30 bases	% ≥ Q20	% ≥ Q30
Control	7,861,906	597,504,856	51.01%	569,419,437	554,944,825	95.29%	92.87%
CDPs-15 min	11,983,679	910,758,920	52.43%	868,918,019	845,476,240	95.41%	92.83%
CDPs-4 h	23,493,492	1,785,505,392	50.89%	1,702,792,904	1,663,564,682	95.36%	93.17%

Clean reads were paired-end reads of clean data.

**Table 1B:** Summary of comparative analysis.

Sample	Control	CDPs-15 min	CDPs-4 h	Total
Total reads	7,861,906	11,983,679	23,493,492	43,339,077 (93%)
Mapped reads	7,175,942 (91.27%)	10,929,757 (91.21%)	21,565,316 (91.79%)	40,356979 (91%)
Unique mapped reads	6,872,404 (87.40%)	10,408,923 (86.86%)	20,583,259 (87.61%)	37,864,585 (87%)
Multiple map reads	382,876 (4.87%)	533,079 (4.45%)	945,498 (4.02%)	1,328,907 (4.5%)

### Identification of Global DEGs in HeLa Cells Exposed to CDPs

**Figure 1:**
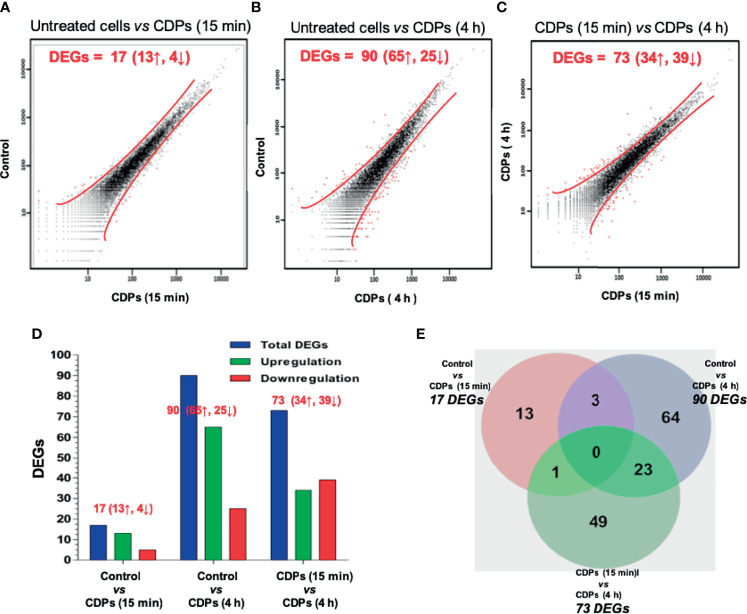
RNA-Seq profiling of HeLa cells exposed to CDPs. **(A-C)** Scatter plots showing the correlation of gene abundance. Red lines delimit points that represent genes up-regulated and down-regulated by at least 1.5-fold at *P* ≥ 0.95, while black dots inside red lines indicate transcripts that did not change significantly. Red words indicate the number of DEGs; ↑ up-regulated, ↓ down-regulated. **(D)** A summary of total, up- and down-regulated genes between treatments is shown. **(E)** Venn diagram shows the DEGs in each treatment and the overlapping in two or three CDPs treatments.

The mapped 22,201 genes were statistically filtered using adjusted *P* values ≤ 0.05 for all samples and selected with a minimum detection threshold of 10 counts for each gene in all samples. The number of genes below these thresholds of detection was 10,127. A scatter plot illustrating different expression is shown (**Figures 1A–C**).

Following filtration, a change of ≥1.50 fold was observed in a total of 13 up-regulated and 4 down-regulated genes in cells treated with CDPs, compared to untreated cells (**Figures 1A, D**); while at 4 h of CDPs exposure, a total of 65 up-regulated and 25 down-regulated genes were observed (**Figures 1B, D**). Finally, comparing DEGs between 15 min and 4 h CDP exposure, 34 up- and 39 down-regulated genes were identified (**Figures 1C, D**).

Seeking to identify both unique and common genes differentially expressed and associated with the effect of CDPs exposure, a Venn diagram was constructed (**Figure 1E**; full gene list is showed in **Supplementary Table S2**). None of the DEGs were shared among all the subgroups compared. Three DEGs were shared among the comparison of control *vs* 15 min and control *vs* 4 h CDPs exposure; only one DEG was shared among the comparison of control *vs* 15 min, and 15 min *vs* 4 h; and 23 DEGs were shared among the comparison of 15 min *vs* 4 h, and control *vs* 4 h, which suggests that more complex biological events occurred after 4 h of CDPs exposure. In addition, clustering showed two well-defined DEG groups: one cluster associated with up-regulated genes with a short time of CDPs exposure (15 min) and a second group strongly associated with up-regulated genes at a longer time of CDPs exposure (4 h). Also, a smaller number of DEGs with a down-expression profile was observed (**Figure 1D**). Thus, a total of ~151 DEGs in the global comparison changed in expression level, representing the genes which were used in subsequent analyses (**Supplementary Table S3** and **Supplementary Figure S1**).

### Functional Enrichment Analysis of DEGs in HeLa Cells Exposed to CDPs

Analysis of gene expression patterns using RNA-Seq revealed several biological processes and biological pathways that could be targeted when HeLa cells are exposed to bacterial CDPs. Gene Ontology analysis of the 151 DEGs using bioinformatics platforms, including the PANTHER classification System (http://www.pantherdb.org), ShinyGO v0.61 GO terms (http://bioinformatics.sdstate.edu/go/), Reactome FIVIz (https://reactome.org/tools/reactome-fiviz), STRING-db v.10 (https://string-db.org), Pathway Commons tool (https://www.pathwaycommons.org), and the KEGG pathway mapping (https://www.genome.jp/kegg/mapper.html), clustered the DEGs into two major categories: biological processes and biological pathways.

**Figure 2:**
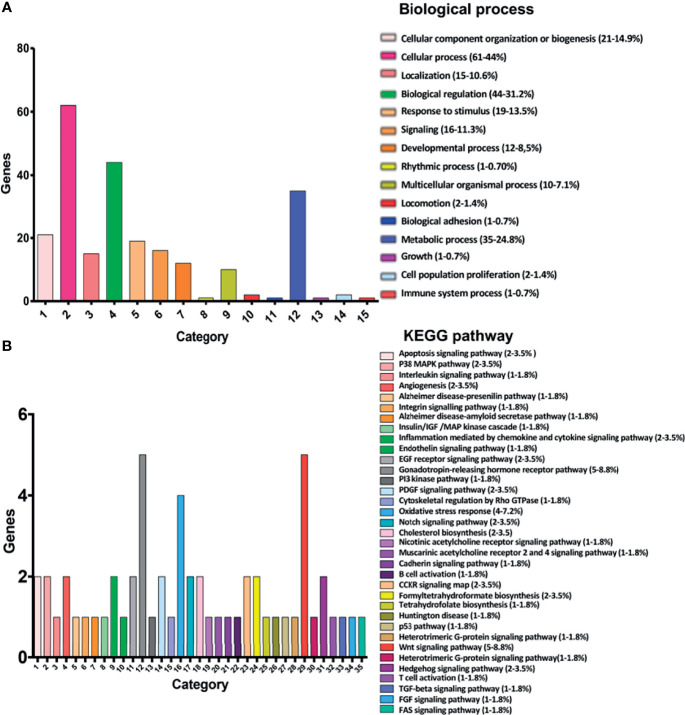
Classification of the 151 main DEGs according to the Gene Ontology (COG) principles. **(A)** Classification of biological processes shows the 15 most significantly enriched COG terms for DEGs. **(B)** Classification of canonical pathways showing the 35 most significantly enriched COG terms for DEGs. The *X*-axis is the COG category classification; the left of the *Y*-axis is the number of genes. Chart tooltips are read as: category name (gene numbers, percentage).

The present study identified 15 biological processes (**Figure 2A**; *P* ≤ 0.05), including cellular component organization or biogenesis (21-14.9%), cellular processes (61-44%), localization (15-10.6%), biological regulation (44-31.2%), response to stimulus (19-13.5%), signaling (16-11.3%), developmental processes (12-8,5%), rhythmic processes (1-0.70%), multicellular organismal processes (10-7.1%), locomotion (2-1.4%), biological adhesion (1-0.7%), metabolic processes (35-24.8%), growth (1-0.7%), cell population proliferation (2-1.4%), and immune system processes (1-0.7%), although there exist others. The biological pathways (**Figure 2B**; *P* ≤ 0.05) included insulin/IGF/MAP kinase cascade (1-1.8%), P38/MAPK (2-3.5%), interleukins (1-1.8%), EGF receptor (2-3.5%), PI3K kinase(1-1.8%), PDGF (2-3.5%), Notch (2-3.5%), Cadherin (1-1.8%), P53 (1-1.8%), Gonadotropin-releasing hormone receptor pathway (5-8.8%), Hedgehog (2-3.5%), TGF-beta (1-1.8%), FGF (1-1.8%), FAS signaling pathways (1-1.8%), Gonadotropin-releasing hormone receptor pathway (5-8.8%), Angiogenesis (2-3.5%), Cholesterol biosynthesis (2-3.5%), as well as other pathways that are involved in inflammation (2-3.5%), oxidative stress response (4-7.2%), apoptosis (2-3.6%), cytoskeleton (1-1.8%), and T and B cell growth/activation (2-3.6%).

### Networks and KEGG Pathways Analysis of DEGs Associated to Cancers in HeLa Cells Exposed to CDPs

**Figure 3:**
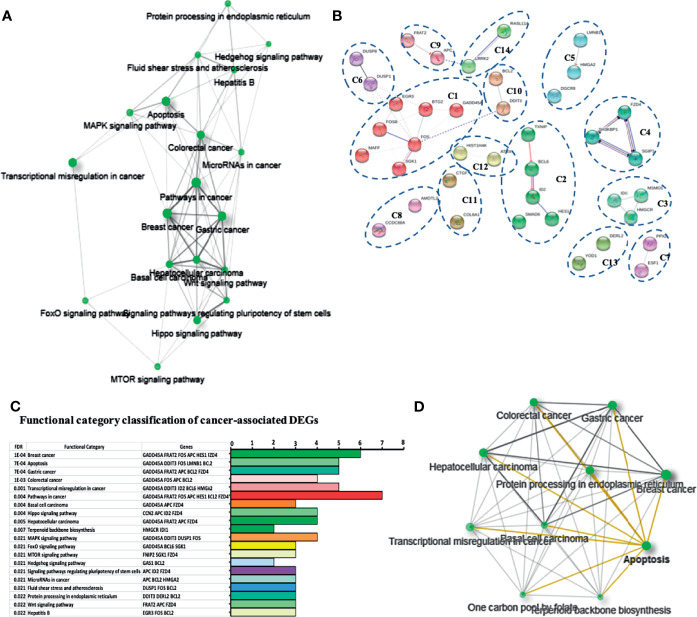
Network and functional enrichment analysis of cancer related DEGs. **(A)** KEGG network shows the relationship between enriched pathways. Darker nodes are more significantly enriched gene sets. Bigger nodes represent larger gene sets. Thicker edges represent more overlapped genes. **(B)** STRING network shows fourteen cancer related DEGs. Each cluster represents a set of highly connected nodes and is illustrated in a discrete color. **(C)** Functional category classification shows the most representative DEGs and pathways related to cancer from **(A, B)**. **(D)** Network of the relationship between enriched cancer types.

The annotation results of the gene KEGG identified a total of 35 distinct functional clusters in the global DEG analysis and nineteen cancer-related functional clusters, each one consisting of a set of highly connected nodes (**Figure 3A**). Except for pathways in apoptosis and transcriptional dysregulation in cancer, the top 8 signaling pathways annotated with a significant number of DEGs were: mTOR, Hippo, FoxO, CSC, Wnt, MAPK, apoptosis, and Hedgehog signaling pathways (**Figure 3A**). To seek potential interactions between DEGs according to CDPs exposure, the ShinyGO v0.61 and STRING tools were employed. Active interaction sources, including text mining, experiments, databases, and co-expression, as well as species limited to *Homo sapiens* and an interaction score > 0.4, were applied to construct the networks. The STRING network shows the 14 networks of the cancer related DEGs associated with CDPs exposure on HeLa cells (**Figure 3B**).

In the STRING networks, the main clusters corresponded to the network C1 which is associates to the DEGs, *FOS*, *FOSB*, *BTG2*, *EGR3*, *GADD45A*, *SGK1*, and *MAFF* and interacts with the C6, C10, C11, and C12 clusters, containing DEGs such as *DUSP1*, *DDIT3*, *BCL2*, *CTGF*, and *ATRX*. A classification and frequency analysis of each DEG associated with cancer networks in some of the more frequent cancer types such as breast, gastric, and colorectal cancers, are shown (**Figures 3C, D**).

**Figure 4:**
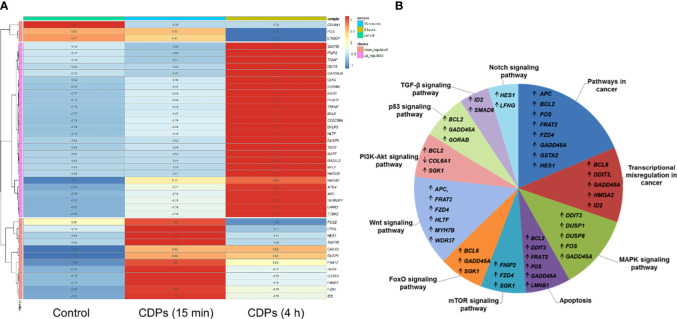
Identification of cancer-related genes and pathways. **(A)** Heat maps of the 53 cancer-related DEGs common in the three comparative transcriptomes. **(B)** Distribution of cancer-related transcripts in signaling pathways. Up arrows and down arrows indicate the overlap of up-regulated and down-regulated genes, respectively. Total DEGs were analyzed by Gene Ontology (COG) annotation and the Kyoto Encyclopedia of Genes and Genomes (KEGG). All genes were pooled to build the differential pathways, which helped to reveal the signaling pathways and key regulatory genes in DEGs.

To deepen in the identification of pathways and DEGs related to cancer, a heat map showed 41 main DEGs in HeLa cells exposed to CDPs (**Figure 4A**), as well as the different cancer-associated pathways (**Figure 4B**, **Table 2**). In this context, in HeLa cell line the following signaling pathways are impacted by bacterial CDPs: PI3K-Akt, mTOR, FoxO, Wnt, MAPK, P53, TGF-β, Notch, and apoptosis FasL/Bcl2-dependent (**Supplementary Figure S2**). In addition, outside those mentioned, a group of transcription factors, which dysregulation has been widely associated with cancer phenotype was identified, including DEGs such as *BCL6*, *DDIT3*, *GADD45A*, *HMGA2*, and *ID2* (**Figure 4B**).

**Table 2:** Identification of cancer-related genes and pathways in CDPs-treated HeLa cells.

Pathway	DEGs CDPs-modified
Pathways associated to cancer	*APC*; circRNA APC regulator of WNT signaling pathway
	*BCL2*; BCL2 apoptosis regulator
	*FOS*; Fos proto-oncogene, AP-1
	*FRAT2*; FRAT regulator of WNT signaling pathway
	*FZD4*; frizzled class receptor
	*GADD45A*; growth arrest and DNA damage inducible alpha
	*GSTA2*; glutathione S-transferase alpha
	*HES1*; Hes family bHLH transcription factor
Apoptosis	*BCL2*; BCL2 apoptosis regulator
	*DDIT3*; DNA damage inducible transcript
	*FOS*; Fos proto-oncogene, AP-1 transcription factor subunit
	*GADD45A*; growth arrest and DNA damage inducible alpha
	*LMNB*1; lamin B1
Transcriptional misregulation in cancer	*BCL6*; BCL6 transcription repressor
	*DDIT3*; DNA damage inducible transcript
	*GADD45A*; growth arrest and DNA damage inducible alpha
	*HMGA2*; high mobility group AT-hook
	*ID2*; inhibitor of DNA binding
MAPK signaling pathway	*DDIT3*; DNA damage inducible transcript 3
	*DUSP1*; dual specificity phosphatase
	*DUSP8*; dual specificity phosphatase
	*FOS*; Fos proto-oncogene, AP-1 transcription factor subunit; growth arrest and DNA damage inducible alpha
mTOR signaling pathway	*FNIP2*; folliculin interacting protein
	*FZD4*; frizzled class receptor
	*SGK1*; serum/glucocorticoid regulated kinase
FoxO signaling pathway	*BCL6*; BCL6 transcription repressor
	*GADD45A*; growth arrest and DNA damage inducible alpha
	*SGK1*; serum/glucocorticoid regulated kinase
Wnt signaling pathway	*APC*; circRNA APC regulator of WNT signaling pathway
	*FRAT2*; FRAT regulator of WNT signaling pathway
	*FZD4*; frizzled class receptor
	*HLTF*; Helicase-like transcription factor
	*MYH7*B, myosin/7B
	*WDR37*, WD repeat-containing protein
PI3K-Akt signaling pathway	*BCL2*; BCL2 apoptosis regulator
	*COL6A1*; collagen type VI alpha 1 chain
	*SGK1*; serum/glucocorticoid regulated kinase
p53 signaling pathway	*BCL2*; BCL2 apoptosis regulator
	*GADD45A*; growth arrest and DNA damage inducible alpha
	*GORAB*; golgin, RAB6 interacting
TGF-beta signaling pathway	*ID2*; inhibitor of DNA binding
	*SMAD6*; SMAD family member
Notch signaling pathway	*HES1*; Hes family bHLH transcription factor
	*LFNG*;LFNG O-fucosylpeptide 3-beta-N-acetyl-glucosaminyl-transferase

According to the KEGG database, one gene may be involved in several pathways or interact with several other genes. All DEGs were pooled to build the differential pathways, which helped us to reveal the signaling pathways and key regulatory genes in differentially expressed genes (DEGs).

### Networks and KEGG Pathways Analysis of Differentially Expressed Transcription Factors (DETFs) in HeLa Cells Exposed to CDPs

To further evidence the connection of transcriptome changes, biological processes and pathways, the list of the global co-modified DEGs from the two CDPs treatments were subjected to analysis of the expression of transcription factors (TFs) and functional enrichment analyses, using an established computational ChEA3 TF tool that offers a better understanding of gene regulatory networks. **Supplementary Table S4** provides a complete list of all 1632 site-specific TFs covered by ChEA3 TF, which prioritization was based on their integrated Mean Rank score, along the overlapping genes found affected by CDPs exposure for each TF entry.

**Figure 5:**
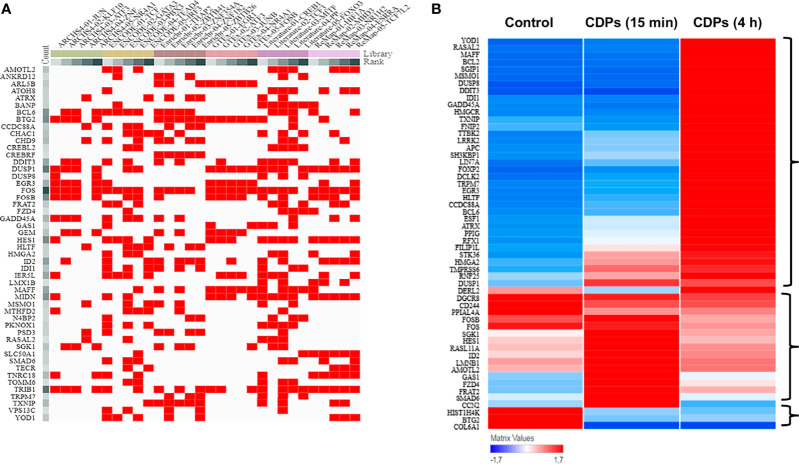
ChEA3 analysis of transcription factors. **(A)** The interactive cluster-gram shows the overlapping of the top 50 query targeted genes from this study, among the top 30 gene library results. **(B)** Heat map of the top DEGs with modified expression in the transcriptome of HeLa exposed to CDPs at t= 0 (Control), 15 min and 4 h.

TF analysis revealed that from the 1632 TFs identified, 151 DEGs have TF/target–gene associations and the more enriched genes are related with a neoplastic phenotype (**Supplementary Figures S1**, **S2**). The top 55 prioritized DEGs (of which most are DETFs) according to their ranking score are shown (**Figure 5A**, **Table 3**). The heatmap network revealed the main DETFs in CDPs-exposed HeLa cells, showing high frequency, and significant differences, in expression levels (**Figure 5B**). Documented information about major biological functions in the context of cancer and the acquired ranks for each individual library are also shown (**Table 2**). Crucial cancer-related processes are observed including the cell cycle, control of apoptosis, DNA-damage response, proliferation, transcriptional dysregulation of oncogenes, and tumor suppressors TFs-regulated. In addition to widely studied cancer-related DETFs, some also corresponded to non-coding RNA transcripts (**Table 3**, **Supplementary Table S4**).

**Table 3:** Classifications of the 53 DETFs according to the ChEA3 TF analysis.

GO Biological Process				
N genes	High level GO category			Genes
22	Regulation of molecular function			*TBC1D2 ID2 GADD45A TRIB1 BCL2 FZD4 DDIT3 FNIP2 RASAL2 LFNG HMGCR HES1 CCN2 GEM MIDN DUSP8 MTRNR2L10 TXNIP SMCR8 DHFR SGK1 FOS*
21	Regulation of response to stimulus			*LFNG GADD45A BCL2 TRIB1 DDIT3 CCN2 SMAD6 HMGCR HES1 CREBRF FZD4 YOD1 DUSP8 RTN4RL1 MTRNR2L10 CHAC1 SMCR8 GAS1 DHFR FOS BANP*
20	Response to stress			*BCL2 DDIT3 YOD1 GADD45A CCN2 FOS FNIP2 HMGCR ID2 SGK1 CHAC1 CREBRF FZD4 RTN4RL1 TXNIP SMAD6 ASCL2 TRIB1 DHFR MAFF*
19	Regulation of signaling			*LFNG GADD45A BCL2 TRIB1 DDIT3 CCN2 SMAD6 HMGCR HES1 CREBRF MIDN SYNPO FZD4 DUSP8 MTRNR2L10 CHAC1 SMCR8 GAS1 BANP*
18	Regulation of multicellular organismal process			*HES1 ID2 RFLNB ASCL2 MAFF ATOH8 DDIT3 LFNG HMGCR CCN2 SMAD6 FOS BCL2 TRIB1 FZD4 RTN4RL1 SGK1 LINGO1*
17	Regulation of developmental process			*HES1 ID2 RFLNB ASCL2 MAFF CCN2 ATOH8 DDIT3 LFNG HMGCR FOS BCL2 TRIB1 FZD4 RTN4RL1 SMAD6 LINGO1*
14	Regulation of biological quality			*SAMD4A BCL2 DDIT3 HMGCR SEC24A ID2 CCN2 PKNOX1 MIDN SYNPO FZD4 SGIP1 SGK1 MAFF*
13	Anatomical structure morphogenesis			*AMOTL2 HES1 SGK1 FZD4 RFLNB LFNG ID2 CCN2 SMAD6 PKNOX1 ATOH8 BCL2 LINGO1*
13	Regulation of localization			*AMOTL2 BCL2 HMGCR SEC24A SGIP1 GEM MIDN DDIT3 YOD1 ATOH8 TRIB1 GAS1 SGK1*
12	Cell proliferation			*BCL2 HMGCR HES1 ID2 CCN2 ATOH8 ASCL2 TXNIP SMAD6 TRIB1 FRAT2 SGK1*
12	Response to external stimulus			*SMAD6 BCL2 HMGCR ID2 FOSB FOS TRIB1 DDIT3 RTN4RL1 TXNIP GADD45A SGIP1*
12	Macromolecule localization			*TBC1D2 ARL5B HMGCR SEC24A SGIP1 MIDN BCL2 BANP FZD4 DDIT3 YOD1 TXNIP*
11	Cellular component biogenesis			*SGIP1 SYNPO FNIP2 HMGCR SEC24A CCN2 SMAD6 DDIT3 HES1 BCL2 SMCR8*
11	Cellular localization			*TBC1D2 ARL5B SEC24A HMGCR BCL2 BANP DDIT3 YOD1 TXNIP GEM GAS1*
**GO Molecular Function**				
**Enrichment FDR**	**Genes in list**	**Total genes**	**Functional Category**	**Genes**
0.022039758	6	250	Microtubule binding	*CCDC88A KIF20B CENPE APC TTBK2 LRRK2*
0.022039758	3	33	Receptor antagonist activity	*MTRNR2L10 MTRNR2L1 MTRNR2L6*
0.023281805	3	43	Receptor inhibitor activity	*MTRNR2L10 MTRNR2L1 MTRNR2L6*
0.036692194	9	816	RNA polymerase II regulatory region sequence-specific DNA binding	*BCL6 FOSB FOS DDIT3 EGR3 MAFF HLTF FOXP2 RORB*
0.036692194	7	542	RNA polymerase II proximal promoter sequence-specific DNA binding	*FOSB FOS DDIT3 MAFF HLTF FOXP2 RORB*
0.036692194	15	1673	DNA-binding transcription factor activity, RNA polymerase II-specific	*BCL6 EGR3 MAFF DDIT3 HLTF FOSB FOXP2 BTG2 FOS RORB ZFY ATRX CREBL2 ZNF236 ZNF534*
0.036692194	7	556	Proximal promoter sequence-specific DNA binding	*FOSB FOS DDIT3 MAFF HLTF FOXP2 RORB*
0.036692194	9	823	RNA polymerase II regulatory region DNA binding	*BCL6 FOSB FOS DDIT3 EGR3 MAFF HLTF FOXP2 RORB*
0.036692194	10	1029	Double-stranded DNA binding	*CDC6 BCL6 FOSB FOS DDIT3 EGR3 MAFF HLTF FOXP2 RORB*
0.036692194	15	1793	DNA-binding transcription factor activity	*BCL6 ZNF236 EGR3 MAFF DDIT3 FOXP2 FOS HLTF CREBL2 FOSB BTG2 RORB ZFY ATRX ZNF534*
0.036692194	4	171	Helicase activity	*HLTF ATRX HFM1 CHD9*
0.036692194	15	1929	Drug binding	*PPIG PPIAL4A DHFR HLTF ATRX TRPM7 CDC6 CLK4 TTBK2 KIF20B CENPE HFM1 DCLK2 CHD9 LRRK2*
0.036692194	6	339	Tubulin binding	*CCDC88A KIF20B CENPE APC LRRK2 TTBK2*
0.036692194	2	21	Cyclosporin A binding	*PPIG PPIAL4A*
0.036692194	11	1189	Sequence-specific DNA binding	*CDC6 BCL6 FOSB FOS DDIT3 EGR3 MAFF FOXP2 BCL2 HLTF RORB*
0.036692194	2	17	Microtubule plus-end binding	*APC TTBK2*
0.036692194	10	920	Sequence-specific double-stranded DNA binding	*CDC6 BCL6 FOSB FOS DDIT3 EGR3 MAFF HLTF FOXP2 RORB*
0.037282737	9	875	Transcription regulatory region sequence-specific DNA binding	*BCL6 FOSB FOS DDIT3 EGR3 MAFF HLTF FOXP2 RORB*
0.040840296	6	441	DNA-binding transcription activator activity, RNA polymerase II-specific	*MAFF DDIT3 HLTF FOSB FOS RORB*
0.049765976	5	330	Ubiquitin protein ligase binding	*HLTF APC SLF1 BCL2 YOD1*

Statistical correspondence analysis of the 151 principal DEGs in the HeLa cells exposed to CDPs (using the numerical values of mRNA reads obtained from the RNA-Seq), clearly grouped the DEGs into three correlation groups: i) a group of 31 DEGs with modified expression in correlation with the CDPs exposure in a short time period (15 min); ii) a second group of 95 DEGs associated with the CDPs exposure at the longer exposure time period (4 h); and finally, iii) a third group of DEGs that more closely resembled the control (HeLa cells without treatment) (**Supplementary Figure S2**). The group of 95 DEGs associated with CDPs exposure was analyzed in detail by the same correspondence analysis (green circle in **Supplementary Figure S2**), whose cross information with the ranking score on database and heatmap network showing high frequency, and significant differences in expression levels (**Figures 5A, B**, **Table 3**), revealed the main DEGs correlating with the effect of CDPs-exposed HeLa cells. These DEGs were i.e., *YOD1*, *RASAL2*, *MSMO1*, *MAFF*, *BCL2*, *DUSP8*, *DDIT3*, *IDI1*, *GADD45A*, *HMGCR*, *LRRR2*, *EGR3*, *BCL6*, *ATRX*, *FOSB*, *FOS*, *SGK1*, *GAS1*, *FZD4*, *FRAT2*, *SMAD6*, *BTG2*, and *COL6A1* (for more details of DEGs implication and function, see **Supplementary Table S5**). Therefore, these DEGs could be considered as the top CDPs-associated targets on HeLa cells.

**Figure 6:**
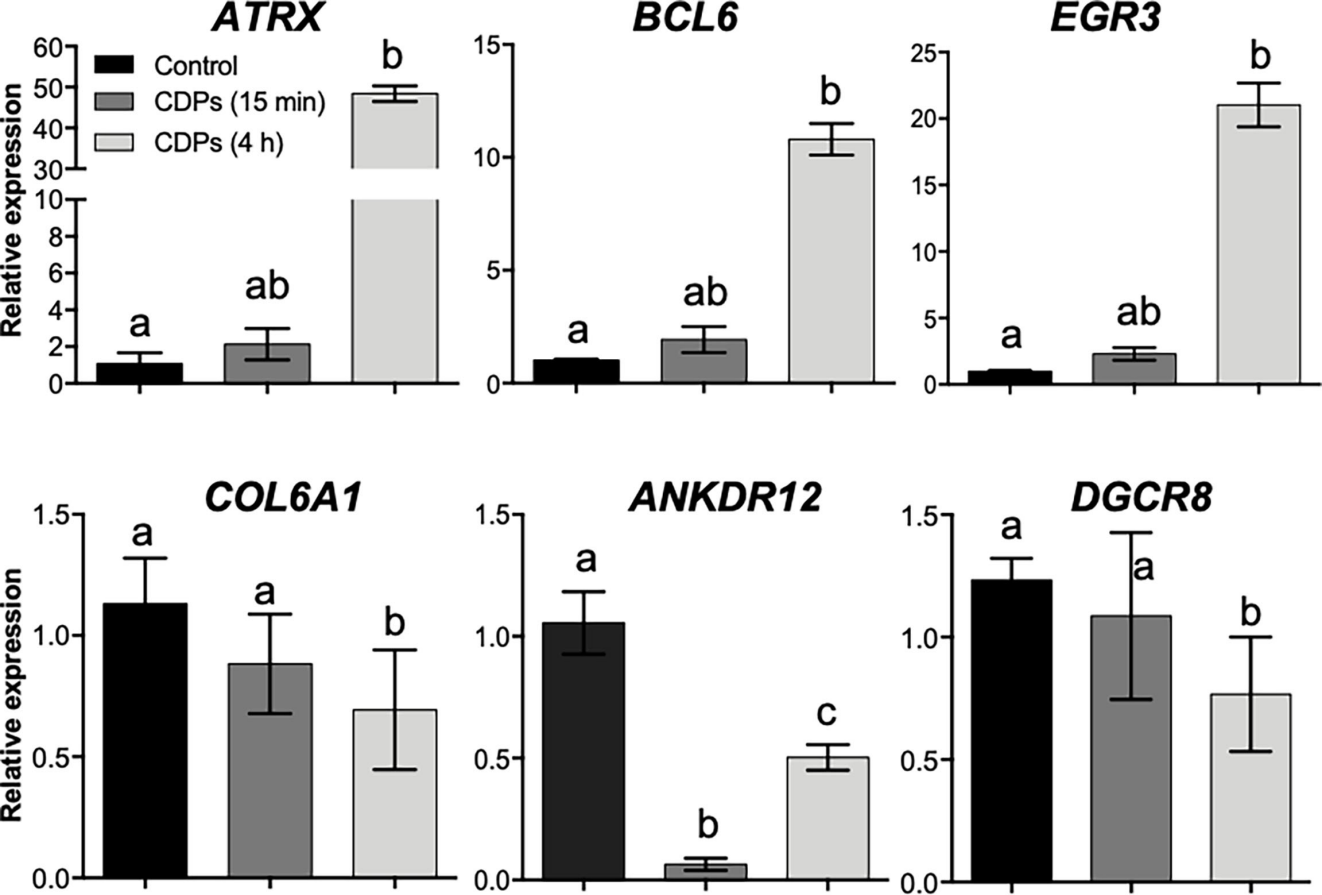
Validation of RNA-Seq data by qRT-PCR. The relative expression levels of cancer-related transcripts through RT-qPCR assays for HeLa cells exposed to CDPs at 15 min and 4 h are shown. Data were analyzed by the 2–ΔΔ^Ct^ method using GAPDH as a reference gene. The results are presented as expression-fold changes. Each column represents the means ± SEM from three biological samples by triplicate each. Bars represent means ± SE of three independent assays. One-way analysis of variance (ANOVA) was carried out, with a Bonferroni *post-hoc* test; statistical significance (*P ≤* 0.05) of differences between treatments is indicated with lowercase letters.

To verify the mRNA expression in HeLa cells in the RNA-Seq approach, we randomly selected six target genes (*ATRX*, *BCL6*, *EGR3*, *FOS*, *COL6A1*, and *ANKDR12*), which expression was modified in the top DEG-enriched ranking score, to verify their relative mRNA expression in HeLa cells through RT-qPCR (**Figure 6**). The *ATRX*, *BCL6*, and *EGR3* genes belonging to TFs showed moderate increases in mRNA level at 15 min of CDPs exposure, but the mRNA levels clearly increased at 4 h of CDPs exposure, in agreement with the RNA-Seq profile (**Figure 6**). The *COL6A1*, *ANKDR12*, and *DGCR8* transcripts showed a less clear expression behavior, but it correlated with the RNA-Seq profile, that showed decreased levels of expression in the CDPs-exposed samples. Thus, the RT-qPCR expression analysis supported and validated the RNA-Seq.

### CDPs Impacted the Mevalonate and Cholesterol Pathways

**Figure 7:**
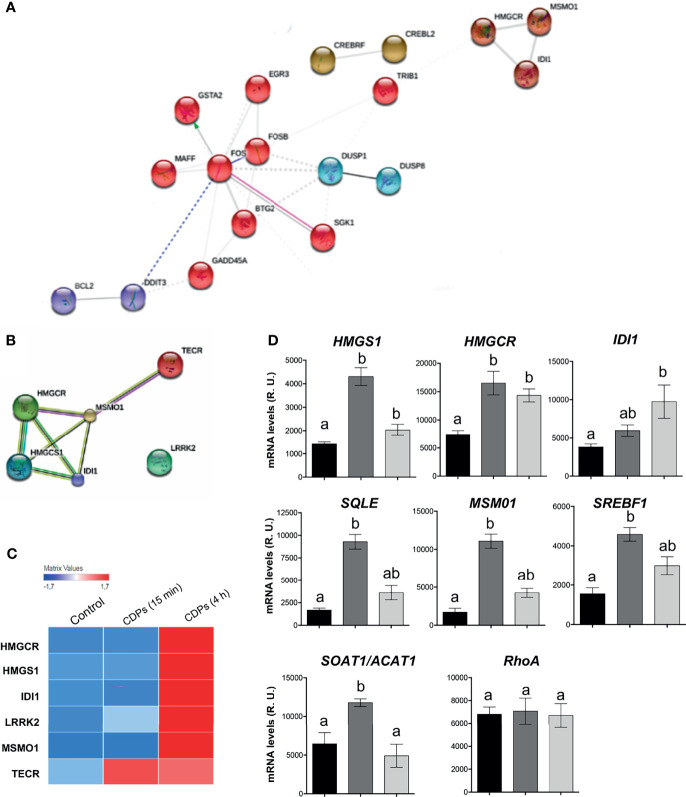
DEGs expression of the mevalonate and cholesterol pathways in HeLa cells exposed to CDPs. **(A, B)** Close up of STRING network, showing the top prioritized DEGs of the mevalonate and cholesterol pathways. Each cluster represents a set of highly connected nodes and is illustrated in a discrete color. **(C)** Heat map of mevalonate and cholesterol-pathway-associated DEGs of transcriptome. **(D)** Relative expression of mRNA levels of genes from the mevalonate and cholesterol pathways determined through RT-PCR of CDPs-exposed HeLa cells at 15 min and 4 h. The products were examined using amplification curves. The amounts of transcript were obtained at 20 cycles of the exponential amplification curve and expressed as relative units using the Image J software. Data represent the means ± SEM from three replicates each. One-way analysis of variance (ANOVA) was carried out, with Bonferroni *post-hoc* test; statistical significance (*P ≤* 0.05) of differences between treatments is indicated with lowercase letters.

The network interactions between the clusters and functional categories, identified DEGs categorized in the terpenoid backbone biosynthesis (**Figures 3C, D**). In this STRING network, the main clusters observed contained DETFs such as *FOS*, *FOSB*, *MAFF*, *BTG2*, *SGK1*, *GADD45A*, *GSTA2*, *EGR3*, *DDIT3*, *BCL2*, *DUSP1*, and *DUSP8*, interacting with the DEGs *TRIB1* and subsequently with the *HMGCR*, *MOSMO1*, and *IDI1* (**Figure 7A**); interestingly, the last genes are part of the mevalonate/cholesterol pathways. The transcriptome and bioinformatic analyses of specific pathway components shown here, revealed that six pivotal genes in the mevalonate/cholesterol pathways experienced significant changes after CDPs exposure. These key mevalonate genes showing altered expression encodes for: 3-hydroxy-3-methylglutaryl coenzyme A synthase (HMGCS1), 3-hydroxy-3-methylglutaryl CoA reductase (HMGCR), isopentenyl-diphosphate-delta-isomerase 1 (IDI1), methylsterol monooxygenase 1 (MSMO1), very-long-chain enoyl-CoA reductase (TECR), and leucine-rich repeat serine/threonine-protein kinase 2 (LRRK2) (**Figures 7B, C**). Overall, these findings suggest that the mevalonate/cholesterol pathway regulation was modified in HeLa cells by the bacterial CDPs-exposure.

**Figure 8:**
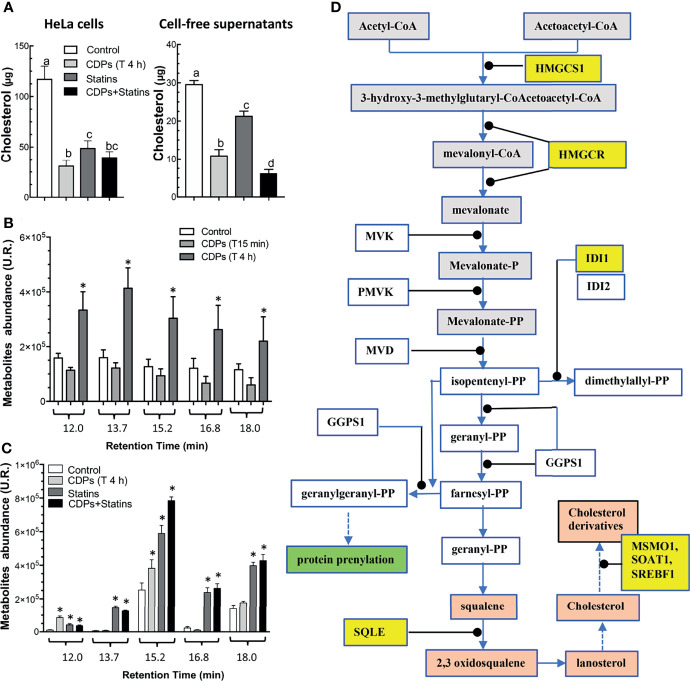
Content of cholesterol and intermediary metabolites of the mevalonate pathway in HeLa cells exposed to CDPs. **(A)** Determination of total cholesterol amounts in HeLa cells and cell-free supernatants of cultures of HeLa cells exposed for 4 h to CDPs and statins (mervastatin), determined by spectrophotometry at 505 nm. **(B, C)** Organic acids of the mevalonate pathway were determined by GC-MS analysis using cell-free supernatants of cultures of HeLa cells exposed for 4 h to CDPs and statins (mervastatin). Compounds are shown at the respective retention time as follows: trans-3-hydroxyhex-4-enoic (12.0 min), 3,5-dihydroxyhexanoic 1,5 lactone (13.7 min), trans-5-hydroxyhex-2-enoic (15.2 min), 4-hydroxy-6-methyl-2-pyrone (16.8 min), and 5-hydroxy-3-ketohexanoic (18.0 min). Compound identification was carried out as described by (52). Data represent the means ± SE of three independent assays. A one-way ANOVA with a Bonferroni *post-hoc* test was used to compare treatment times with respect to the control (time 0). Significant differences (*P* < 0.05) *vs* control is denoted with an asterisk or lowercase letters. **(D)** Mevalonate and cholesterol pathways showing the genes with modified expression by the exposure of HeLa cells to CDPs are highlighted in yellow.

To evaluate the participation of the cholesterol synthesis pathway in the antiproliferative mechanism of the CDPs in HeLa cells, cholesterol amounts were determined in cells exposed to bacterial CDPs. In the CDPs-treated HeLa cultures, the amounts of cholesterol in both, cellular pellets and cell-free supernatants decreased significantly, at similar levels to those obtained with mervastatin-treated cells (**Figure 8A**), confirming the blockage of cholesterol synthesis as an antiproliferative mechanism. Deepening in the mechanism involved, we determined the expression of genes of the mevalonate and cholesterol pathways in HeLa cells exposed to CDPs by RT-PCR assays. Findings showed that the genes *HMGCS1*, *HMGCR*, and *IDI1* from the mevalonate pathway, increased their transcription levels in CDPs-treated HeLa cells (**Figure 7D**), in agreement with the transcriptome results (**Figure 7C**). In addition, the expression of the genes *SQLE* (encoding for squalene epoxidase), *MSMO1* (encoding for sterol-C4-methyl oxidase), *SOAT1/ACAT1* (encoding for cholesterol acyltransferase), and *SREBF1* (encoding for sterol regulatory element-1 transcription factor), belonging to the sterols synthesis pathway, also increased by the CDPs treatment (**Figure 7D**). The differences in the expression levels were more clearly observed at short CDPs exposure time (15 min), but was also observed at 4 h CDPs-exposure. As a control, the RhoA gene that is up-regulated when the HMGCR is inhibited by statins was used (53), showing unmodified expression by CDPs-exposure.

In agreement with the up-regulation of genes of the mevalonate/cholesterol pathways observed in cancer patients also as metabolites accumulation in the urinary organic acids profiles occurred in patients with mitochondrial HMGCS deficiency (52). In our study, the cultures of HeLa cells exposed to bacterial CDPs showed an increase in metabolites corresponding to organic acids of the mevalonate pathway (**Figure 8B**). The metabolites identified by mass fragmentation profiles [as reported by Pitt et al. (52); see *Material and Methods*] were: trans-3-hydroxyhex-4-enoic, 3,5-dihydroxyhexanoic 1,5 lactone, trans-5-hydroxyhex-2-enoic, 4-hydroxy-6-methyl-2-pyrone, and 5-hydroxy-3-ketohexanoic, corresponded to intermediary compounds of the mevalonate pathway. The maximum accumulation of organic acids of the mevalonate metabolism was found after a long time-period of CDPs exposure (4 h). These findings were further confirmed when mervastatin (statin) was used as inhibitor of the HMGCR enzyme and with the combination of statin and CDPs addition; in this case the mevalonate metabolites accumulation was synergistic (**Figure 8C**). Conversely, cholesterol determination in both, cellular pellets and cell-free supernatants of CDPs-treated HeLa cultures, decreased significantly as with mervastatin-treated cells (**Figure 8A**).

## Discussion

Treatment with drugs is the most common therapy for many types of cancer. Hence, it is important to unravel the molecular mechanisms of drug action. Drugs directly kill cancer cells by stopping the proliferation and by inducing a cellular response. Substances such as natural cyclodipeptides (2,5-diketopiperazines) that directly impact cell proliferation have a high potential for cancer treatment. The bacterium *P. aeruginosa* PAO1 produces preferentially the cyclodipeptides cyclo (l-Pro- l-Tyr), cyclo (l-Pro- lPhe), and cyclo (l-Pro- l-Val), whose ability to arrest the cell cycle at the G0-G1, transition has already been implicated in the inhibition of the proliferation of human HeLa cell line. The mechanism of bacterial CDPs on inhibition of proliferation of the HeLa cells model involves the inhibition of phosphorylation of protein-kinases, such as Akt-Ser473, mTOR-Ser2448, and p70S6K-Thr389, mediated by the mTORC1 and mTORC2 complexes (24). Thus, our aim was to obtain more robust evidences of the antiproliferative/cytotoxic effects of *P. aeruginosa* PAO1 CDPs in a human cervical cancer cell line through a transcriptome comparative analyses.

### General Analysis of Transcriptomic Study

In the cancer context, evidences indicate that cancer progression is related to dysregulation in the expression of a plethora of molecules, including TFs, representing approximately 20% of known oncogenes associated with the development and maintenance of cancer (54). The roles of transcriptional regulators, including transcription factors (TFs) and RNA molecules such as miRNA are strongly associated with cancer development and invasiveness (55, 56). Given the importance of gene expression during the development of cancers; several global gene expression profiles using RNA-Seq can provide insights into the regulatory genes and critical pathways involved in these diseases. Also it provides useful information about how the products of individual genes, or their combinations, could efficiently kill cancerous cells, as well as to identify molecular signatures and pathways, which allow the design of novel therapeutics to target a specific cancer type (57–59).

Our RNA-sequencing data identified 151 genes that were differentially expressed by effect of the CDPs-exposure, including 141 up-regulated and 10 down-regulated genes. The function of these DEGs was annotated and enriched by databases, such as the ChEA3 TF tool, ShinyGO, Common Pathways, and KEGG. The top 151 DEG-enriched were further investigated by bioinformatics analysis in the KEGG database in order to study in deep the molecular elements associated with the cytotoxic effect caused by the CDPs exposure on HeLa cell cultures (**Table 2**). Transcriptomic analysis of HeLa cells confirmed that exposure to CDPs leads to a significant modification of the gene expression profile, being particularly visible in genes involved in protein-kinase signal transduction pathways, some of them previously identified by proteomic studies (24). Importantly, in our transcriptomic study were also identified several genes encoding cancer-associated transcription factors that participate in processes such as epigenetics, DNA splicing, and damage response, as well as in regulation of miRNAs, circRNAs, and lncRNA, suggesting that modulation of these genes could contribute to the overall molecular mechanisms associated with the cytotoxic effect of the bacterial CDPs in the HeLa cell line.

Additionally, the computational analysis of TF enrichment presented here, according to the ChEA3 database and based on co-modified DEGs, revealed a prioritized list of transcription regulators potentially involved in a wide range of cellular processes related to transformation and malignant cell progression (**Table 3**). These TF included e.g., *ATRX*, *BCL6*, *EGR3*, *COL6A1*, *ANKDR12*, *HMGA2*, which are key members of AP-1 transcription factor such as *FOS*, *FOSB*, and *MAFF*. Thus, our findings show that the expression of transcripts in HeLa cell cultures were significantly modified by CDPs-exposure. With regards to functional Gene Ontology and pathways analyses, an interesting result was the prevalence of protein-kinases belonging to cancer-related signaling transduction pathways. We identified three uniquely up-regulated genes of the PI3K/Akt/mTOR signaling pathway, including *FNIP2*, *SGK1*, and *BCL2*; and the up-regulated genes of the Wnt pathway, including *APC*, *HLTF*, *MYH7B*, and *WDR37*. Meanwhile in the MAPK signaling pathway, four exclusive genes were up-regulated, *DDIT3*, *DUSP1*, *DUSP8*, and *GADD45A* (**Figure 3**).

The heatmap and STRING network showed five DETFs that have been described as biomarkers for cancer, including *BCL6*, *DDIT3*, *GADD45A*, *HMGA2*, and *ID2* (**Figure 4**), which genes are members of the aforementioned pathways and are also involved in the control of cell growth, proliferation, and cell death. In relation to the regulation of cell death *via* apoptosis, the anti-apoptotic B-cell lymphoma-2 (BCL-2) (54) and the B cell lymphoma 6 (BCL6), a transcriptional suppressor of BCL2 (60), the dual-specificity phosphatase 1 (DUSP1), also identified as MAPK phosphatase-1, a well-known inhibitor of the MAPK pathway (61), were also significantly up-regulated in response to CDPs.

It is well documented that the regulation of gene expression responds quickly to cellular signals. Primary response genes (PRGs) are expressed following a wide range of external stimuli, to modify the mechanisms of regulation involved in cell proliferation. The PRGs are classically grouped in two types: immediate early response genes (IEGs), and delayed primary response genes (DPRGs). The mRNA of IEGs can appear in cells within 5 to 60 min after the stimulus, whereas the DPRGs are induced later, around 4 h after the stimulus (62). Typical PRGs are TFs, such as *EGR3* and *FOS*, which are components of intracellular signal transduction pathways (e.g., MAPK phosphatases such as DUSP1and DUSP8) (63, 64). The HeLa cell line is a convenient model to explain how bacterial CDPs can act as a relatively brief stimulus (15 min), but also as a long-term signal (4 h) to alter gene expression through IEGs and DPRGs responses. In the current study, 18 genes (12%) corresponded to IEGs, while 133 genes (88%) were of the type DPRGs. This shows that hierarchically transcriptional programs and various signaling pathways were modulated. We also found three key representative genes to be up-regulated which are involved in the apoptotic signaling pathway in response to endoplasmic reticulum stress, including the DNA damage-inducible transcript 3 (*DDIT3*, known as *CHOP*) (65), the growth arrest and DNA-damage-inducible beta (*GADD45B*) (66), and the Glutathione-Specific Gamma-Glutamyl-cyclo-transferase, encoded by the *CHAC1* gene, an inducer of glutathione depletion - which is an important factor for apoptosis initiation and execution (67). Prolonged ER stress can lead to apoptosis through the activation of DDIT3/CHOP (68, 69).

The major signaling pathways affected by CDPs (**Figures 4**, **5**), also commonly activated in many physiological responses, are growth factor receptor tyrosine kinases (RTKs; e.g., the epidermal growth factor receptor, EGFR), small GTPases (e.g., RASL11A), serine/threonine kinases (e.g., STK36, DCLK and SGK1), cytoplasmic tyrosine kinases (e.g., TTBK2), lipid kinases (e.g., phosphoinositide 3-kinases, PI3Ks), as well as nuclear receptors (e.g., the estrogen receptor, ER), and components of developmental signaling pathways, such as Wnt (e.g., FZD4 and APC), TGF-β (e.g., ID2 and SMAD6), bone morphogenetic protein, G protein-coupled receptor (e.g., GPR176, CCDC88A/GIRDIN), Hedgehog (e.g., GAS1), PI3K/Akt/mTOR (e.g., SGK1), Hippo (e.g., CCN2), and Notch (e.g., HES1 and LFNG), as they could all modulate downstream signaling pathways, including transcription factors (e.g., HMGA2, FOS and EGR3), chromatin remodelers (e.g., ATRX), and cell cycle motors (e.g., CCNF cyclin).

In addition to the identification of the widely described DEGs and DETFs associated with several types of cancer and transduction signaling pathways, we also found a group of DEGs, including circRNAs, lncRNA, RNAs regulated by miRNAs, and TFs which are related to fundamental processes such as epigenetics, splicing, and DNA-damage response (**Supplementary Table S4**). From the main DEGs, some were assigned as RNAs, miRNAs-regulated and those associated with cancer and neurodegenerative diseases, i.e., *FAM135A*, *MTRNR2L2*, *PSD3*, *HMGA2*, *LIN7A*, and *MYH7B*; as well as circRNAs i.e., *ANKRD12* (recruitment of histone deacetylases), *APC* (involved in tumorigenesis), and *PSD3* (associated with obesity, type 2 diabetes, and cholesterologenesis), and *MTRNR2L10*; from the lncRNA type, *KTN1* (an anti-sense RNA involved in tumorigenesis and EMT). The TFs most commonly identified are related to fundamental processes of DNA modifications, which have been associated with cancer, chromatin remodeling, neurodegenerative diseases, and epigenetics, i.e., *ATRX*, *ASCL2*, *ATOH8*, *BCL6*, *BTG2*, *C11orf65*, *CCDC148*, *CCDC88A*, *CDC6*, *CENPE*, *CEP170-2*, *CHD9*, *CLK4*, *CWC22*, *DGCR8*, *FOXP2*, *HFM1*, *HIST1H4K*, *HLTF*, *ID2*, *KIF20*B, *KBTBD8*, *LMX1B*, *LINGO1*, *MAFF*, *NAA16*, *PHF1*, *PPIG*, *RFX1-2*, *SLF1*, *STK36*, *YOD1*, *ZFY*, and *ZNF236* (**Tables 2**, **3**).

Our current and past findings also suggest that, in addition to some of the mentioned pathways, which are inhibited by bacterial CDPs, such as the AMPK and PI3K/Akt/mTOR signaling transduction networks (24), aimed at inhibiting the pathways that lead to cancer by altering a large number of cellular processes that lead to the orderly death of cancer cells. The data presented here will not only serve to guide future hypothesis-driven investigations aimed at identifying molecular targets for bacterial CDPs, but could also to be used to develop exposure biomarkers and/or to evaluate the therapeutic potential of CDPs.

### Implication of the Mevalonate and Sterol Pathway in the CDPs Cytotoxic Effect on HeLa Cells

Several studies have reported that cholesterol plays a critical role in the progression of numerous types of cancer (35, 70, 71). Disruption of fatty acids and cholesterol biosynthesis can also induce ER-stress (68). In agreement with this, increased activity of the mevalonate pathway has been implicated in cancers and aberrant protein prenylation which occurs when the pathway is highly up-regulated (72–74). In this sense, in breast cancer, mutations in P53 up-regulate components of the mevalonate pathway through the sterol regulatory element-binding protein (SREBP) family of transcription factors, increasing the flux in the mevalonate pathway of these mutants (75).

In our study, transcriptomics analysis and bioinformatics examination of DEGs revealed that six key genes in the mevalonate and cholesterol pathways were up-expressed in HeLa cells treated with CDPs (**Figures 5**, **7**), these were: *HMGCR*, *HMGCS1*, *IDI1*, *TECR*, *MSMO1*, and the transcription regulator *LRRK2* (**Figure 7C**), suggesting a targeting of the CDPs on these pathways, in addition to aim protein kinases as the molecular mechanism of inhibition of cell proliferation. Deepening in the transcriptome data base, it was found that the treatment of CDPs at 4 h of exposure increase significantly the *SREBF1* transcript counts from 4.2 in the control to 14.2 counts of expression in the CDPs exposure. In agreement to the transcriptomic analysis, mRNA quantitation by RT-PCR assays showed that the genes *HMGS1*, *HMGCR*, *IDI1*, *SQLE*, *MSMO1*, *SREBF1*, and *SOAT1*, were over-expressed in HeLa cells following CDP exposure (**Figure 7D**). Therefore, our findings indicate that the enzymes HMGCR, HMGCS1, IDI1, MSMO1, TECR, and SQLE of these pathways could be candidates for the modulation by bacterial CDPs, probably by the control of the gene expression through the TFs LRRK2 and SREBF1, or perhaps through protein-kinases that hierarchically control critical molecular interconnections between AMPK and PI3K/Akt/mTOR signaling. In this sense, it has been demonstrated through microarrays and the RT-qPCR gene expression analysis, that cell treatments with HMGCR inhibitors, differentially induces the expression of *HMGCR*, *HMGCS1* and *IDI1* genes in breast cancer cell lines (76). Also, the increase of the HMGCR activity in cancer proliferating cells provokes an increase in the content and consumption of cholesterol (77, 78). Interestingly, AMPK pathway has been reported to phosphorylate and suppress the activity of the sterol regulatory element binding proteins (SREBP-1c and -2), transcriptional factors that control the expression of enzymes of the mevalonate pathway (79). AMPK was also described as a direct regulator of the phosphorylation of HMGCR, causing a decrease in enzymatic activity (80).

Cholesterol is the precursor of steroid hormones, bile acids, and oxysterols, but also modifies proteins that are covalently attached to Hedgehog proteins and to smoothened, signaling pathways that play critical roles in embryonic development and tumorigenesis. On the other hand, blocking the mevalonate pathway through inhibition of HMGCR cause apoptosis by P38 activation and suppressing activation of the Akt and Erk pathways by reducing the metabolic products downstream of the HMG-CoA reductase reaction (36). Thus, mechanistically, drug inhibition of HMGCR can decrease cholesterol synthesis, thereby attenuating cell proliferation, suppressing tumor progression, and inducing cell senescence by negatively regulating growth-promoting signals, including RAS, PI3K/AKT, RAF/MEK/ERK1/2, Hippo, and Wnt/β-catenin signaling cascades (81–83). In addition, HMCS2 deficiency causes the accumulation of organic acids, which can be detected in the urine of patients with metabolic disorders associated with hypoglycemia and increased accumulation of fatty acids metabolites (52). Furthermore, increased activity of the mevalonate pathway has been linked to cancers and aberrant protein prenylation which occurs when the pathway is highly up-regulated (72–74).

Interestingly, our findings showed that both, genes of mevalonate and cholesterol pathways (*HMGS1*, *HMGCR*, *IDI1*, *SQLE*, *MSMO1*, *SREBF1*, and *SOAT1*), are positively regulated by bacterial CDPs on HeLa cell cultures (**Figure 7**). Accordingly, intermediary metabolites of the mevalonate pathway were accumulated in the supernatants of the HeLa culture media, but conversely, the cholesterol content significantly decreased in both cells and supernatants (**Figure 8A**). The decreased amounts of cholesterol observed in HeLa cells treated with CDPs showed a similar behavior than the statins treatment, indicating that the deficiency in cholesterol induces its autosynthesis and those of the mevalonate precursors, causing their accumulation as a result of the allosteric regulation of the HMGCR (limiting enzyme of the cholesterol biosynthesis) (**Figure 8D**). The HMGCR induction, positively promotes the gene expression of the biosynthetic genes involved in both, the mevalonate and cholesterol synthesis pathways. Thus, the results showed here suggest that the mechanism of antiproliferation/cytotoxicity in the HeLa cells by the bacterial CDPs involves the inhibition of the HMGCR enzyme in a mechanism similar to that of the statins, probably by a mechanism dependent of inhibition of enzymatic activity by phosphorylation.

Some meta-analysis studies suggest that the mechanism of antiproliferation by statins is beneficial for cell survival and cancer-specific cell survival; however, the effect could be pleiotropic, affecting other mechanisms such as protein prenylation, tumor cell proliferation and migration, inhibiting Ras signaling, inducing apoptosis through inhibition of Akt phosphorylation, and consequently mTOR down-regulation at other cellular level (84). Thus, cholesterol represents a precursor for the hormones estrogens and androgens, involved in the modulation of cell proliferation, migration, invasion and apoptosis in different cancers (78). Here, we found that although the genes of the mevalonate pathway were up-regulated by CDPs-exposure, the cholesterol amounts were significantly diminished in the cultures, confirming the importance of cholesterol and its precursors in cell cancer proliferation, invasiveness, and apoptosis.

## Conclusions

The transcriptomic study in the HeLa cell line supports the anti-proliferative/cytotoxic effects of the CDPs shown previously, providing new knowledge on the molecular mechanisms, deepening in the elucidation of the signal pathways involved in the anti-neoplastic effects of the bacterial CDPs using the HeLa cell line as a model of human cervical cancer. The findings suggest that as part of the cytotoxic effects of the bacterial CDPs on HeLa cells, these compounds transduce the signal through the PI3K-Akt-mTOR pathway to multiple transcription factors. This study also demonstrates the impact of CDPs on the expression of genes of the mevalonate/cholesterol pathways, which are essential for cell proliferation, finding a correlation between gene expression and accumulation of metabolites of the mevalonate pathway, but decreasing the amounts of cholesterol. This fact suggests a blockage of sterols synthesis as an additional mechanism of death induction by CDPs in the HeLa cell line. Our study also highlights the potential of CDPs as anti-neoplastic drugs, genes that could be used as therapeutic targets and/or biomarkers for the treatment and monitoring of this type of cervical cancer.

## Data Availability Statement

The datasets presented in this study can be found in online repositories. The names of the repository/repositories and accession number(s) can be found below: https://www.ncbi.nlm.nih.gov/sra, Bioproject ID PRJNA725963.

## Authors Contributions

Conception and design: JC-G. Development of methodology: PL-M, LH-P, JG-C, EM-C, and LM-A. Analysis and interpretation of data: PL-M, JG-C, JL-B, AG-G, and JC-G. Writing, review, and/or revision of the manuscript: PL-M, JL-B, AG-G, and JC-G. Administrative, technical, or material support: JL-B, AG-G, and JC-G. Study supervision: AG-G and JC-G. All authors read and approved the final manuscript.

## Funding

This study received funding from the Consejo Nacional de Ciencia y Tecnologia (CONACYT-México) grant No 256119, Marcos Moshinsky Fundation grant 2014-16, Universidad Michoacana de San Nicolás de Hidalgo grant CIC-2.14, and DGAPA PAPIIT UNAM Grant No 209420. PL-M and EM-C were awarded with a CONACYT Postdoctoral Fellowship. The funders were not involved in the study design, collection, analysis, interpretation of data, the writing of this article or the decision to submit it for publication.

## Conflict of Interest

The authors declare that the research was conducted in the absence of any commercial or financial relationships that could be construed as a potential conflict of interest.

## Publisher’s Note

All claims expressed in this article are solely those of the authors and do not necessarily represent those of their affiliated organizations, or those of the publisher, the editors and the reviewers. Any product that may be evaluated in this article, or claim that may be made by its manufacturer, is not guaranteed or endorsed by the publisher.

## References

[1] Arbyn M Weiderpass E Bruni L de Sanjosé S Saraiya M Ferlay J. Estimates of Incidence and Mortality of Cervical Cancer in 2018: A Worldwide Analysis. Lancet Glob Health (2020) 8(2):e191–203. doi: 10.1016/S2214-109X(19)30482-6 PMC7025157 31812369

[2] Olusola P Banerjee HN Philley JV Dasgupta S. Human Papilloma Virus-Associated Cervical Cancer and Health Disparities. Cells (2019) 8(6):622. doi: 10.3390/cells8060622 PMC6628030 31234354

[3] Kuguyo O Tsikai N Thomford NE Magwali T Madziyire MG Nhachi CFB. Genetic Susceptibility for Cervical Cancer in African Populations: What Are the Host Genetic Drivers? Omics A J Integr Biol (2018) 22(7):468–83. doi: 10.1089/omi.2018.0075 30004844

[4] Wang X Chen M Zhou J Zhang X. HSP27, 70 and 90, Anti-Apoptotic Proteins, in Clinical Cancer Therapy (Review). Int J Oncol (2014) 45(1):18–30. doi: 10.3892/ijo.2014.2399 24789222

[5] Crosbie EJ Einstein MH Franceschi S Kitchener HC. Human Papillomavirus and Cervical Cancer. Lancet (2013) 382(9895):889–99. doi: 10.1016/S0140-6736(13)60022-7 23618600

[6] Tewari KS Sill MW Long HJ Penson RT Huang H Ramondetta LM. Improved Survival With Bevacizumab in Advanced Cervical Cancer. N Engl J Med (2014) 370(8):734–43. doi: 10.1056/NEJMoa1309748 PMC4010094 24552320

[7] Franke TF Hornik CP Segev L Shostak GA Sugimoto C. PI3K/Akt and Apoptosis: Size Matters. Oncogene (2003) 22(56):8983–98. doi: 10.1038/sj.onc.1207115 14663477

[8] LoPiccolo J Blumenthal GM Bernstein WB Dennis PA. Targeting the PI3K/Akt/mTOR Pathway: Effective Combinations and Clinical Considerations. Drug Resist Updates (2008) 11(1-2):32–50. doi: 10.1016/j.drup.2007.11.003 PMC2442829 18166498

[9] Aoki M Fujishita T. Oncogenic Roles of the PI3K/AKT/mTOR Axis. Curr Topics Microbiol Immunol (2017) 407:153–89. doi: 10.1007/82_2017_6 28550454

[10] Lee H. Phosphorylated mTOR Expression Profiles in Human Normal and Carcinoma Tissues. Dis Markers (2017) 2017(1397063):8. doi: 10.1155/2017/1397063 PMC5555007 28831205

[11] Pópulo H Lopes JM Soares P. The mTOR Signalling Pathway in Human Cancer. Int J Mol Sci (2012) 13(2):1886–918. doi: 10.3390/ijms13021886 PMC3291999 22408430

[12] Crino PB. The mTOR Signalling Cascade: Paving New Roads to Cure Neurological Disease. Nat Rev Neurol (2016) 12(7):379–92. doi: 10.1038/nrneurol.2016.81 27340022

[13] Kim AL Back JH Chaudhary SC Zhu Y Athar M Bickers DR. SOX9 Transcriptionally Regulates mTOR-Induced Proliferation of Basal Cell Carcinomas. J Invest Dermatol (2018) 138(8):1716–25. doi: 10.1016/j.jid.2018.01.040 PMC6056318 29550418

[14] Fan QW Cheng C Knight ZA Haas-Kogan D Stokoe D James CD. EGFR Signals to mTOR Through PKC and Independently of Akt in Glioma. Sci Signaling (2009) 2(55):ra4. doi: 10.1126/scisignal.2000014 PMC2793677 19176518

[15] Razmara M Heldin C-H Lennartsson J. Platelet-Derived Growth Factor-Induced Akt Phosphorylation Requires mTOR/Rictor and Phospholipase C-γ1, Whereas S6 Phosphorylation Depends on mTOR/Raptor and Phospholipase D. Cell Commun Signal (2013) 11(1):3. doi: 10.1186/1478-811X-11-3 23311350 PMC3560233

[16] Sarbassov DD Guertin DA Ali SM Sabatini DM. Phosphorylation and Regulation of Akt/PKB by the rictor-mTOR Complex. Science (2005) 307(5712):1098–101. doi: 10.1126/science.1106148 15718470

[17] Alessi DR Pearce LR García-Martínez JM. New Insights Into mTOR Signaling: Mtorc2 and Beyond. Sci Signal (2009) 2(67):pe27. doi: 10.1126/scisignal.267pe27 19383978

[18] O’Reilly KE Rojo F She QB Solit D Mills GB Smith D. mTOR Inhibition Induces Upstream Receptor Tyrosine Kinase Signaling and Activates Akt. Cancer Res (2006) 66(3):1500–8. doi: 10.1158/0008-5472.CAN-05-2925 PMC3193604 16452206

[19] Yang G Murashige DS Humphrey SJ James DE. A Positive Feedback Loop Between Akt and Mtorc2 *via* SIN1 Phosphorylation. Cell Rep (2015) 12(6):937–43. doi: 10.1016/j.celrep.2015.07.016 26235620

[20] Li Q Song XM Ji YY Jiang H Xu LG. The Dual Mtorc1 and Mtorc2 Inhibitor AZD8055 Inhibits Head and Neck Squamous Cell Carcinoma Cell Growth *In Vivo* and *In Vitro* . Biochem Biophys Res Commun (2013) 440(4):701–6. doi: 10.1016/j.bbrc.2013.09.130 24103749

[21] Slotkin EK Patwardhan PP Vasudeva SD De Stanchina E Tap WD Schwartz GK. MLN0128, an ATP-Competitive mTOR Kinase Inhibitor With Potent *In Vitro* and *In Vivo* Antitumor Activity, as Potential Therapy for Bone and Soft-Tissue Sarcoma. Mol Cancer Ther (2015) 14(2):395–406. doi: 10.1158/1535-7163.MCT-14-0711 25519700 PMC4332837

[22] Petrossian K Nguyen D Lo C Kanaya N Somlo G Cui YX. Use of Dual mTOR Inhibitor MLN0128 Against Everolimus-Resistant Breast Cancer. Breast Cancer Res Treat (2018) 170(3):499–506. doi: 10.1007/s10549-018-4779-x 29623577 PMC6026053

[23] Bhagwat SV Gokhale PC Crew AP Cooke A Yao Y Mantis C. Preclinical Characterization of OSI-027, a Potent and Selective Inhibitor of Mtorc1 and Mtorc2: Distinct From Rapamycin. Mol Cancer Ther (2011) 10(8):1394–406. doi: 10.1158/1535-7163.MCT-10-1099 21673091

[24] Hernández-Padilla L Reyes de la Cruz H Campos-García J. Antiproliferative Effect of Bacterial Cyclodipeptides in the HeLa Line of Human Cervical Cancer Reveals Multiple Protein Kinase Targeting, Including Mtorc1/C2 Complex Inhibition in a TSC1/2-Dependent Manner. Apoptosis (2020) 25(9–10):632–47. doi: 10.1007/s10495-020-01619-z 32617785

[25] Durán-Maldonado MX Hernández-Padilla L Gallardo-Pérez JC Díaz-Pérez AL Martínez-Alcantar L Reyes de la Cruz H. Bacterial Cyclodipeptides Target Signal Pathways Involved in Malignant Melanoma. Front Oncol (2020) 10:1111. doi: 10.3389/fonc.2020.01111 32793477 PMC7393205

[26] Brauns SC Milne P Naudé R Van De Venter M. Selected Cyclic Dipeptides Inhibit Cancer Cell Growth and Indace Apoptosis in HT-29 Colon Cancer Cells. Anticancer Res (2004) 24(3a):1713–9. 15274345

[27] Furukawa T Akutagawa T Funatani H Uchida T Hotta Y Niwa M. Cyclic Dipeptides Exhibit Potency for Scavenging Radicals. Bioorg Med Chem (2012) 20(6):2002–9. doi: 10.1016/j.bmc.2012.01.050 22356736

[28] Nishanth Kumar S Dileep C Mohandas C Nambisan B Ca J. Cyclo(D-Tyr-D-Phe): A New Antibacterial, Anticancer, and Antioxidant Cyclic Dipeptide From Bacillus Sp. N Strain Associated With a Rhabditid Entomopathogenic Nematode. J Pept Sci (2014) 20(3):173–85. doi: 10.1002/psc.2594 24353056

[29] Vázquez-Rivera D González O Guzmán-Rodríguez J Díaz-Pérez AL Ochoa-Zarzosa A López-Bucio J. Cytotoxicity of Cyclodipeptides From Pseudomonas Aeruginosa PAO1 Leads to Apoptosis in Human Cancer Cell Lines. BioMed Res Int (2015) 2015:197608. doi: 10.1155/2015/197608 25821788 PMC4363556

[30] Hernández-Padilla L Vázquez-Rivera D Sánchez-Briones LA Díaz-Pérez AL Moreno-Rodríguez J Moreno-Eutimio MA. The Antiproliferative Effect of Cyclodipeptides From Pseudomonas Aeruginosa PAO1 on HeLa Cells Involves Inhibition of Phosphorylation of Akt and S6k Kinases. Molecules (2017) 22(6):1024. doi: 10.3390/molecules22061024 PMC6152764 28632179

[31] Liskamp RMJ Rijkers DTS Kruijtzer JAW Kemmink J. Peptides and Proteins as a Continuing Exciting Source of Inspiration for Peptidomimetics. ChemBioChem (2011) 12(11):1626–53. doi: 10.1002/chin.201147233 21751324

[32] Menegatti S Hussain M Naik AD Carbonell RG Rao BM. mRNA Display Selection and Solid-Phase Synthesis of Fc-Binding Cyclic Peptide Affinity Ligands. Biotechnol Bioeng (2013) 110(3):857–70. doi: 10.1002/bit.24760 23108907

[33] Sánchez-Hernández I Baquero P Calleros L Chiloeches A. Dual Inhibition of (V600E)BRAF and the PI3K/AKT/mTOR Pathway Cooperates to Induce Apoptosis in Melanoma Cells Through a MEK-Independent Mechanism. Cancer Lett (2012) 314(2):244–55. doi: 10.1016/j.canlet.2011.09.037 22056813

[34] Yamauchi Y Rogers MA. Sterol Metabolism and Transport in Atherosclerosis and Cancer. Front Endocrinol (2018) 9:509. doi: 10.3389/fendo.2018.00509 PMC6157400 30283400

[35] Chimento A Casaburi I Avena P Trotta F De Luca A Rago V. Cholesterol and its Metabolites in Tumor Growth: Therapeutic Potential of Statins in Cancer Treatment. Front Endocrinol (2019) 9:807. doi: 10.3389/fendo.2018.00807 PMC6348274 30719023

[36] Qi XF Zheng L Lee KJ Kim DH Kim CS Cai DQ. HMG-CoA Reductase Inhibitors Induce Apoptosis of Lymphoma Cells by Promoting ROS Generation and Regulating Akt, Erk and P38 Signals *via* Suppression of Mevalonate Pathway. Cell Death Dis (2013) 4(2):e518. doi: 10.1038/cddis.2013.44 23449454 PMC3734846

[37] González O Ortíz-Castro R Díaz-Pérez C Díaz-Pérez AL Magaña-Dueñas V López-Bucio J. Non-Ribosomal Peptide Synthases From Pseudomonas Aeruginosa Play a Role in Cyclodipeptide Biosynthesis, Quorum-Sensing Regulation, and Root Development in a Plant Host. Microbial Ecol (2017) 73(3):616–29. doi: 10.1007/s00248-016-0896-4 27900439

[38] Martin M. Cutadapt Removes Adapter Sequences From High-Throughput Sequencing Reads. EMBnet.journal (2011) 17(1):10–2. doi: 10.14806/ej.17.1.200

[39] Langmead B Salzberg S. Fast gapped-read alignment with Bowtie 2. Nat Methods (2012) 9:357–9. 10.1038/nmeth.1923 PMC3322381 22388286

[40] Li H Handsaker B Wysoker A Fennell T Ruan J Homer N. The Sequence Alignment/Map Format and SAMtools. Bioinformatics (2009) 25(16):2078–9. doi: 10.1093/bioinformatics/btp352 PMC2723002 19505943

[41] Quinlan AR Hall IM. BEDTools: A Flexible Suite of Utilities for Comparing Genomic Features. Bioinformatics (2010) 26(6):841–2. doi: 10.1093/bioinformatics/btq033 PMC2832824 20110278

[42] Tarazona S Furió-Tarí P Turrà D Di Pietro A Nueda MJ Ferrer A. Data Quality Aware Analysis of Differential Expression in RNA-Seq With NOISeq R/Bioc Package. Nucleic Acids Res (2015) 43(21):e140. doi: 10.1093/nar/gkv711 26184878 PMC4666377

[43] Kolde R Vilo J. GOsummaries: An R Package for Visual Functional Annotation of Experimental Data. F1000Res (2015) 4:574. doi: 10.12688/f1000research.6925.1 26913188 PMC4743157

[44] Fernandez NF Gundersen GW Rahman A Grimes ML Rikova K Hornbeck P. Clustergrammer, a Web-Based Heatmap Visualization and Analysis Tool for High-Dimensional Biological Data. Sci Data (2017) 4:170151. doi: 10.1038/sdata.2017.151 28994825 PMC5634325

[45] James DH. Panther. Grand Street (1995) 51:178–91. doi: 10.2307/25007829

[46] Kanehisa M Sato Y Kawashima M Furumichi M Tanabe M. KEGG as a Reference Resource for Gene and Protein Annotation. Nucleic Acids Res (2016) 44(1):457–62. doi: 10.1093/nar/gkv1070 PMC4702792 26476454

[47] Cerami EG Gross BE Demir E Rodchenkov I Babur Ö. Anwar N. Pathway Commons, a Web Resource for Biological Pathway Data. Nucleic Acids Res (2011) 39(1):685–90. doi: 10.1093/nar/gkq1039 PMC3013659 21071392

[48] Slenter DN Kutmon M Hanspers K Riutta A Windsor J Nunes N. WikiPathways: A Multifaceted Pathway Database Bridging Metabolomics to Other Omics Research. Nucleic Acids Res (2018) 46(1):661–7. doi: 10.1093/nar/gkx1064 PMC5753270 29136241

[49] Ge SX Jung D Jung D Yao R. ShinyGO: A Graphical Gene-Set Enrichment Tool for Animals and Plants. Bioinformatics (2020) 36(8):2628–9. doi: 10.1093/bioinformatics/btz931 PMC7178415 31882993

[50] Szklarczyk D Gable AL Lyon D Junge A Wyder S Huerta-Cepas J. STRING V11: Protein-Protein Association Networks With Increased Coverage, Supporting Functional Discovery in Genome-Wide Experimental Datasets. Nucleic Acids Res (2019) 47(1):607–13. doi: 10.1093/nar/gky1131 PMC6323986 30476243

[51] Keenan AB Torre D Lachmann A Leong AK Wojciechowicz ML Utti V. ChEA3: Transcription Factor Enrichment Analysis by Orthogonal Omics Integration. Nucleic Acids Res (2019) 47(1):212–24. doi: 10.1093/nar/gkz446 PMC6602523 31114921

[52] Pitt JJ Peters H Boneh A Yaplito-Lee J Wieser S Hinderhofer K. Mitochondrial 3-Hydroxy-3-Methylglutaryl-CoA Synthase Deficiency: Urinary Organic Acid Profiles and Expanded Spectrum of Mutations. J Inherit Metab Dis (2015) 38(3):459–66. doi: 10.1007/s10545-014-9801-9 25511235

[53] Gan Y Wang J Coselli J Wang XL. Synergistic Induction of Apoptosis by HMG-CoA Reductase Inhibitor and Histone Deacetylases Inhibitor in HeLa Cells. Biochem Biophys Res Commun (2008) 365(2):386–92. doi: 10.1016/j.bbrc.2007.11.002 PMC2151206 17996726

[54] Adams CM Clark-Garvey S Porcu P Eischen CM. Targeting the Bcl-2 Family in B Cell Lymphoma. Front Oncol (2019) 8:00636. doi: 10.3389/fonc.2018.00636 PMC6331425 30671383

[55] Chen Z Han Y Song C Wei H Chen Y Huang K. Systematic Review and Meta-Analysis of the Prognostic Significance of microRNAs in Cervical Cancer. Oncotarget (2017) 9(24):17141–8. 10.18632/oncotarget.23839 PMC5908312 29682211

[56] Rodríguez-Enríquez S Marín-Hernández Á Gallardo-Pérez JC Pacheco-Velázquez SC Belmont-Díaz JA Robledo-Cadena DX. Transcriptional Regulation of Energy Metabolism in Cancer Cells. Cells (2019) 8(10):1225. doi: 10.3390/cells8101225 PMC6830338 31600993

[57] Berg KCG Eide PW Eilertsen IA Johannessen B Bruun J Danielsen SA. Multi-Omics of 34 Colorectal Cancer Cell Lines - A Resource for Biomedical Studies. Mol Cancer (2017) 16:116. doi: 10.1186/s12943-017-0691-y 28683746 PMC5498998

[58] Ghandi M Huang FW Jané-Valbuena J Kryukov GV Lo CC McDonald ER. Next-Generation Characterization of the Cancer Cell Line Encyclopedia. Nature (2019) 569(7757):503–8. doi: 10.1038/s41586-019-1186-3 PMC6697103 31068700

[59] Yang X Kui L Tang M Li D Wei K Chen W. High-Throughput Transcriptome Profiling in Drug and Biomarker Discovery. Front Genet (2020) 11:00019. doi: 10.3389/fgene.2020.00019 PMC7013098 32117438

[60] Ci W Polo JM Cerchietti L Shaknovich R Wang L Shao NY. The BCL6 Transcriptional Program Features Repression of Multiple Oncogenes in Primary B Cells and is Deregulated in DLBCL. Blood (2009) 113(22):5536–48. doi: 10.1182/blood-2008-12-193037 PMC2689052 19307668

[61] Peng HZ Yun Z Wang W Ma BA. Dual Specificity Phosphatase 1 has a Protective Role in Osteoarthritis Fibroblast-Like Synoviocytes *via* Inhibition of the MAPK Signaling Pathway. Mol Med Rep (2017) 16:8441–7. doi: 10.3892/mmr.2017.7617 28983624

[62] Tullai JW Schaffer ME Mullenbrock S Sholder G Kasif S Cooper GM. Immediate-Early and Delayed Primary Response Genes are Distinct in Function and Genomic Architecture. J Biol Chem (2007) 282(33):23981–95. doi: 10.1074/jbc.M702044200 PMC2039722 17575275

[63] Levin WJ Press MF Gaynor RB Sukhatme VP Boone TC Reissmann PT. Expression Patterns of Immediate Early Transcription Factors in Human non-Small Cell Lung Cancer. The Lung Cancer Study Group. Oncogene (1995) 11(7):1261–9. 7478546

[64] Healy S Khan P Davie JR. Immediate Early Response Genes and Cell Transformation. Pharmacol Ther (2013) 137(1):64–77. doi: 10.1016/j.pharmthera.2012.09.001 22983151

[65] Zinszner H Kuroda M Wang XZ Batchvarova N Lightfoot RT Remotti H. CHOP is Implicated in Programmed Cell Death in Response to Impaired Function of the Endoplasmic Reticulum. Genes Dev (1998) 12:982–95. doi: 10.1101/gad.12.7.982 PMC316680 9531536

[66] Wang L Xiao X Li D Chi Y Wei P Wang Y. Abnormal Expression of GADD45B in Human Colorectal Carcinoma. J Trans Med (2012) 10:215. doi: 10.1186/1479-5876-10-215 PMC3495754 23110778

[67] Mungrue IN Pagnon J Kohannim O Gargalovic PS Lusis AJ. CHAC1/MGC4504 Is a Novel Proapoptotic Component of the Unfolded Protein Response, Downstream of the ATF4-ATF3-CHOP Cascade. J Immunol (2009) 182(1):466–76. doi: 10.4049/jimmunol.182.1.466 PMC2846782 19109178

[68] Jakobsen CH Størvold GL Bremseth H Follestad T Sand K Mack M. DHA Induces ER Stress and Growth Arrest in Human Colon Cancer Cells: Associations With Cholesterol and Calcium Homeostasis. J Lipid Res (2008) 49(19):2089–100. doi: 10.1194/jlr.M700389-JLR200 PMC2533412 18566476

[69] Rozpedek W Pytel D Mucha B Leszczynska H Diehl JA Majsterek I. The Role of the PERK/Eif2α/ATF4/CHOP Signaling Pathway in Tumor Progression During Endoplasmic Reticulum Stress. Curr Mol Med (2016) 16(6):533–44. doi: 10.2174/1566524016666160523143937 PMC5008685 27211800

[70] Kuzu OF Noory MA Robertson GP. The Role of Cholesterol in Cancer. Cancer Res (2016) 76(8):2063–70. doi: 10.1158/0008-5472.CAN-15-2613 PMC5813477 27197250

[71] Ding X Zhang W Li S Yang H. The Role of Cholesterol Metabolism in Cancer. Am J Cancer Res (2019) 9(2):219–27. PMC6405981 30906624

[72] Clendening JW Pandyra A Li Z Boutros PC Martirosyan A Lehner R. Exploiting the Mevalonate Pathway to Distinguish Statin-Sensitive Multiple Myeloma. Blood (2010) 115(23):4787–97. doi: 10.1182/blood-2009-07-230508 20360469

[73] Berndt N Hamilton AD Sebti SM. Targeting Protein Prenylation for Cancer Therapy. Nat Rev Cancer (2011) 11:775–91. doi: 10.1038/nrc3151 PMC4037130 22020205

[74] Gruenbacher G Thurnher M. Mevalonate Metabolism Governs Cancer Immune Surveillance. OncoImmunology (2017) 6(10):e1342917. doi: 10.1080/2162402X.2017.1342917 29123952 PMC5665080

[75] Freed-Pastor WA Mizuno H Zhao X Langerød A Moon SH Rodriguez-Barrueco R. Mutant P53 Disrupts Mammary Tissue Architecture *via* the Mevalonate Pathway. Cell (2012) 148(1-2):244–58. doi: 10.1016/j.cell.2011.12.017 PMC3511889 22265415

[76] Kimbung S Lettiero B Feldt M Bosch A Borgquist S. High Expression of Cholesterol Biosynthesis Genes Is Associated With Resistance to Statin Treatment and Inferior Survival in Breast Cancer. Oncotarget (2016) 7:59640–51. doi: 10.18632/oncotarget.10746 PMC5312337 27458152

[77] Azrolan NI Coleman PS. A Discoordinate Increase in the Cellular Amount of 3-Hydroxy-3-Methylglutaryl-CoA Reductase Results in the Loss of Rate-Limiting Control Over Cholesterogenesis in a Tumour Cell-Free System. Biochem J (1989) 258(2):421–5. doi: 10.1042/bj2580421 PMC1138379 2705993

[78] Huang B Song B-L Xu C. Cholesterol Metabolism in Cancer: Mechanisms and Therapeutic Opportunities. Nat Metab (2020) 2(2):132–41. doi: 10.1038/s42255-020-0174-0 32694690

[79] Li Y Xu S Mihaylova MM Zheng B Hou X Jiang B. AMPK Phosphorylates and Inhibits SREBP Activity to Attenuate Hepatic Steatosis and Atherosclerosis in Diet-Induced Insulin-Resistant Mice. Cell Metab (2011) 13(4):376–88. doi: 10.1016/j.cmet.2011.03.009 PMC3086578 21459323

[80] Burg JS Espenshade PJ. Regulation of HMG-CoA Reductase in Mammals and Yeast. Prog Lipid Res (2011) 50(4):403–10. doi: 10.1016/j.plipres.2011.07.002 PMC3184313 21801748

[81] Wang Z Wu Y Wang H Zhang Y Mei L Fang X. Interplay of Mevalonate and Hippo Pathways Regulates RHAMM Transcription *via* YAP to Modulate Breast Cancer Cell Motility. Proc Natl Acad Sci USA (2014) 111(1):E89–98. doi: 10.1073/pnas.1319190110 PMC3890879 24367099

[82] Warita K Warita T Beckwitt CH Schurdak ME Vazquez A Wells A. Statin-Induced Mevalonate Pathway Inhibition Attenuates the Growth of Mesenchymal-Like Cancer Cells That Lack Functional E-Cadherin Mediated Cell Cohesion. Sci Rep (2014) 4:7593. doi: 10.1038/srep07593 25534349 PMC4274516

[83] Ampuero J Romero-Gomez M. Prevention of Hepatocellular Carcinoma by Correction of Metabolic Abnormalities: Role of Statins and Metformin. World J Hepatol (2015) 7(8):1105–11. doi: 10.4254/wjh.v7.i8.1105 PMC4450187 26052399

[84] Casaburi I Chimento A De Luca A Nocito M Sculco S Avena P. Cholesterol as an Endogenous Errα Agonist: A New Perspective to Cancer Treatment. Front Endocrinol (2018) 9:525. doi: 10.3389/fendo.2018.00525 PMC6141749 30254608

