# Supplementary material for: Transcriptomics Reveals the Mevalonate and Cholesterol Pathways Blocking as Part of the Bacterial Cyclodipeptides Cytotoxic Effects in HeLa Cells of Human Cervix Adenocarcinoma
Source: Front Oncol. 2022 Mar 14;12:790537. doi: 10.3389/fonc.2022.790537 (PMC8964019; doi:10.3389/fonc.2022.790537)
Supplement: Supplementary file 1 [file Presentation_1.pptx]

## Slide 1
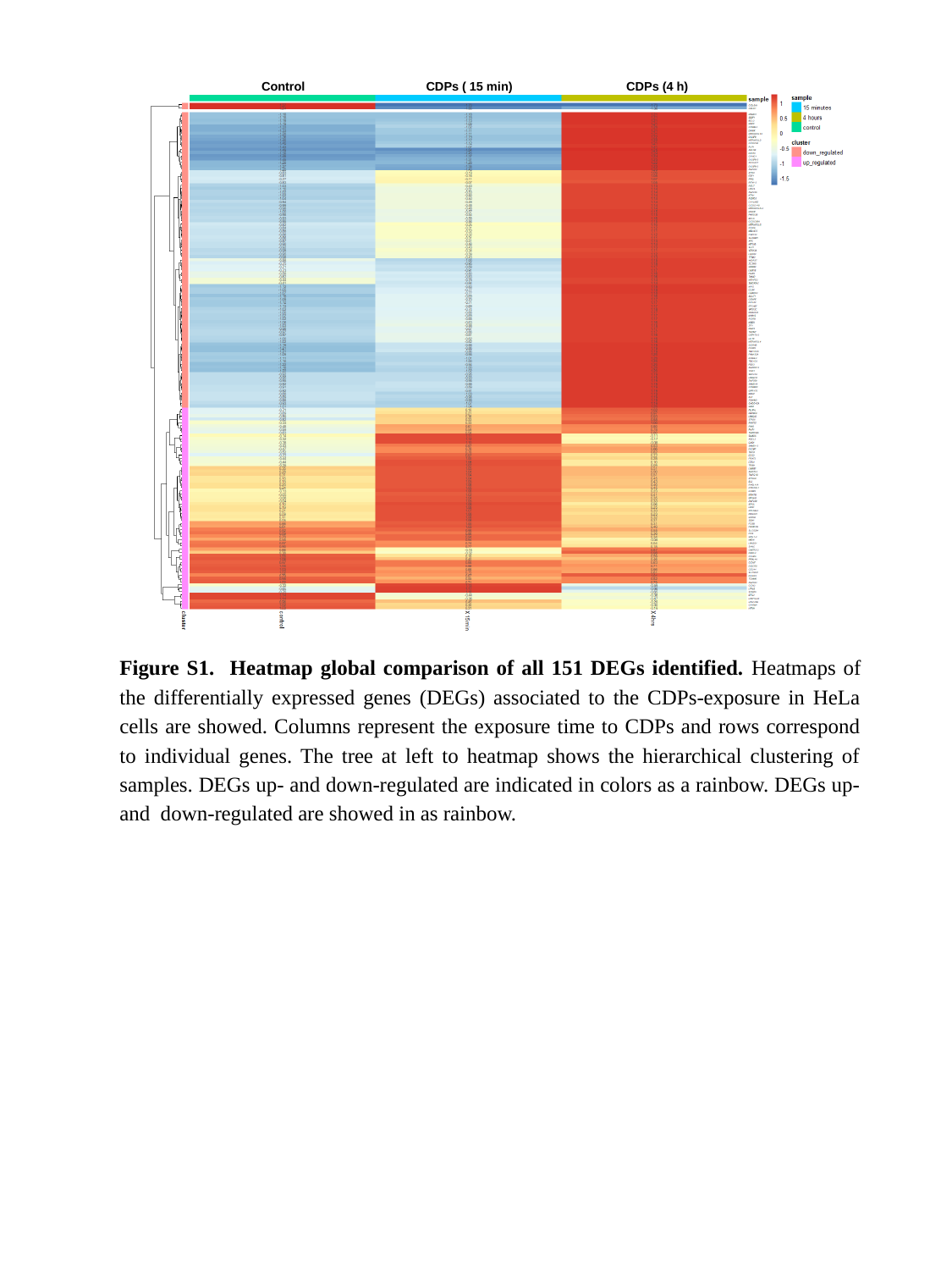

CDPs (4 h)
CDPs ( 15 min)
Control
Figure S1. Heatmap global comparison of all 151 DEGs identified. Heatmaps of the differentially expressed genes (DEGs) associated to the CDPs-exposure in HeLa cells are showed. Columns represent the exposure time to CDPs and rows correspond to individual genes. The tree at left to heatmap shows the hierarchical clustering of samples. DEGs up- and down-regulated are indicated in colors as a rainbow. DEGs up- and down-regulated are showed in as rainbow.
