# Supplementary material for: Transcriptomics Reveals the Mevalonate and Cholesterol Pathways Blocking as Part of the Bacterial Cyclodipeptides Cytotoxic Effects in HeLa Cells of Human Cervix Adenocarcinoma
Source: Front Oncol. 2022 Mar 14;12:790537. doi: 10.3389/fonc.2022.790537 (PMC8964019; doi:10.3389/fonc.2022.790537)
Supplement: Supplementary file 2 [file Presentation_2.pptx]

## Slide 1
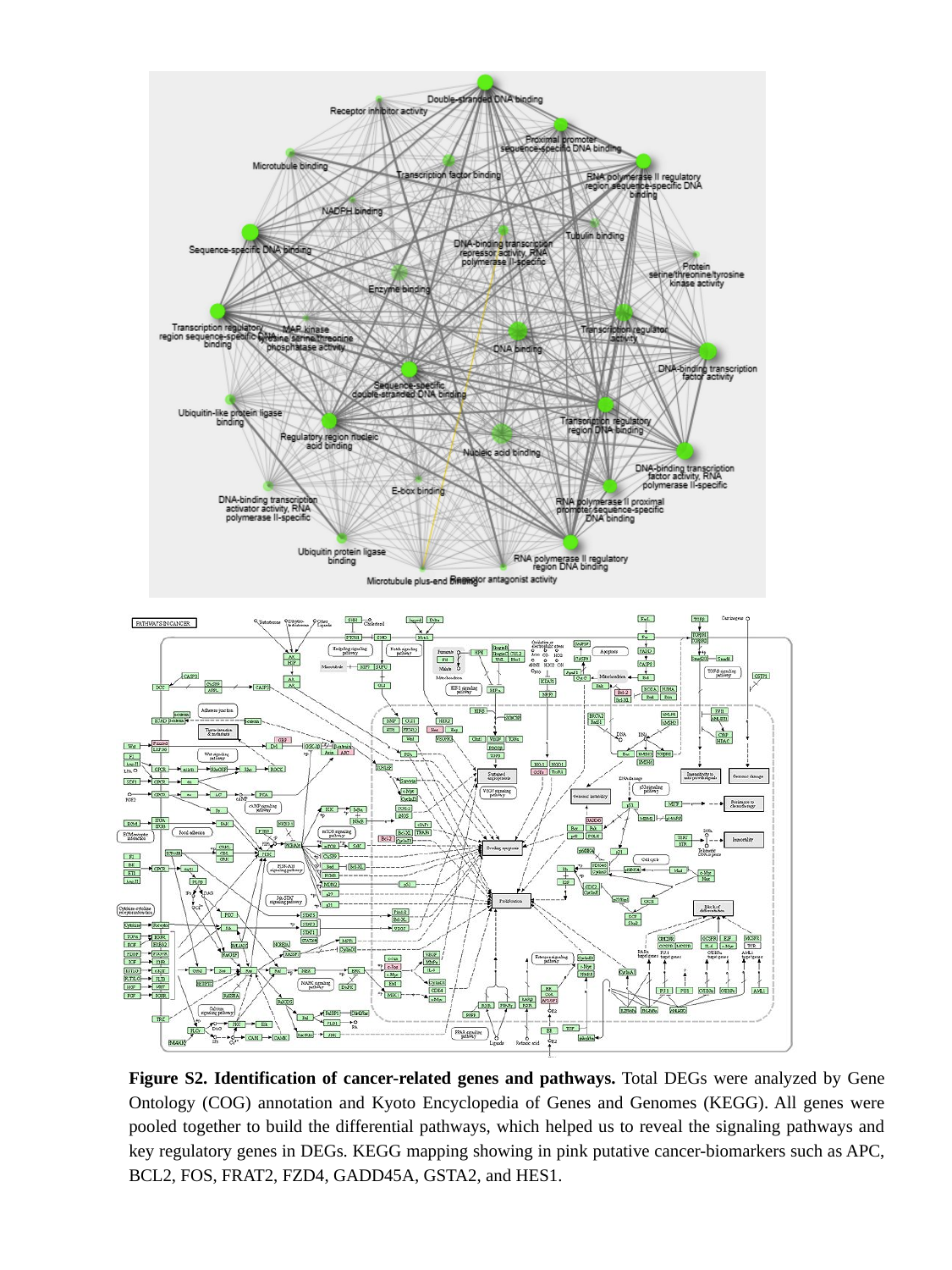

Figure S2. Identification of cancer-related genes and pathways. Total DEGs were analyzed by Gene Ontology (COG) annotation and Kyoto Encyclopedia of Genes and Genomes (KEGG). All genes were pooled together to build the differential pathways, which helped us to reveal the signaling pathways and key regulatory genes in DEGs. KEGG mapping showing in pink putative cancer-biomarkers such as APC, BCL2, FOS, FRAT2, FZD4, GADD45A, GSTA2, and HES1.
