# Supplementary material for: Transcriptomics Reveals the Mevalonate and Cholesterol Pathways Blocking as Part of the Bacterial Cyclodipeptides Cytotoxic Effects in HeLa Cells of Human Cervix Adenocarcinoma
Source: Front Oncol. 2022 Mar 14;12:790537. doi: 10.3389/fonc.2022.790537 (PMC8964019; doi:10.3389/fonc.2022.790537)
Supplement: Supplementary file 3 [file Presentation_3.pptx]

## Slide 1
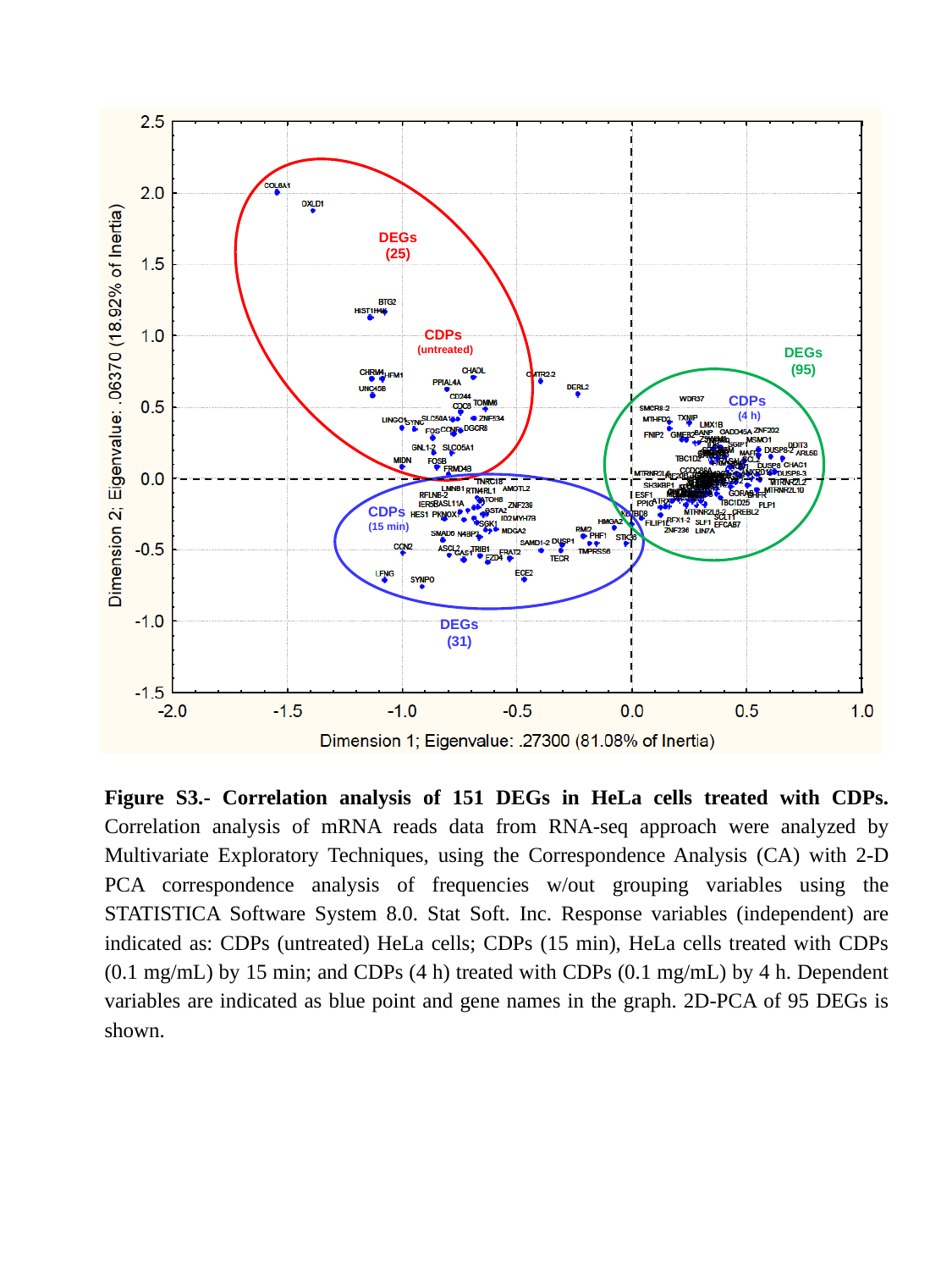

CDPs
(untreated)
CDPs
(4 h)
CDPs
(15 min)
DEGs
(25)
DEGs
(95)
DEGs
(31)
Figure S3.- Correlation analysis of 151 DEGs in HeLa cells treated with CDPs. Correlation analysis of mRNA reads data from RNA-seq approach were analyzed by Multivariate Exploratory Techniques, using the Correspondence Analysis (CA) with 2-D PCA correspondence analysis of frequencies w/out grouping variables using the STATISTICA Software System 8.0. Stat Soft. Inc. Response variables (independent) are indicated as: CDPs (untreated) HeLa cells; CDPs (15 min), HeLa cells treated with CDPs (0.1 mg/mL) by 15 min; and CDPs (4 h) treated with CDPs (0.1 mg/mL) by 4 h. Dependent variables are indicated as blue point and gene names in the graph. 2D-PCA of 95 DEGs is shown.
