# Supplementary material for: Transcriptomics Reveals the Mevalonate and Cholesterol Pathways Blocking as Part of the Bacterial Cyclodipeptides Cytotoxic Effects in HeLa Cells of Human Cervix Adenocarcinoma
Source: Front Oncol. 2022 Mar 14;12:790537. doi: 10.3389/fonc.2022.790537 (PMC8964019; doi:10.3389/fonc.2022.790537)
Supplement: Supplementary file 4 [file Presentation_4.pptx]

## Slide 1
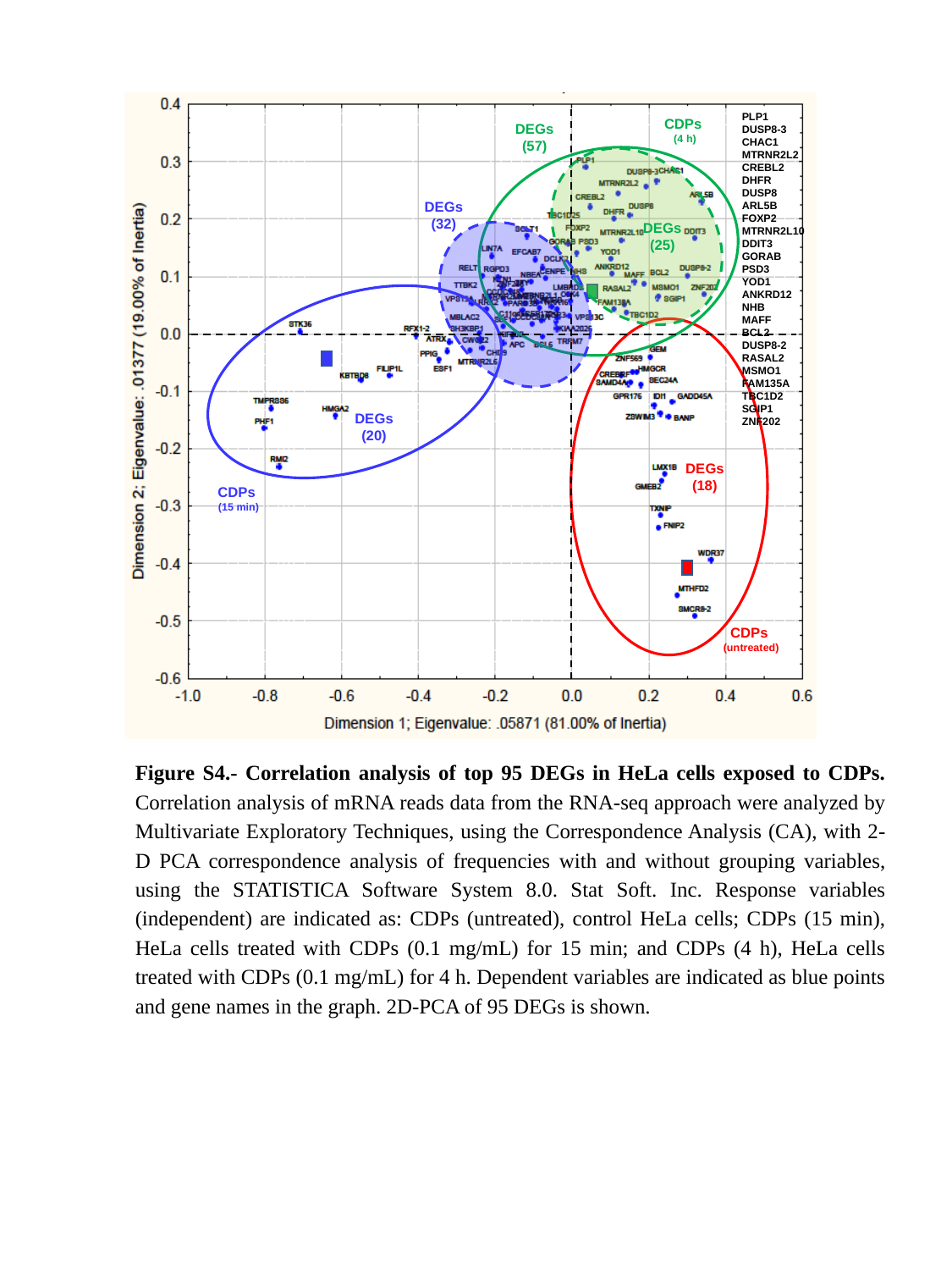

CDPs
(4 h)
DEGs
(57)
DEGs
(32)
DEGs
(25)
DEGs
(20)
DEGs
(18)
CDPs
(15 min)
CDPs
(untreated)
PLP1
DUSP8-3
CHAC1
MTRNR2L2
CREBL2
DHFR
DUSP8
ARL5B
FOXP2
MTRNR2L10
DDIT3
GORAB
PSD3
YOD1
ANKRD12
NHB
MAFF
BCL2
DUSP8-2
RASAL2
MSMO1
FAM135A
TBC1D2
SGIP1
ZNF202
Figure S4.- Correlation analysis of top 95 DEGs in HeLa cells exposed to CDPs. Correlation analysis of mRNA reads data from the RNA-seq approach were analyzed by Multivariate Exploratory Techniques, using the Correspondence Analysis (CA), with 2-D PCA correspondence analysis of frequencies with and without grouping variables, using the STATISTICA Software System 8.0. Stat Soft. Inc. Response variables (independent) are indicated as: CDPs (untreated), control HeLa cells; CDPs (15 min), HeLa cells treated with CDPs (0.1 mg/mL) for 15 min; and CDPs (4 h), HeLa cells treated with CDPs (0.1 mg/mL) for 4 h. Dependent variables are indicated as blue points and gene names in the graph. 2D-PCA of 95 DEGs is shown.
