# Supplementary material for: Transcriptomics Reveals the Mevalonate and Cholesterol Pathways Blocking as Part of the Bacterial Cyclodipeptides Cytotoxic Effects in HeLa Cells of Human Cervix Adenocarcinoma
Source: Front Oncol. 2022 Mar 14;12:790537. doi: 10.3389/fonc.2022.790537 (PMC8964019; doi:10.3389/fonc.2022.790537)
Supplement: Supplementary file 5 [file Presentation_5.pptx]

## Slide 1
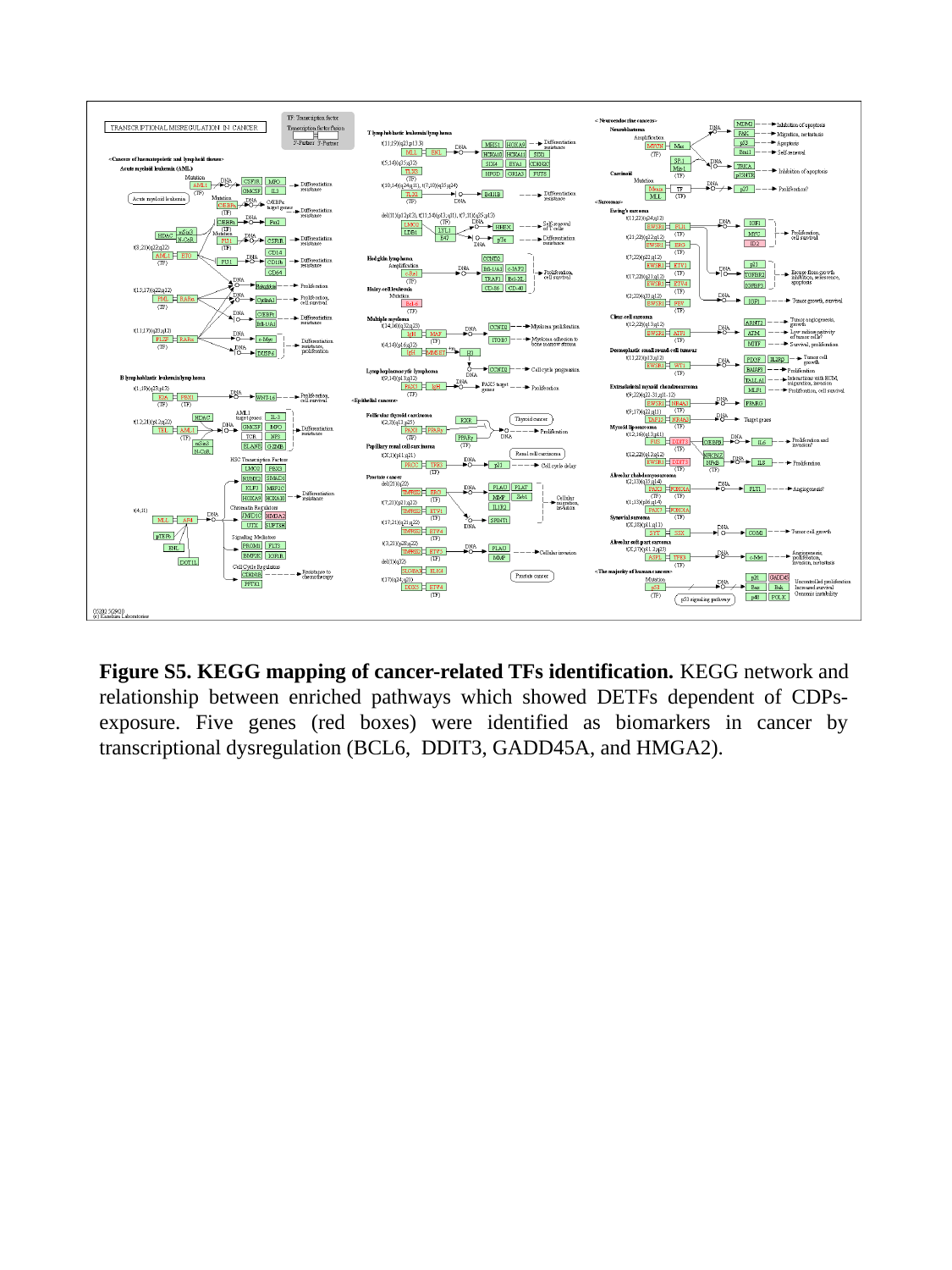

Figure S5. KEGG mapping of cancer-related TFs identification. KEGG network and relationship between enriched pathways which showed DETFs dependent of CDPs-exposure. Five genes (red boxes) were identified as biomarkers in cancer by transcriptional dysregulation (BCL6, DDIT3, GADD45A, and HMGA2).
