# Supplementary material for: Transcriptomics Reveals the Mevalonate and Cholesterol Pathways Blocking as Part of the Bacterial Cyclodipeptides Cytotoxic Effects in HeLa Cells of Human Cervix Adenocarcinoma
Source: Front Oncol. 2022 Mar 14;12:790537. doi: 10.3389/fonc.2022.790537 (PMC8964019; doi:10.3389/fonc.2022.790537)
Supplement: Supplementary file 6 [file Table_1.docx]

Table S1.- Oligonucleotides sequences used in RT-qPCR analysis.

_________________________________________________________________________

Gene Sequence

_________________________________________________________________________

*ATRX* Fw-GTAGACAAGCCAGCCAGGAG, Rv-CATGAGGTGACCCAGTGTTG

*ANKRD12* Fw-TGGCTGCTATTCGAGGAGAT, Rv-TCATGGAGTGGAGTGTCATCA

*BCL6*  Fw-CATGCAGAGATGTGCCTCCACA Rv-TCAGAGAAGCGGCAGTCACACT

*COL6A1*  Fw-TCAAGAGCCTGCAGTGGATG Rv-TGGACACTTCTTGTCTATGCAG

*EGR3* Fw-GACTCGGTAGTCCATTACAATCAG Rv-AGTAGGTCACGGTCTTGTTGCC

*DGR8*  Fw-CAAGCAGGAGACATCGGACAAG Rv-CACAATGGACATCTTGGGCTTC *GAPDH* Fw-CTTCATTGACCTCAACTACATGG Rv-GTCTTCTGGGTGGCAGTGATG

*SREBF1* Fw-ACTTCTGGAGGCATCGCAAGCA Rv-AGGTTCCAGAGGAGGCTACAAG

*HMGCS1* Fw-AAGTCACACAAGATGCTACACCG Rv-TCAGCGAAGACATCTGGTGCCA

*HMGR* Fw-TGCAGAGCAATAGGTCTTGGTG Rv-TCGAGCCAGGCTTTCACTTC

*IDI1* Fw-GCCGCAGACTGTGCTCAAAGC Rv-CCTGTTGCTTGTCGAGGTGGTT

*SQLE* Fw-CTCCAAGTTCAGGAAAAGCCTGG Rv-GAGAACTGGACTCGGGTTAGCT

*SOAT1/ACAT1* Fw-CCAGCCACTAAGCTTGGTTCCA Rv-GTAGGAGCTTGTCCTTCACCTC

*MSM01* Fw-GCTGCCTTTGATTTGTGGAACCT Rv-CTGCACAACCAAAGCATCTTGCC

*RhoA* Fw-TGGAAAGACATGCTTGCTCAT Rv-GCCTCAGGCGATCATAATCTTC

_______________________________________________________________________________________
