# Supplementary material for: Transcriptomics Reveals the Mevalonate and Cholesterol Pathways Blocking as Part of the Bacterial Cyclodipeptides Cytotoxic Effects in HeLa Cells of Human Cervix Adenocarcinoma
Source: Front Oncol. 2022 Mar 14;12:790537. doi: 10.3389/fonc.2022.790537 (PMC8964019; doi:10.3389/fonc.2022.790537)
Supplement: Supplementary file 7 [file Table_2.docx]

**Table S2. DEGs differentially identified.**

| **ctrl_vs_15 min** | **ctrl_vs_15 min ctrl_vs_4 h** | **ctrl_vs_4 h** | **ctrl_vs_4 h 15 min_vs_4 h** | **15 min_vs_4 h** | **ctrl_vs_15 min**  **15 min_vs_4 h** |
| --- | --- | --- | --- | --- | --- |
| CMTR2-2 | BTG2 | APC | ANKRD12 | AMOTL2 | SMCR8-2 |
| DERL2 |  | ATRX |  | ASCL2 |  |
| DUSP1 | COL6A1 | BCL6 | ARL5B | ATOH8 |  |
| ECE2 |  | C11orf65 |  | BANP |  |
| FILIP1L | LIN7A | CCDC148 | BCL2 | CREBRF |  |
| HMGA2 |  | CCDC88A |  | DUSP8-2 |  |
| KBTBD8 |  | CCNF | CCN2 | FNIP2 |  |
| LMBRD2 |  | CD244 |  | FRAT2 |  |
| LRRK2 |  | CDC6 | CHAC1 | FZD4 |  |
| MBLAC2 |  | CENPE |  | GADD45A |  |
| MTRNR2L1 |  | CEP170-2 | DDIT3 | GAS1 |  |
| PHF1 |  | CHADL |  | GEM |  |
| RMI2 |  | CHD9 | DHFR | GMEB2 |  |
| SAMD1-2 |  | CHRM4 |  | GPR176 |  |
| STK36 |  | CLK4 | DUSP8 | GSTA2 |  |
| TECR |  | CREBL2 |  | HES1 |  |
| TMPRSS6 |  | CWC22 | DUSP8-3 | HMGCR |  |
|  |  | DCLK2 |  | ID2 |  |
|  |  | DGCR8 | FAM135A | IDI1 |  |
|  |  | EFCAB7 |  | IER5L |  |
|  |  | EGR3 | FOS | LFNG |  |
|  |  | ESF1 |  | LMNB1 |  |
|  |  | FOXP2 | FOSB | LMX1B |  |
|  |  | GNL1-2 |  | MDGA2 |  |
|  |  | GORAB | FRMD4B | MSMO1 |  |
|  |  | HIST1H4K |  | MTHFD2 |  |
|  |  | HLTF | HFM1 | MYH7B |  |
|  |  | KIAA2026 |  | N4BP2 |  |
|  |  | KIF20B | LINGO1 | PKNOX1 |  |
|  |  | KTN1 |  | RASL11A |  |
|  |  | MTRNR2L6 | MAFF | RFLNB-2 |  |
|  |  | MTRNR2L6-2 |  | RTN4RL1 |  |
|  |  | MYRIP | MIDN | SAMD4A |  |
|  |  | NAA16 |  | SEC24A |  |
|  |  | NBEA | MTRNR2L10 | SGIP1 |  |
|  |  | NHS |  | SGK1 |  |
|  |  | OXLD1 | MTRNR2L2 | SMAD6 |  |
|  |  | PARD3B |  | SYNPO |  |
|  |  | PLP1 | RASAL2 | TBC1D2 |  |
|  |  | PPIAL4A |  | TNRC18 |  |
|  |  | PPIG | SYNC | TRIB1 |  |
|  |  | PSD3 |  | TXNIP |  |
|  |  | RELT | UNC45B | WDR37 |  |
|  |  | RFX1-2 |  | ZC3H6 |  |
|  |  | RGPD3 | YOD1 | ZCCHC7 |  |
|  |  | RORB |  | ZNF202 |  |
|  |  | SCLT1 |  | ZNF239 |  |
|  |  | SH3KBP1 |  | ZNF569 |  |
|  |  | SLC50A1 |  | ZSWIM3 |  |
|  |  | SLCO5A1 |  |  |  |
|  |  | SLF1 |  |  |  |
|  |  | TBC1D25 |  |  |  |
|  |  | TOMM6 |  |  |  |
|  |  | TRPM7 |  |  |  |
|  |  | TTBK2 |  |  |  |
|  |  | VPS13A |  |  |  |
|  |  | VPS13C |  |  |  |
|  |  | ZFY |  |  |  |
|  |  | ZNF236 |  |  |  |
|  |  | ZNF534 |  |  |  |
