# Supplementary material for: Transcriptomics Reveals the Mevalonate and Cholesterol Pathways Blocking as Part of the Bacterial Cyclodipeptides Cytotoxic Effects in HeLa Cells of Human Cervix Adenocarcinoma
Source: Front Oncol. 2022 Mar 14;12:790537. doi: 10.3389/fonc.2022.790537 (PMC8964019; doi:10.3389/fonc.2022.790537)
Supplement: Supplementary file 10 [file Table_5.docx]

**Table S5. DEGs UP-REGULATED by the CDPs exposure in HeLa cells.**

| **DEGs** | **mRNA reads**  **(T0, T15min, T4h)** | **Cellular process target** | **References** |
| --- | --- | --- | --- |
| ***AMOTL2*** | (662,1406,875) | Angiomtin-like protein 2; member of the mTORC2/AMOTL2/YAP pathway, associated with angiogenesis, tumorigenesis, invasiveness. | [[1](#_ENREF_1)] |
| ***ANKRD12**** | (32,70,548) | circRNA involven in tumor invasion, migration, metabolism.  Recruit of histone deacetylases (HDACs) to the p160 coactivators/nuclear receptor complex to inhibit ligand-dependent transactivation. | [[2](#_ENREF_2)] |
| ***ARL5B**** | (2,1,53) | trans-Golgi network small G protein, regulating the endosome-TGN transport, invasividad. | [[3](#_ENREF_3)]  [[4](#_ENREF_4)] |
| ***APC*** | (64,234,794) | circRNA involved in tumorigenesis, proliferation inhibitor, Wnt/β-catenin signaling pathway. | [[5](#_ENREF_5)] |
| ***ASCL2*** | (12,49,16) | The basic helix-loop-helix (bHLH) transcription factor ASCL2 associated with embriogensesis, development, CSC, carcinogenesis. | [[6](#_ENREF_6)] |
| ***ATOH8*** | (39,97,59) | Atonal homolog 8 (Atoh8) transcription factor of the helix-loop-helix (bHLH) family, proliferation-to-differentiation transition in embryonic development, and chondrocyte proliferation. | [[7](#_ENREF_7)] |
| ***ATRX*** | (83,454,1184) | Alpha thalassemia/mental retardation syndrome X-linked (ATRX), chromatin remodeling, telomere maintenance, gliomas tumorigenesis. | [[8](#_ENREF_8)] |
| ***BCL2**** | (6,8,87) | The B cell lymphoma-2 (Bcl-2) key regulator of apoptosis, implicated in cancer, neurodegenerative disorders, ischemia and autoimmune diseases. | [[9](#_ENREF_9)] |
| ***BCL6*** | (29,82,349) | B-cell lymphoma 6 (BCL6), transcription regulator of multiple cancer-related pathways, in DNA damage sensing, proliferation check points. | [[10](#_ENREF_10),[11](#_ENREF_11)]. |
| ***C11orf65*** | (23,91,335 | Telomere maintaining related with aging, disease, and mortality. | [[12](#_ENREF_12)] |
| ***CCDC148*** | (4,21,75) | Coiled-coil domain containing 148 (CCDC148) is a transcription regulator involved in tumorogenesis in vulvar carcinoma. | [[13](#_ENREF_13)] |
| ***CCDC88A*** | (38,126,514) | Coiled-coil domain containing 88A (CCDC88A) is a transcription regulator involved in migration and invasiveness through phosphorylation of Src and ERK1/2 and/or dephosphorylation of AMPK1. | [[14](#_ENREF_14)] |
| ***CCN2*** | (45,153,25) | CCN2 is member glycoproteins termed CCN proteins (acronym of Cyr61/CTGF/Nov; temened as Cellular Communication Network factor 2), implicated in several diseases, embryonic development, angiogenesis, chondrogenesis, osteogenesis, fibrosis, mechanotransduction and inflammation. | [[15](#_ENREF_15),[16](#_ENREF_16)] |
| ***CENPE*** | (3,13,61) | Centromere protein E (CENPE) is a centromere binding protein and mitotic kinesin, involved with tumorigenesis. | [[17](#_ENREF_17)] |
| ***CEP170-2*** | 3(4,107,464) | Centrosome-associated protein (CEP170) involved in meiosis. | [[18](#_ENREF_18)] |
| ***CHAC1**** | (3,9,147) | Glutathione-specific Gamma-glutamylcyclotransferase 1 (CHAC1), involved in degradation of glutathione enhancing necroptosis and ferroptosis in human triple negative breast cancer cells via the GCN2-eIF2α-ATF4 pathway. Osteoblast differentiation. | [[19](#_ENREF_19)]  [[20](#_ENREF_20)] |
| ***CHD9*** | (24,99,299) | Chromodomain helicase DNA binding protein 9 (CHD9), involved in chromatin structure in growing oocytes and totipotency. Oxidative stress, inflamation by Notch/NF-κB pathway. | [[21](#_ENREF_21)]. |
| ***CLK4*** | (14,39,209) | Cdc2-like kinases 4 (CLK4) involved in alternative gene splicing of cell proliferation, tumorigenesis, and angiogenesis. | [[22](#_ENREF_22)] |
| ***CREBRF*** | (33,35,267) | Ortholog to REPTOR, involved in tumor suppressor, proliferation, angiogenesis, obesity and diabetes, repress the glucocorticoid receptor. | [[23](#_ENREF_23)] |
| ***CREBL2**** | (1,7,50) | Ortholog to REPTOR-BP, cAMP response element binding protein (CREB), promotes differentiation, adipogenesis and lipogenesis by PPARγ and C/EBPα expression. It mediates part of the transcriptional induction caused by mTORC1 inhibition. | [[24](#_ENREF_24)] |
| ***CWC22*** | (13,58,176) | Spliceosomal protein CWC22 binds the core of exon junction complex (EJC), and orchestrates pre-mRNA splicing and eIF4A3 binding. | [[25](#_ENREF_25)] |
| ***DCLK2*** | (3,15,70) | Doublecortin-like kinases (DCLKs) expressed in adult retinal ganglion cells, survival and regeneration of injured neurons. Hypomethylation is highly frequent in chronic lymphocytic leukaemia. | [[26](#_ENREF_26)] |
| ***DDIT3**** | (183,102,3389) | Member of the C/EBP family, tumor suppressor associated with endoplasmic reticulum-stress response. | [[27](#_ENREF_27)] |
| ***DHFR**** | (4,15,131) | Chondrosarcoma (CS) proteins involved in pathogenesis, progression, and prognosis. | [[28](#_ENREF_28)] |
| ***DUSP1*** | (66,441,412) | Dual-specificity phosphatases 1 (DUSP1) expression is strongly upregulated upon several stimuli such as oxidative stress, hypoxia, growth factors, glucocorticoids, heat shock and UV. | [[29](#_ENREF_29)] |
| ***DUSP8**** | (3,9,94) | Dual-specificity phosphatases (DUSPs) plays role in phosphorylation-mediated signal transduction of MAPK signaling ranging from cell oxidative stress response, cell apoptosis and various human diseases. | [[30](#_ENREF_30)] |
| ***DUSP8-2**** | (5,3,70) | Dual-specificity phosphatases (DUSPs) suppress proliferation and migration. Involved in hypothalamic control of glucose homeostasis. | [[31](#_ENREF_31)] |
| ***DUSP8-3**** | (2,7,95) | Dual-specificity phosphatases (DUSPs) suppress proliferation and migration. Involved in hypothalamic control of glucose homeostasis. | [[31](#_ENREF_31)] |
| ***ECE2*** | (2,29,17) | Endothelin‐converting enzyme‐2 (ECE2), involved in neurodevelopment, neurodegeneration, Alzheimer disease. | [[32](#_ENREF_32)] |
| ***EFCAB7*** | (4,24,108) | Positively regulates the Hedgehog pathway depends on primary cilia in vertebrates, involved in Ellis van Creveld and Weyers syndromes. | [[33](#_ENREF_33)] |
| ***EGR3*** | (47,130,614) | Early growth response gene 3 (EGR3) is a central transcription factor, regulates an array of target genes that mediate critical neurobiological processes such as synaptic plasticity, memory, cognition, also as autoimmune diseases. | [[34](#_ENREF_34),[35](#_ENREF_35)] |
| ***ESF1*** | (30,155,396) | 18S rRNA factor 1 (ESF1) involved in development of the cranial neural crest cells. Strong upregulation of p53 signaling, apoptosis, and proliferation. | [[36](#_ENREF_36)] |
| ***FAM135A**** | (13,22,166) | Gene regulated by miRNAs, its involved in regulation of gene of multiple biological processes and diseases. Coxsackievirus B3 (CVB3)-associated myocarditis. | [[37](#_ENREF_37)] |
| ***FILIP1L*** | (14,88,171) | Filamin A interacting protein 1-like (FILIP1L) is an inhibitor of the WNT/β-catenin signaling, EMT, play a key role in ovarian cancer metastasis and chemoresistance. | [[38](#_ENREF_38)] |
| ***FOXP2**** | (7,35,201) | Transcriptional factor involved in brain development, promotes CSC, tumorigenesis, and metastasis. | [[39](#_ENREF_39)] |
| ***FRAT2*** | (9,55,32) | Frequently rearranged in advanced T-cell lymphomas 2 (FRAT2), regulator of Wnt/β-catenin pathway, tumorigenesis. | [[40](#_ENREF_40)] |
| ***FZD4*** | (6,34,16) | Frizzleds (FZDs) is unconventional G protein-coupled receptors, activates signal pathways such as Wnt/β and EMT. | [[41](#_ENREF_41)] |
| ***GADD45A*** | (641,369,4612) | Growth arrest and DNA-damage-inducible protein (GADD45A), involved in tumorigenesis and invasiveness. | [[42](#_ENREF_42)] |
| ***GAS1*** | (17,80,30) | Growth arrest-specific gene 1 (GAS1), inhibites proliferation, induced cell cycle arrest and promotes apoptosis by PI3K/Akt pathway. | [[43](#_ENREF_43)] |
| ***GORAB**** | (3,12,72) | Member of the golgin family of coiled-coil Golgi proteins. Involved in embryonic development, protein trafficking, glycosylation, cell growth, migration, and extracellular matrix formation. Negative regulator of AKT phosphorylation. | [[44](#_ENREF_44)]  [[45](#_ENREF_45)] |
| ***GPR176*** | (27,28,220) | G-protein-coupled receptors (GPCRs) glycosylated, which is involved in the anacardic acid-induced transcriptional response of human breast cancer cells. | [[46](#_ENREF_46)] |
| ***HES1*** | (44,101,48) | Transcriptional regulator Hairy and Enhancer of Split 1 (Hes1) is essential for governing neural cells brain development. | [[47](#_ENREF_47)] |
| ***HLTF*** | (39,137,595) | Helicase-like transcription factor (HLTF), involved in chromatin remodeling, DNA replication, and DNA repair. | [[48](#_ENREF_48)] [[48](#_ENREF_48)] |
| ***HMGA2*** | (9,58,87) | High motility group (HMG) protein family regulated by miRNA-195, played important roles in proliferation, metastasis, and EMT in lung cancer. | [[49](#_ENREF_49)]  [[50](#_ENREF_50)] |
| ***HMGCR*** | (67,70,573) | 3-hydroxy-3-methylglutaryl-coenzyme A reductase (HMGCR) involved in autoimmune-mediated necrotizing myopathy. | [[51](#_ENREF_51)] |
| ***ID2*** | (52,129,76) | The inhibitor of DNA binding/differentiation (Id) family are negative regulators of transcription factors with a basic helix-loop-helix motif. Four members (ID1 to ID4) play critical roles in angiogenesis, neurogenesis, tumorigenesis, and immune development, by regulating cell differentiation. | [[52](#_ENREF_52)] |
| ***IDI1*** | (19,14,136) | Isopentenyl-diphosphate-D-isomerase (IDI1) of the cholesterol synthesis pathway. | [[53](#_ENREF_53)] |
| ***IER5L*** | (273,665,254) | Immediate Early Response 5-Like (IER5L), involved in nutrient-responsive pathway in systemic and adipose inflamation. | [[54](#_ENREF_54)] |
| ***KBTBD8*** | (7,51,88) | KBTBD8 ubiquitin ligase with CUL3 forms the biquitin ligase complex in pluripotent stem cells. Selectively regulates PKM1 through Erk1/2 and Aurora A kinases. | [[55](#_ENREF_55)]  [[56](#_ENREF_56)] |
| ***KIAA2026*** | (20,56,269) | Unknown function, involved in gene fusions neoplasia-associated mutations sarcomas. | [[57](#_ENREF_57)] |
| ***KIF20B*** | (17,61,219) | Member of the Kinesin-6 family required for efficient cytokinesis, in cerebral cortex growth and midbody maturation of neural stem cells. | [[58](#_ENREF_58)] [[58](#_ENREF_58)] |
| ***KTN1*** | (32,192,693) | KTN1 antisense RNA 1 (KTN1-AS1), tumor-related long noncoding RNAs (lncRNA) involved in tumorigenesis and EMT. | [[59](#_ENREF_59)] |
| ***LFNG*** | (8,37,3) | Lunatic fringe (LFNG) encodes a glycosylating enzyme (O‐fucosylpeptide 3‐beta‐N‐acetylglucosaminyltransferase) that regulates NOTCH signaling, hippocampal neural stem cell maintenance and metastasis. | [[60](#_ENREF_60)] |
| ***LIN7A*** | (5,54,191) | Crumbs-complex polarity gene, that regulates tumor progression, hyperproliferation, invasion; its over-expressed in invasive carcinomas by miRNA-501-3p targeted. | [[61](#_ENREF_61)] |
| ***LMBRD2*** | (7,21,112) | Membrane protein LMBRD2 as a potential regulator of β2 adrenoceptor signaling. Identified in neurodevelopmental disorders. | [[62](#_ENREF_62)] |
| ***LMX1B*** | (16,9,87) | The LIM homeodomain transcription factor (Lmx1b) is essential for the development of the isthmic organizer and mesodiencephalic dopaminergic neurons. | [[60](#_ENREF_60)] |
| ***LRRK2*** | (4,22,71) | Leucine-rich repeat kinase 2 (LRRK2) is associated in Parkinson's disease. Autophagy and aging brain. | [[63](#_ENREF_63)] |
| ***MAFF**** | (72,111,1080) | Muscoloaponeurotic fibrosarcoma (MAFF) of basic leucine zipper (bZIP) family transcription factor. Induce IL-1B and TNF in the inflammatory response. | [[64](#_ENREF_64),[65](#_ENREF_65)] |
| ***MBLAC2*** | (7,33,100) | Metallo-β-lactamase (MBL) superfamily enzymes, hydroxy-acylglutathione hydrolase-like (HAGHL) involved in myoclonic epilepsy and dyskinesia. Endonuclease involved in replication-dependent histone pre-mRNA. | [[66](#_ENREF_66)] |
| ***MDGA2*** | (30,97,55) | MAM domain containing glycosylphosphatidylinositol anchor 2 (MDGA2), is a tumor suppressor controls cell proliferation, apoptosis and cell cycle progression. | [[67](#_ENREF_67)] |
| ***MSMO1**** | (7,7,89) | Sterol-C4-methyl oxidase (MSMO1) is involved in the biosynthetic and metabolic processes of sterol and cholesterol. | [[68](#_ENREF_68)] |
| ***MTRNR2L1*** | (23,86,372) | Peptide related with Alzheimer's disease, colorectal liver metastases, neuroprotective and anti-apoptotic functions. | [[69](#_ENREF_69)]  [[70](#_ENREF_70)] |
| ***MTRNR2L2**** | (2,11,105) | Humanins (small anti-apoptotic peptides), its hyper-methylation in association with miR182-5p in breast cancer. | [[71](#_ENREF_71)] |
| ***MTRNR2L10**** | (7,18,164) | circRNAs that were differentially expressed in the maternal blood of prospective FOX cohort preterm fetal growth restriction. Uncharacterized function. | [[72](#_ENREF_72)] |
| ***MTHFD2*** | (67,28, 247) | Mitochondrial methylenetetrahydrofolate dehydrogenase 2 (MTHFD2) involved in tumorigenesis and stem-like properties, which were associated with purine nucleotide deficiency. | [[73](#_ENREF_73)] |
| ***MTRNR2L6*** | (17,75,213) | Humanin-like protein 6 (MTRNR2L6), neuroprotective and antiapoptotic peptide is 6-KSHV miRNAs-infected in Kaposi’s sarcoma-associated herpesvirus (KSHV). Inhibits apoptosis via interaction with the BCL2/BAX proapoptotic protein family. | [[74](#_ENREF_74)]  [[71](#_ENREF_71)] |
| ***MTRNR2L6-2*** | (15,74,265) | Humanin-like protein 6 (MTRNR2L6)c, neuroprotective and antiapoptotic peptide is 6-KSHV miRNAs-infected in Kaposi’s sarcoma-associated herpesvirus (KSHV). Inhibits apoptosis via interaction with the BCL2/BAX proapoptotic protein family. | [[74](#_ENREF_74)]  [[71](#_ENREF_71)] |
|  |  |  |  |
| ***MYH7B*** | (23,74,45) | Sarcomeric myosin gene (Myh7b) encodes an intronic microRNA, in association with miR-499 is involved in skeletal muscle synthesis, contraction force and fatiga. | [[75](#_ENREF_75)] |
| ***MYRIP*** | (60,250,1000) | Myosin- and Rab-interacting protein (MyRIP), which belongs to the protein kinase A (PKA)-anchoring family, is implicated in hormone secretion. | [[76](#_ENREF_76)] |
| ***N4BP2*** | (11,38,19) | Nedd4 binding protein 2 (N4BP2) is a Bcl-3 binding protein, contains a polynucleotide kinase domain (PNK). | [[77](#_ENREF_77)] |
| ***NAA16*** | (8,24,116) | N-terminal acetylation (Nt-Ac) oncogene associated with cancers and their association with patient survival. | [[78](#_ENREF_78)] |
| ***NBEA*** | (17,82,349) | Neurobeachin (NBEA) regulate synapse receptor targeting, synaptic function, cognition, and social behavior. NBEA-KIF21B involved in Autism spectrum disorders. | [[79](#_ENREF_79)] |
| ***NHS*** | (4,13,74) | Nance-Horan Syndrome (NHS)-associated and mammalian development. | [[80](#_ENREF_80)] |
| ***PARD3B*** | (6,24,94) | PARD3B products interact with members of the SMAD (mothers against decapentaplegic homolog) family, mediating multiple signaling pathways and involved in cell growth, apoptosis, morphogenesis, development, and immune responses. Associated with prostate cancer. | [[81](#_ENREF_81)] |
| ***PHF1*** | (4,35,40) | Plant homeodomain finger protein 1 (PHF1) is a essential factor for epigenetic regulation and genome maintenance. Promotes cell proliferation, invasion, and tumorigenesis in a variety of human cancers. | [[82](#_ENREF_82)] |
| ***PKNOX1*** | (14,36,17) | Transcription factor Pknox1 belongs to the three‐amino acid loop extension class, it is essential at multiple stages of embryonic development and apoptosis. | [[83](#_ENREF_83)] |
| ***PLP1**** | (0,7,51) | Myelin proteolipid protein 1 (PLP1) is exclusively localized in the myelin sheath of oligodendrocytes, involved in trafficking and apoptosis. Associated to Pelizaeus-Merzbacher and Multiple Sclerosis desease. | [[84](#_ENREF_84)] |
| ***PPIG*** | (151,772,1877) | Immunopilin, modulates the Calcineurin/NFAT pathway in immune cell activation. Splicing regulation. | [[85](#_ENREF_85)] |
| ***PSD3**** | (3,11,73) | circRNA pleckstrin and Sec7 domain containing 3 (circPSD3) involved in papillary thyroid carcinoma development through regulating miR-637/HEMGN axis and activating PI3K/Akt signaling. Associated with obesity, type 2 diabetes, and cholesterol level. | [[86](#_ENREF_86)]  [[87](#_ENREF_87)] |
| ***RASAL2**** | (39,60,506) | Oncogene, RAS protein activator like 2 (RASAL2) belongs to the RAS GTPase-activating protein family and plays an important role in several cancers, including ovarian cancer, nasopharyngeal carcinoma, malignant astrocytoma, renal cell carcinoma, bladder cancer, colorectal cancer, liver cancer, triple-negative breast cancer, lung adenocarcinoma, and pancreatic ductal adenocarcinoma. Authophagy, angiogenesis, and adipogenesis. | [[88](#_ENREF_88)] |
| ***RASL11A*** | (43,96,54) | Protein of the Ras super-family, it is is a chromatin‐associated modulator of pre‐ribosomal RNA (pre‐rRNA) synthesis. | [[89](#_ENREF_89)] |
| ***RELT*** | (2,17,56) | Human tumor necrosis factor receptor (TNFR) family member expressed in lymphoid tissues (RELT) induces apoptosis by SPAK kinase to mediate p38 and JNK activation. | [[90](#_ENREF_90)] |
| ***RFLNB-2*** | (21,48,25) | Gene expressed in placental with severe preeclampsia. | [[91](#_ENREF_91)] |
| ***RFX1-2*** | (5,37,84) | Epigenetic regulation of IL-6/STAT3 pathway. | [[92](#_ENREF_92)] |
| ***RGPD3*** | (2,14,50) | RanBP2-type ZF family or RNA transport is over-expressed in cancers. | [[93](#_ENREF_93)] |
| ***RMI2*** | (12,75,86) | Transcription regulator associated with cervical squamous cell carcinoma (CESC) methylation and expression. Tumor suppressor, growth and metastasis. | [[94](#_ENREF_94)] |
| ***RORB*** | (5,16,75) | Retinoic acid-related orphan receptor B (RORB) regulates insulin secretion in pancreatic β cells, diabetic/hyperglycemic, insulin secretion, maturation of photoreceptors, and epilepsy. | [[95](#_ENREF_95)]  [[96](#_ENREF_96)] |
| ***SAMD4A*** | (52,61,443) | SMAUG1 protein-like promote Hepatitis B virus RNA degradation, mTORC1 modulation. | [[97](#_ENREF_97)] |
| ***SEC24A*** | (24,22,191) | COPII coat complex component (SEC24A) is a cytosolic protein, belonging to the COPII secretory machinery of endoplasmic reticulum (ER) to the Golgi apparatus. | [[98](#_ENREF_98)] |
| ***SGK1*** | (138,380,201) | The serum and glucocorticoid inducible kinase-1 (SGK1) modulates hyperglucemia and authophagy-dependent apoptosis. | [[99](#_ENREF_99)] |
| ***SGIP1**** | (6,6,77) | The SH3-domain GRB2-like (endophilin) interacting protein 1 (SGIP1) functions as an endocytic protein involved in clathrin-mediated endocytosis | [[100](#_ENREF_100)] |
| ***SCLT1*** | (1,10,44) | Sodium channel and clathrin linker 1 (SCLT1) ciliary protein related with Bardet–biedl syndrome and Ciliopathy. | [[101](#_ENREF_101)] |
| ***SH3KBP1*** | (14,62,187) | SH3-domain kinase binding protein 1 (Sh3kbp1) involved in carcinomas chemotherapy resistance. | [[102](#_ENREF_102)] |
| ***SLF1*** | (15,62,214) | SLF1 and SLF2 (Smc5/6 localization factors 1 and 2), link with RAD18 of the SMC5/6 complex to RNF8/RNF168-generated ubiquitylations at damaged DNA. | [[103](#_ENREF_103)] |
| ***SMAD6*** | (36,115,38) | Member of the transforming growth factor β/bone morphogenetic protein (TGFβ/BMP) superfamily, associated with craniosynostosis. | [[104](#_ENREF_104)] |
| ***SMCR8-2*** | (43,14,150) | Smith-Magenis syndrome chromosome region candidate 8 protein is associated with authophagy and neurodegenerative diseases; controls AKT/mTORC1. | [[105](#_ENREF_105)] |
| ***STK36*** | (1,24,35) | Serine/threonine protein kinase involved in Hedgehog signaling, embryogenesis, tissue regeneration and carcinogenesis. | [[106](#_ENREF_106)] |
| ***SYNPO*** | (7,43,89) | SYNPO (synaptopodin) protein is implicated in structural and functional of dendrites and actic cytoskeleton. | [[107](#_ENREF_107)] |
| ***TBC1D2**** | (9,13,110) | TBC1D2b is a Rab22 GTPase-activating protein that forms a complex with ZEB1/NuRD, potent suppressor of NSCLC invasion and metastasis by EMT, suppressing E-cadherin internalization. Autophagy. | [[108](#_ENREF_108)]  [[109](#_ENREF_109)] |
| ***TBC1D25*** | (2,10,57) | TBC1D25 showed inhibitory role in the maturation step of autophagosomes depending of binding to LC3. Overexpression inhibits phosphorylation of TAK1, JNK and p38. Related with autophagy and pathological cardiac remodeling. | [[110](#_ENREF_110)]  [[111](#_ENREF_111)] |
| ***TECR*** | (5,39,35) | The trans-2,3-enoyl-CoA reductase (TECR) a synaptic glycoprotein associated with non-syndromic mental retardation. | [112] |
| ***TMPRSS6*** | (3,30,36) | Matriptase-2 is a type II transmembrane serine protease involved in iron homoeostasis, cleaves haemojuvelin (HJV) and regulates the BMP/SMAD signalling pathway leading to production of hepcidin. Low expressed in hepatocellular carcinoma cell lines. | [[113](#_ENREF_113)] |
| ***TRIB1*** | (47,225,102) | Tribbles pseudokinase 1 (Trib1) is a central regulator of antiviral T cell immunity. | [[114](#_ENREF_114)] |
| ***TRPM7*** | (103,271,1276) | Channel-Kinase TRPM7 associated with immunity and immune cell signalling. In lymphocyte proliferation, differentiation, activation and survival. Contributes to malignant neoplasia. | [[115](#_ENREF_115)]  [[116](#_ENREF_116)] |
| ***TTBK2*** | (3,18,60) | Tau-tubulin kinase-2 (TTBK2) is linked to spinocerebellar ataxia type 11, crucial for ciliogenesis. Enhanced expression of circ-TTBK2 promoted cell proliferation, migration, and invasion, while inhibited apoptosis. | [[117](#_ENREF_117)]  [[118](#_ENREF_118)] |
| ***TXNIP*** | (36,20,170) | Thioredoxin-interacting protein (TXNIP), a major cellular thiol-reducing and antioxidant system. Implicated in neurodegenerative diseases and glucose homeostasis. | [[119](#_ENREF_119)] |
| ***VPS13A*** | (6,40,121) | VPS13 protein involved in lipid transport, membrane traffic at Golgi–endosome interfaces, cytoskeletal organization, mitochondrial health, and autophagy. Responsible for neurodevelopmental and neurodegenerative disorders including chorea acanthocytosis (VPS13A). | [[120](#_ENREF_120)] |
| ***VPS13C*** | (18,46,240) | VPS13 protein involved in lipid transport, responsible for neurodevelopmental and neurodegenerative disorders including Parkinson's disease (VPS13C). | [[120](#_ENREF_120)] |
| ***WDR37*** | (12,3,49) | Member of the WD40 repeats (WDRs) associated with ocular colobomas, cerebellar malformations, developmental delay, intellectual disability, facial dysmorphism, and epilepsy. | [[121](#_ENREF_121)] |
| ***YOD1**** | (6,15,119) | YOD1 deubiquitinates ITCH, an E3 ligase of LATS, and a subsequent increase he YAP/TAZ level, is a regulator of the Hippo pathway and associated to liver cancer. Antagonizes TRAF6/p62-dependent IL-1 signaling to NF-κB. | [[122](#_ENREF_122)]  [[123](#_ENREF_123)] |
| ***ZFY*** | (7,34,136) | Triggers germ cell apoptosis during stage IV of the spermatogenic cycle. Down-regulated in several cancer types. | [[124](#_ENREF_124)]  [[125](#_ENREF_125)] |
| ***ZNF202**** | (3,2,36) | The zinc finger protein 202 (ZNF202) transcription repressor of a number of genes involved in lipid metabolism. | [[126](#_ENREF_126)] |
| ***ZNF236*** | (3,16,60) | Kruppel-like zinc-finger gene (ZNF) its glucose-regulated expression in human mesangial cells and neuroradiological abnormalities. | [[127](#_ENREF_127)] |
| ***ZNF569*** | (10,10,85) | The zinc finger protein 569 (ZNF569) inhibites the proliferation, migration and invasion of head and neck squamous cells carcinoma. | [[128](#_ENREF_128),[129](#_ENREF_129)] |
| ***ZSWIM3*** | (27,18,186) | The zinc finger-chelate domain of SWIM, functions in DNA-binding and protein-binding interactions. Its associated with inflammation TRAF2-mediated NF-kB pathway. | [[130](#_ENREF_130)] |

| **DEGs** | **mRNA reads**  **(T0, T15min, T4h)** | **Cellular Process Target** | **References** |
| --- | --- | --- | --- |
| ***BTG2**** | (27,6,7) | B-cell translocation gene 2 (BTG2) PI3K/Akt1, NFκB pathways. Tumor suppressor involved in cell division, DNA repair, transcriptional regulation and messenger RNA stability. | [[131](#_ENREF_131)] |
| ***CD244**** | (30,21,21) | Transmembrane receptor in the signaling lymphocyte activation molecule (SLAM) family, involved in tumor-mediated immune cell regulation and proliferation ability of leukemia initiating cells through SHP-2/p27 kip1 signaling. | [[132](#_ENREF_132)]  [[133](#_ENREF_133)] |
| ***CDC6**** | (73,56,51) | Cell division cycle 6 (Cdc6) is an essential regulator of pre-replication complex assembly and DNA replication. Proliferation of keratinocytes and osteosarcoma. | [[134](#_ENREF_134)] |
| ***CHADL**** | (24,10,17) | Chondroadherin-like (CHADL) regulator of collagen fibrillogenesis, and modulates chondrocyte differentiation. | [[135](#_ENREF_135)]. |
| ***CHRM4**** | (14,8,3) | Cholinergic receptor muscarinic 4 (CHRM4) involved in anemias of myelodysplastic syndrome (MDS), aging, and hemolysis. | [[136](#_ENREF_136)] |
| ***COL6A1**** | (22,0,0) | Collagen type VI α1 chain (COL6A1) decreased in migration and invasion, causing suppressed EMT. | [[137](#_ENREF_137)] |
| ***DGCR8**** | (500,438,372) | DiGeorge syndrome critical region 8 (DGCR8) is a critical component of the miRNA biogenesis. It plays an important role in maintaining heterochromatin organization, attenuating aging and involved in malignant of glioma cells. | [[138](#_ENREF_138)]  [[139](#_ENREF_139)] |
| ***HFM1**** | (594,333,153) | Helicase for meiosis 1 (HFM1) participates in Golgi-associated spindle assembly and division in mouse oocyte meiosis. | [[140](#_ENREF_140)] |
| ***HIST1H4K**** | (19,5,4) | Histone cluster 1 H4 family member k (HIST1H4K), plays essential roles in epigenetic regulation, cancer and pathogenesis in patients with systemic lupus erythematosus. | [[141](#_ENREF_141),[142](#_ENREF_142)] |
| ***LINGO1**** | (834,753,308) | Leucine-rich repeat and Ig domain containing 1 gene (LINGO1), is one of four leucine rich repeat and immunoglobin-like (LRRIG) domain-containing proteins predominantly expressed in the central nervous system, is involved in essential Tremor and Parkinson’s diseases. | [[143](#_ENREF_143)] |
| ***OXLD1**** | (14,0,1) | Oxidoreductase-like domain-containing protein 1 (OXLD1) over-expressed in several cancer types. | https://www.proteinatlas.org |
| ***PPIAL4A**** | (26,14,15) | Peptidyl-prolyl cis-trans isomerase A-like 4A (PPIAL4A). Overexpression is related with aggressivity, metastatic or chemotherapy resistant in tumours. | [[144](#_ENREF_144)] |
| ***SYNC**** | (52,47,23) | Syncoilin (SYNC) is an intermediate filament protein highly expressed in skeletal and cardiac muscle. Its is associated with myopathy, myofibrillar, and neuromuscular disease. | https://www.proteinatlas.org |
| ***UNC45B**** | (37,25,8) | Molecular chaperone that mediates the folding of thick-filament myosin during sarcomere formation and myofibrillogenesis, related with cytoskeletal and cardiac myopathies. | [[145](#_ENREF_145)] |

**TFs DOWN-REGULATED by the CDPs exposure in HeLa cells.**

**Additional References:**

1. Artinian N, Cloninger C, Holmes B, Benavides-Serrato A, Bashir T, Gera J (2015) Phosphorylation of the Hippo Pathway Component AMOTL2 by the mTORC2 Kinase Promotes YAP Signaling, Resulting in Enhanced Glioblastoma Growth and Invasiveness. J Biol Chem 290 (32):19387-19401. doi:10.1074/jbc.M115.656587

2. Karedath T, Ahmed I, Al Ameri W, Al-Dasim FM, Andrews SS, Samuel S, Al-Azwani IK, Mohamoud YA, Rafii A, Malek JA (2019) Silencing of ANKRD12 circRNA induces molecular and functional changes associated with invasive phenotypes. BMC cancer 19 (1):565. doi:10.1186/s12885-019-5723-0

3. Xu Y, Ye S, Zhang N, Zheng S, Liu H, Zhou K, Wang L, Cao Y, Sun P, Wang T (2020) The FTO/miR-181b-3p/ARL5B signaling pathway regulates cell migration and invasion in breast cancer. Cancer communications (London, England) 40 (10):484-500. doi:10.1002/cac2.12075

4. Houghton FJ, Bellingham SA, Hill AF, Bourges D, Ang DK, Gemetzis T, Gasnereau I, Gleeson PA (2012) Arl5b is a Golgi-localised small G protein involved in the regulation of retrograde transport. Experimental cell research 318 (5):464-477. doi:10.1016/j.yexcr.2011.12.023

5. Hu Y, Zhao Y, Shi C, Ren P, Wei B, Guo Y, Ma J (2019) A circular RNA from APC inhibits the proliferation of diffuse large B-cell lymphoma by inactivating Wnt/β-catenin signaling via interacting with TET1 and miR-888. Aging 11 (19):8068-8084. doi:10.18632/aging.102122

6. Bogutz AB, Oh-McGinnis R, Jacob KJ, Ho-Lau R, Gu T, Gertsenstein M, Nagy A, Lefebvre L (2018) Transcription factor ASCL2 is required for development of the glycogen trophoblast cell lineage. PLoS Genet 14 (8):e1007587-e1007587. doi:10.1371/journal.pgen.1007587

7. Schroeder N, Wuelling M, Hoffmann D, Brand-Saberi B, Vortkamp A (2019) Atoh8 acts as a regulator of chondrocyte proliferation and differentiation in endochondral bones. PLoS One 14 (8):e0218230-e0218230. doi:10.1371/journal.pone.0218230

8. Nandakumar P, Mansouri A, Das S (2017) The Role of ATRX in Glioma Biology. Frontiers in oncology 7:236. doi:10.3389/fonc.2017.00236

9. Siddiqui WA, Ahad A, Ahsan H (2015) The mystery of BCL2 family: Bcl-2 proteins and apoptosis: an update. Archives of toxicology 89 (3):289-317. doi:10.1007/s00204-014-1448-7

10. Walker SR, Nelson EA, Frank DA (2007) STAT5 represses BCL6 expression by binding to a regulatory region frequently mutated in lymphomas. Oncogene 26 (2):224-233. doi:10.1038/sj.onc.1209775

11. Cardenas MG, Oswald E, Yu W, Xue F, MacKerell AD, Jr., Melnick AM The Expanding Role of the BCL6 Oncoprotein as a Cancer Therapeutic Target. (1078-0432 (Print))

12. van der Spek A, Warner SC, Broer L, Nelson CP, Vojinovic D, Ahmad S, Arp PP, Brouwer RWW, Denniff M, van den Hout M, van Rooij JGJ, Kraaij R, van IWFJ, Samani NJ, Ikram MA, Uitterlinden AG, Codd V, Amin N, van Duijn CM (2020) Exome Sequencing Analysis Identifies Rare Variants in ATM and RPL8 That Are Associated With Shorter Telomere Length. Frontiers in genetics 11:337. doi:10.3389/fgene.2020.00337

13. Thomas J, Leufflen L, Chesnais V, Diry S, Demange J, Depardieu C, Bani MA, Marchal F, Charra-Brunaud C, Merlin JL, Leroux A, Sastre-Garau X, Harlé A (2020) Identification of Specific Tumor Markers in Vulvar Carcinoma Through Extensive Human Papillomavirus DNA Characterization Using Next Generation Sequencing Method. Journal of lower genital tract disease 24 (1):53-60. doi:10.1097/lgt.0000000000000498

14. Tanouchi A, Taniuchi K, Furihata M, Naganuma S, Dabanaka K, Kimura M, Watanabe R, Kohsaki T, Shimizu T, Saito M, Hanazaki K, Saibara T (2016) CCDC88A, a prognostic factor for human pancreatic cancers, promotes the motility and invasiveness of pancreatic cancer cells. Journal of experimental & clinical cancer research : CR 35 (1):190. doi:10.1186/s13046-016-0466-0

15. Leguit RJ, Raymakers RAP, Hebeda KM, Goldschmeding R (2021) CCN2 (Cellular Communication Network factor 2) in the bone marrow microenvironment, normal and malignant hematopoiesis. J Cell Commun Signal 15 (1):25-56. doi:10.1007/s12079-020-00602-2

16. Kaasbøll OJ, Gadicherla AK, Wang J-H, Monsen VT, Hagelin EMV, Dong M-Q, Attramadal H (2018) Connective tissue growth factor (CCN2) is a matricellular preproprotein controlled by proteolytic activation. J Biol Chem 293 (46):17953-17970. doi:10.1074/jbc.RA118.004559

17. Liang Y, Ahmed M, Guo H, Soares F, Hua JT, Gao S, Lu C, Poon C, Han W, Langstein J, Ekram MB, Li B, Davicioni E, Takhar M, Erho N, Karnes RJ, Chadwick D, van der Kwast T, Boutros PC, Arrowsmith CH, Feng FY, Joshua AM, Zoubeidi A, Cai C, He HH (2017) LSD1-Mediated Epigenetic Reprogramming Drives CENPE Expression and Prostate Cancer Progression. Cancer research 77 (20):5479-5490. doi:10.1158/0008-5472.can-17-0496

18. Qin Y, Zhou Y, Shen Z, Xu B, Chen M, Li Y, Chen M, Behrens A, Zhou J, Qi X, Meng W, Wang Y, Gao F (2019) WDR62 is involved in spindle assembly by interacting with CEP170 in spermatogenesis. Development (Cambridge, England) 146 (20). doi:10.1242/dev.174128

19. Chen M-S, Wang S-F, Hsu C-Y, Yin P-H, Yeh T-S, Lee H-C, Tseng L-M (2017) CHAC1 degradation of glutathione enhances cystine-starvation-induced necroptosis and ferroptosis in human triple negative breast cancer cells via the GCN2-eIF2α-ATF4 pathway. Oncotarget 8 (70):114588-114602. doi:10.18632/oncotarget.23055

20. Mungrue IN, Pagnon J, Kohannim O, Gargalovic PS, Lusis AJ (2009) CHAC1/MGC4504 is a novel proapoptotic component of the unfolded protein response, downstream of the ATF4-ATF3-CHOP cascade. Journal of immunology (Baltimore, Md : 1950) 182 (1):466-476. doi:10.4049/jimmunol.182.1.466

21. Yuan H, Du S, Deng Y, Xu X, Zhang Q, Wang M, Wang P, Su Y, Liang X, Sun Y, An Z (2019) Effects of microRNA-208a on inflammation and oxidative stress in ketamine-induced cardiotoxicity through Notch/NF-κB signal pathways by CHD9. Bioscience reports 39 (5). doi:10.1042/bsr20182381

22. Eisenreich A, Zakrzewicz A, Huber K, Thierbach H, Pepke W, Goldin-Lang P, Schultheiss HP, Pries A, Rauch U (2013) Regulation of pro-angiogenic tissue factor expression in hypoxia-induced human lung cancer cells. Oncology reports 30 (1):462-470. doi:10.3892/or.2013.2413

23. Xue H, Zhang J, Guo X, Wang J, Li J, Gao X, Guo X, Li T, Xu S, Zhang P, Liu Q, Li G (2016) CREBRF is a potent tumor suppressor of glioblastoma by blocking hypoxia-induced autophagy via the CREB3/ATG5 pathway. International journal of oncology 49 (2):519-528. doi:10.3892/ijo.2016.3576

24. Ma X, Zhang H, Yuan L, Jing H, Thacker P, Li D (2011) CREBL2, interacting with CREB, induces adipogenesis in 3T3-L1 adipocytes. The Biochemical journal 439 (1):27-38. doi:10.1042/bj20101475

25. Steckelberg AL, Altmueller J, Dieterich C, Gehring NH (2015) CWC22-dependent pre-mRNA splicing and eIF4A3 binding enables global deposition of exon junction complexes. Nucleic acids research 43 (9):4687-4700. doi:10.1093/nar/gkv320

26. Nawabi H, Belin S, Cartoni R, Williams PR, Wang C, Latremolière A, Wang X, Zhu J, Taub DG, Fu X, Yu B, Gu X, Woolf CJ, Liu JS, Gabel CV, Steen JA, He Z (2015) Doublecortin-Like Kinases Promote Neuronal Survival and Induce Growth Cone Reformation via Distinct Mechanisms. Neuron 88 (4):704-719. doi:10.1016/j.neuron.2015.10.005

27. Block I, Müller C, Sdogati D, Pedersen H, List M, Jaskot AM, Syse SD, Lund Hansen P, Schmidt S, Christiansen H, Casella C, Bering Olsen S, Blomstrøm MM, Riedel A, Thomassen M, Kruse TA, Karlskov Hansen SW, Kioschis P, Mollenhauer J (2019) CFP suppresses breast cancer cell growth by TES-mediated upregulation of the transcription factor DDIT3. Oncogene 38 (23):4560-4573. doi:10.1038/s41388-019-0739-0

28. He L, Shi X, Liu Z, Ren X, Zhang C, Yang Z, Li Z (2019) Roles Of EAAT1, DHFR, And Fetuin-A In The Pathogenesis, Progression And Prognosis Of Chondrosarcoma. OncoTargets and therapy 12:8411-8420. doi:10.2147/ott.s222426

29. Cáceres A, Perdiguero B, Gómez CE, Cepeda MV, Caelles C, Sorzano CO, Esteban M (2013) Involvement of the cellular phosphatase DUSP1 in vaccinia virus infection. PLoS Pathog 9 (11):e1003719-e1003719. doi:10.1371/journal.ppat.1003719

30. Ding T, Zhou Y, Long R, Chen C, Zhao J, Cui P, Guo M, Liang G, Xu L (2019) DUSP8 phosphatase: structure, functions, expression regulation and the role in human diseases. Cell & bioscience 9:70. doi:10.1186/s13578-019-0329-4

31. Schriever SC, Kabra DG, Pfuhlmann K, Baumann P, Baumgart EV, Nagler J, Seebacher F, Harrison L, Irmler M, Kullmann S, Corrêa-da-Silva F, Giesert F, Jain R, Schug H, Castel J, Martinez S, Wu M, Häring HU, de Angelis MH, Beckers J, Müller TD, Stemmer K, Wurst W, Rozman J, Nogueiras R, De Angelis M, Molkentin JD, Krahmer N, Yi CX, Schmidt MV, Luquet S, Heni M, Tschöp MH, Pfluger PT (2020) Type 2 diabetes risk gene Dusp8 regulates hypothalamic Jnk signaling and insulin sensitivity. The Journal of clinical investigation 130 (11):6093-6108. doi:10.1172/jci136363

32. Buchsbaum IY, Kielkowski P, Giorgio G, O'Neill AC, Di Giaimo R, Kyrousi C, Khattak S, Sieber SA, Robertson SP, Cappello S (2020) ECE2 regulates neurogenesis and neuronal migration during human cortical development. EMBO Rep 21 (5):e48204-e48204. doi:10.15252/embr.201948204

33. Pusapati GV, Hughes CE, Dorn KV, Zhang D, Sugianto P, Aravind L, Rohatgi R (2014) EFCAB7 and IQCE regulate hedgehog signaling by tethering the EVC-EVC2 complex to the base of primary cilia. Developmental cell 28 (5):483-496. doi:10.1016/j.devcel.2014.01.021

34. Morita K, Okamura T, Inoue M, Komai T, Teruya S, Iwasaki Y, Sumitomo S, Shoda H, Yamamoto K, Fujio K (2016) Egr2 and Egr3 in regulatory T cells cooperatively control systemic autoimmunity through Ltbp3-mediated TGF-β3 production. Proceedings of the National Academy of Sciences of the United States of America 113 (50):E8131-e8140. doi:10.1073/pnas.1611286114

35. Taefehshokr S, Key YA, Khakpour M, Dadebighlu P, Oveisi A (2017) Early growth response 2 and Egr3 are unique regulators in immune system. Cent Eur J Immunol 42 (2):205-209. doi:10.5114/ceji.2017.69363

36. Chen JY, Tan X, Wang ZH, Liu YZ, Zhou JF, Rong XZ, Lu L, Li Y (2018) The ribosome biogenesis protein Esf1 is essential for pharyngeal cartilage formation in zebrafish. The FEBS journal 285 (18):3464-3484. doi:10.1111/febs.14622

37. Yao HL, Liu M, Wang WJ, Wang XL, Song J, Song QQ, Han J (2019) Construction of miRNA-target networks using microRNA profiles of CVB3-infected HeLa cells. Scientific reports 9 (1):17876. doi:10.1038/s41598-019-54188-w

38. Kwon M, Kim JH, Rybak Y, Luna A, Choi CH, Chung JY, Hewitt SM, Adem A, Tubridy E, Lin J, Libutti SK (2016) Reduced expression of FILIP1L, a novel WNT pathway inhibitor, is associated with poor survival, progression and chemoresistance in ovarian cancer. Oncotarget 7 (47):77052-77070. doi:10.18632/oncotarget.12784

39. Cuiffo BG, Campagne A, Bell GW, Lembo A, Orso F, Lien EC, Bhasin MK, Raimo M, Hanson SE, Marusyk A, El-Ashry D, Hematti P, Polyak K, Mechta-Grigoriou F, Mariani O, Volinia S, Vincent-Salomon A, Taverna D, Karnoub AE (2014) MSC-regulated microRNAs converge on the transcription factor FOXP2 and promote breast cancer metastasis. Cell stem cell 15 (6):762-774. doi:10.1016/j.stem.2014.10.001

40. van der Wal T, Lambooij J-P, van Amerongen R (2020) TMEM98 is a negative regulator of FRAT mediated Wnt/ß-catenin signalling. PLoS One 15 (1):e0227435-e0227435. doi:10.1371/journal.pone.0227435

41. Du X, Li Q, Yang L, Liu L, Cao Q, Li Q (2020) SMAD4 activates Wnt signaling pathway to inhibit granulosa cell apoptosis. Cell Death Dis 11 (5):373-373. doi:10.1038/s41419-020-2578-x

42. Cui D, Sajan P, Shi J, Shen Y, Wang K, Deng X, Zhou L, Hu P, Gao L (2017) MiR-148a increases glioma cell migration and invasion by downregulating GADD45A in human gliomas with IDH1 R132H mutations. Oncotarget 8 (15):25345-25361. doi:10.18632/oncotarget.15867

43. Dong C, Wang X, Li N, Zhang K, Wang X, Zhang H, Wang H, Wang B, An M, Ma B (2019) microRNA-mediated GAS1 downregulation promotes the proliferation of synovial fibroblasts by PI3K-Akt signaling in osteoarthritis. Exp Ther Med 18 (6):4273-4286. doi:10.3892/etm.2019.8101

44. Witkos TM, Chan WL, Joensuu M, Rhiel M, Pallister E, Thomas-Oates JA-O, Mould AP, Mironov AA, Biot CA-O, Guerardel YA-O, Morelle W, Ungar DA-O, Wieland FT, Jokitalo E, Tassabehji M, Kornak U, Lowe M GORAB scaffolds COPI at the trans-Golgi for efficient enzyme recycling and correct protein glycosylation. (2041-1723 (Electronic))

45. Liu Y, Chen X, Choi YJ, Yang N, Song Z, Snedecor ER, Liang W, Leung EL, Zhang L, Qin C, Chen J (2020) GORAB promotes embryonic lung maturation through antagonizing AKT phosphorylation, versican expression, and mesenchymal cell migration. FASEB journal : official publication of the Federation of American Societies for Experimental Biology 34 (4):4918-4933. doi:10.1096/fj.201902075R

46. Wang T, Nakagawa S, Miyake T, Setsu G, Kunisue S, Goto K, Hirasawa A, Okamura H, Yamaguchi Y, Doi M (2020) Identification and functional characterisation of N-linked glycosylation of the orphan G protein-coupled receptor Gpr176. Scientific reports 10 (1):4429-4429. doi:10.1038/s41598-020-61370-y

47. Liu X-J, Yang B, Huang S-N, Wu C-C, Li X-J, Cheng S, Jiang X, Hu F, Ming Y-Z, Nevels M, Britt WJ, Rayner S, Tang Q, Zeng W-B, Zhao F, Luo M-H (2017) Human cytomegalovirus IE1 downregulates Hes1 in neural progenitor cells as a potential E3 ubiquitin ligase. PLoS Pathog 13 (7):e1006542-e1006542. doi:10.1371/journal.ppat.1006542

48. Dhont L, Mascaux C, Belayew A (2016) The helicase-like transcription factor (HLTF) in cancer: loss of function or oncomorphic conversion of a tumor suppressor? Cellular and molecular life sciences : CMLS 73 (1):129-147. doi:10.1007/s00018-015-2060-6

49. Zhang S, Mo Q, Wang X (2019) Oncological role of HMGA2 (Review). International journal of oncology 55 (4):775-788. doi:10.3892/ijo.2019.4856

50. Gao X, Dai M, Li Q, Wang Z, Lu Y, Song Z (2017) HMGA2 regulates lung cancer proliferation and metastasis. Thoracic cancer 8 (5):501-510. doi:10.1111/1759-7714.12476

51. Mammen AL, Chung T, Christopher-Stine L, Rosen P, Rosen A, Doering KR, Casciola-Rosen LA (2011) Autoantibodies against 3-hydroxy-3-methylglutaryl-coenzyme A reductase in patients with statin-associated autoimmune myopathy. Arthritis and rheumatism 63 (3):713-721. doi:10.1002/art.30156

52. Mori K, Nakamura H, Kurooka H, Miyachi H, Tamada K, Sugai M, Takumi T, Yokota Y (2018) Id2 Determines Intestinal Identity through Repression of the Foregut Transcription Factor Irx5. Molecular and cellular biology 38 (9):e00250-00217. doi:10.1128/MCB.00250-17

53. Fang C-Y, Chen M-C, Chang T-H, Wu C-C, Chang J-P, Huang H-D, Ho W-C, Wang Y-Z, Pan K-L, Lin Y-S, Huang Y-K, Chen C-J, Lee W-C (2018) Idi1 and Hmgcs2 Are Affected by Stretch in HL-1 Atrial Myocytes. Int J Mol Sci 19 (12):4094. doi:10.3390/ijms19124094

54. Ruan W, Zhu S, Wang H, Xu F, Deng H, Ma Y, Lai M (2010) IGFBP-rP1, a potential molecule associated with colon cancer differentiation. Mol Cancer 9:281-281. doi:10.1186/1476-4598-9-281

55. Werner A, Iwasaki S, McGourty CA, Medina-Ruiz S, Teerikorpi N, Fedrigo I, Ingolia NT, Rape M (2015) Cell-fate determination by ubiquitin-dependent regulation of translation. Nature 525 (7570):523-527. doi:10.1038/nature14978

56. Li YR, Peng RR, Gao WY, Liu P, Chen LJ, Zhang XL, Zhang NN, Wang Y, Du L, Zhu FY, Wang LL, Li CR, Zeng WT, Li JM, Hu F, Zhang D, Yang ZX (2019) The ubiquitin ligase KBTBD8 regulates PKM1 levels via Erk1/2 and Aurora A to ensure oocyte quality. Aging 11 (4):1110-1128. doi:10.18632/aging.101802

57. Hofvander J, Tayebwa J, Nilsson J, Magnusson L, Brosjö O, Larsson O, von Steyern FV, Domanski HA, Mandahl N, Mertens F (2015) RNA sequencing of sarcomas with simple karyotypes: identification and enrichment of fusion transcripts. Laboratory investigation; a journal of technical methods and pathology 95 (6):603-609. doi:10.1038/labinvest.2015.50

58. Janisch KM, McNeely KC, Dardick JM, Lim SH, Dwyer ND (2018) Kinesin-6 KIF20B is required for efficient cytokinetic furrowing and timely abscission in human cells. Molecular biology of the cell 29 (2):166-179. doi:10.1091/mbc.E17-08-0495

59. Liu C, Li X, Hao Y, Wang F, Cheng Z, Geng H, Geng D (2020) STAT1-induced upregulation of lncRNA KTN1-AS1 predicts poor prognosis and facilitates non-small cell lung cancer progression via miR-23b/DEPDC1 axis. Aging 12 (9):8680-8701. doi:10.18632/aging.103191

60. Del Castillo Velasco-Herrera M, van der Weyden L, Nsengimana J, Speak AO, Sjöberg MK, Bishop DT, Jönsson G, Newton-Bishop J, Adams DJ (2018) Comparative genomics reveals that loss of lunatic fringe (LFNG) promotes melanoma metastasis. Mol Oncol 12 (2):239-255. doi:10.1002/1878-0261.12161

61. Luo C, Yin D, Zhan H, Borjigin U, Li C, Zhou Z, Hu Z, Wang P, Sun Q, Fan J, Zhou J, Wang X, Zhou S, Huang X (2018) microRNA-501-3p suppresses metastasis and progression of hepatocellular carcinoma through targeting LIN7A. Cell death & disease 9 (5):535. doi:10.1038/s41419-018-0577-y

62. Paek J, Kalocsay M, Staus DP, Wingler L, Pascolutti R, Paulo JA, Gygi SP, Kruse AC (2017) Multidimensional Tracking of GPCR Signaling via Peroxidase-Catalyzed Proximity Labeling. Cell 169 (2):338-349.e311. doi:10.1016/j.cell.2017.03.028

63. Albanese F, Novello S, Morari M (2019) Autophagy and LRRK2 in the Aging Brain. Front Neurosci 13:1352-1352. doi:10.3389/fnins.2019.01352

64. Massrieh W, Derjuga A, Doualla-Bell F, Ku C-Y, Sanborn BM, Blank V (2006) Regulation of the MAFF Transcription Factor by Proinflammatory Cytokines in Myometrial Cells1. Biology of Reproduction 74 (4):699-705. doi:10.1095/biolreprod.105.045450

65. Eleveld TF, Schild L, Koster J, Zwijnenburg DA, Alles LK, Ebus ME, Volckmann R, Tijtgat GA, van Sluis P, Versteeg R, Molenaar JJ (2018) RAS-MAPK Pathway-Driven Tumor Progression Is Associated with Loss of CIC and Other Genomic Aberrations in Neuroblastoma. Cancer research 78 (21):6297-6307. doi:10.1158/0008-5472.can-18-1045

66. Malgapo MIP, Safadi JM, Linder ME (2020) Metallo-β-Lactamase Domain-Containing Protein 2 (MBLAC2) is S-palmitoylated and exhibits acyl-CoA hydrolase activity. The Journal of biological chemistry. doi:10.1074/jbc.RA120.015701

67. Wang K, Liang Q, Li X, Tsoi H, Zhang J, Wang H, Go MYY, Chiu PWY, Ng EKW, Sung JJY, Yu J (2016) MDGA2 is a novel tumour suppressor cooperating with DMAP1 in gastric cancer and is associated with disease outcome. Gut 65 (10):1619-1631. doi:10.1136/gutjnl-2015-309276

68. Xu P, Wu M, Chen H, Xu J, Wu M, Li M, Qian F, Xu J (2016) Bioinformatics analysis of hepatitis C virus genotype 2a-induced human hepatocellular carcinoma in Huh7 cells. OncoTargets and therapy 9:191-202. doi:10.2147/OTT.S91748

69. Bik-Multanowski M, Pietrzyk JJ, Midro A (2015) MTRNR2L12: A Candidate Blood Marker of Early Alzheimer's Disease-Like Dementia in Adults with Down Syndrome. Journal of Alzheimer's disease : JAD 46 (1):145-150. doi:10.3233/jad-143030

70. Hof J, Visser L, Höppener DJ, Nierop PMH, Terpstra MM, Gouw ASH, Grünhagen DJ, Verhoef C, Sijmons RH, de Jong KP, Kok K (2020) B Cells as Prognostic Biomarker After Surgery for Colorectal Liver Metastases. Frontiers in oncology 10:249. doi:10.3389/fonc.2020.00249

71. Duforestel M, Nadaradjane A, Bougras-Cartron G, Briand J, Olivier C, Frenel JS, Vallette FM, Lelièvre SA, Cartron PF (2019) Glyphosate Primes Mammary Cells for Tumorigenesis by Reprogramming the Epigenome in a TET3-Dependent Manner. Frontiers in genetics 10:885. doi:10.3389/fgene.2019.00885

72. Hannan NJ, Stock O, Spencer R, Whitehead C, David AL, Groom K, Petersen S, Henry A, Said JM, Seeho S, Kane SC, Gordon L, Beard S, Chindera K, Karegodar S, Hiscock R, Pritchard N, Kaitu'u-Lino TuJ, Walker SP, Tong S (2020) Circulating mRNAs are differentially expressed in pregnancies with severe placental insufficiency and at high risk of stillbirth. BMC Med 18 (1):145-145. doi:10.1186/s12916-020-01605-x

73. Nishimura T, Nakata A, Chen X, Nishi K, Meguro-Horike M, Sasaki S, Kita K, Horike S-I, Saitoh K, Kato K, Igarashi K, Murayama T, Kohno S, Takahashi C, Mukaida N, Yano S, Soga T, Tojo A, Gotoh N (2019) Cancer stem-like properties and gefitinib resistance are dependent on purine synthetic metabolism mediated by the mitochondrial enzyme MTHFD2. Oncogene 38 (14):2464-2481. doi:10.1038/s41388-018-0589-1

74. Quan L, Qiu T, Liang J, Li M, Zhang Y, Tao K (2015) Identification of Target Genes Regulated by KSHV miRNAs in KSHV-Infected Lymphoma Cells. Pathology oncology research : POR 21 (4):875-880. doi:10.1007/s12253-015-9902-2

75. Sharlo KA, Paramonova II, Lvova ID, Vilchinskaya NA, Bugrova AE, Shevchenko TF, Kalamkarov GR, Shenkman BS (2020) NO-Dependent Mechanisms of Myosin Heavy Chain Transcription Regulation in Rat Soleus Muscle After 7-Days Hindlimb Unloading. Front Physiol 11:814-814. doi:10.3389/fphys.2020.00814

76. Brozzi F, Lajus S, Diraison F, Rajatileka S, Hayward K, Regazzi R, Molnár E, Váradi A (2012) MyRIP interaction with MyoVa on secretory granules is controlled by the cAMP-PKA pathway. Molecular biology of the cell 23 (22):4444-4455. doi:10.1091/mbc.E12-05-0369

77. Zheng M-Z, Qin H-D, Yu X-J, Zhang R-H, Chen L-Z, Feng Q-S, Zeng Y-X (2007) Haplotype of gene Nedd4 binding protein 2 associated with sporadic nasopharyngeal carcinoma in the Southern Chinese population. J Transl Med 5:36-36. doi:10.1186/1479-5876-5-36

78. Koufaris C, Kirmizis A (2020) N-Terminal Acetyltransferases Are Cancer-Essential Genes Prevalently Upregulated in Tumours. Cancers 12 (9). doi:10.3390/cancers12092631

79. Gromova KV, Muhia M, Rothammer N, Gee CE, Thies E, Schaefer I, Kress S, Kilimann MW, Shevchuk O, Oertner TG, Kneussel M (2018) Neurobeachin and the Kinesin KIF21B Are Critical for Endocytic Recycling of NMDA Receptors and Regulate Social Behavior. Cell reports 23 (9):2705-2717. doi:10.1016/j.celrep.2018.04.112

80. Tian Q, Li Y, Kousar R, Guo H, Peng F, Zheng Y, Yang X, Long Z, Tian R, Xia K, Lin H, Pan Q (2017) A novel NHS mutation causes Nance-Horan Syndrome in a Chinese family. BMC medical genetics 18 (1):2. doi:10.1186/s12881-016-0360-9

81. Troyer JL, Nelson GW, Lautenberger JA, Chinn L, McIntosh C, Johnson RC, Sezgin E, Kessing B, Malasky M, Hendrickson SL, Li G, Pontius J, Tang M, An P, Winkler CA, Limou S, Le Clerc S, Delaneau O, Zagury JF, Schuitemaker H, van Manen D, Bream JH, Gomperts ED, Buchbinder S, Goedert JJ, Kirk GD, O'Brien SJ (2011) Genome-wide association study implicates PARD3B-based AIDS restriction. The Journal of infectious diseases 203 (10):1491-1502. doi:10.1093/infdis/jir046

82. Liu R, Gao J, Yang Y, Qiu R, Zheng Y, Huang W, Zeng Y, Hou Y, Wang S, Leng S, Feng D, Yu W, Sun G, Shi H, Teng X, Wang Y (2018) PHD finger protein 1 (PHF1) is a novel reader for histone H4R3 symmetric dimethylation and coordinates with PRMT5-WDR77/CRL4B complex to promote tumorigenesis. Nucleic acids research 46 (13):6608-6626. doi:10.1093/nar/gky461

83. Ye D, Lou G, Zhang T, Dong F, Liu Y (2018) MiR-17 family-mediated regulation of Pknox1 influences hepatic steatosis and insulin signaling. Journal of cellular and molecular medicine 22 (12):6167-6175. doi:10.1111/jcmm.13902

84. Kouga T, Koizume S, Aoki S, Jimbo E, Yamagata T, Inoue K, Osaka H (2019) Drug screening for Pelizaeus-Merzbacher disease by quantifying the total levels and membrane localization of PLP1. Molecular genetics and metabolism reports 20:100474. doi:10.1016/j.ymgmr.2019.100474

85. Zhan X, Yan C, Zhang X, Lei J, Shi Y (2018) Structure of a human catalytic step I spliceosome. Science (New York, NY) 359 (6375):537-545. doi:10.1126/science.aar6401

86. Li Z, Huang X, Liu A, Xu J, Lai J, Guan H, Ma J (2020) Circ_PSD3 promotes the progression of papillary thyroid carcinoma via the miR-637/HEMGN axis. Life sciences:118622. doi:10.1016/j.lfs.2020.118622

87. Gong S, Xu C, Wang L, Liu Y, Owusu D, Bailey BA, Li Y, Wang K (2017) Genetic association analysis of polymorphisms in PSD3 gene with obesity, type 2 diabetes, and HDL cholesterol. Diabetes research and clinical practice 126:105-114. doi:10.1016/j.diabres.2017.02.006

88. Zhou B, Zhu W, Jiang X, Ren C (2019) RASAL2 Plays Inconsistent Roles in Different Cancers. Frontiers in oncology 9:1235. doi:10.3389/fonc.2019.01235

89. Pistoni M, Verrecchia A, Doni M, Guccione E, Amati B (2010) Chromatin association and regulation of rDNA transcription by the Ras-family protein RasL11a. The EMBO journal 29 (7):1215-1224. doi:10.1038/emboj.2010.16

90. Moua P, Checketts M, Xu LG, Shu HB, Reyland ME, Cusick JK (2017) RELT family members activate p38 and induce apoptosis by a mechanism distinct from TNFR1. Biochemical and biophysical research communications 491 (1):25-32. doi:10.1016/j.bbrc.2017.07.022

91. Nevalainen J, Skarp S, Savolainen ER, Ryynänen M, Järvenpää J (2017) Intrauterine growth restriction and placental gene expression in severe preeclampsia, comparing early-onset and late-onset forms. Journal of perinatal medicine 45 (7):869-877. doi:10.1515/jpm-2016-0406

92. Zhao M, Tan Y, Peng Q, Huang C, Guo Y, Liang G, Zhu B, Huang Y, Liu A, Wang Z, Li M, Gao X, Wu R, Wu H, Long H, Lu Q (2018) IL-6/STAT3 pathway induced deficiency of RFX1 contributes to Th17-dependent autoimmune diseases via epigenetic regulation. Nature communications 9 (1):583. doi:10.1038/s41467-018-02890-0

93. De Franco S, Vandenameele J, Brans A, Verlaine O, Bendak K, Damblon C, Matagne A, Segal DJ, Galleni M, Mackay JP, Vandevenne M (2019) Exploring the suitability of RanBP2-type Zinc Fingers for RNA-binding protein design. Scientific reports 9 (1):2484. doi:10.1038/s41598-019-38655-y

94. Zhan W, Liu Y, Gao Y, Gong R, Wang W, Zhang R, Wu Y, Kang T, Wei D (2020) RMI2 plays crucial roles in growth and metastasis of lung cancer. Signal transduction and targeted therapy 5 (1):188. doi:10.1038/s41392-020-00295-4

95. Jetten AM (2009) Retinoid-related orphan receptors (RORs): critical roles in development, immunity, circadian rhythm, and cellular metabolism. Nucl Recept Signal 7:e003-e003. doi:10.1621/nrs.07003

96. Taneera J, Mohammed AK, Dhaiban S, Hamad M, Prasad RB, Sulaiman N, Salehi A (2019) RORB and RORC associate with human islet dysfunction and inhibit insulin secretion in INS-1 cells. Islets 11 (1):10-20. doi:10.1080/19382014.2019.1566684

97. Chen L, Dumelie JG, Li X, Cheng MH, Yang Z, Laver JD, Siddiqui NU, Westwood JT, Morris Q, Lipshitz HD, Smibert CA (2014) Global regulation of mRNA translation and stability in the early Drosophila embryo by the Smaug RNA-binding protein. Genome biology 15 (1):R4. doi:10.1186/gb-2014-15-1-r4

98. Lu M, Wang W, Zhang S, Li Y (2018) SEC24A stimulates oncogenicity of human gastric cancer cells. Int J Clin Exp Pathol 11 (8):4044-4051

99. Yang C, Li J, Sun F, Zhou H, Yang J, Yang C (2020) The functional duality of SGK1 in the regulation of hyperglycemia. Endocrine connections 9 (7):R187-r194. doi:10.1530/ec-20-0225

100. Zhang Y, Feng Y, Xin Y, Liu X (2018) SGIP1 dimerizes via intermolecular disulfide bond in μHD domain during cellular endocytosis. Biochemical and biophysical research communications 505 (1):99-105. doi:10.1016/j.bbrc.2018.09.075

101. Katagiri S, Hayashi T, Yoshitake K, Murai N, Matsui Z, Kubo H, Satoh H, Matsufuji S, Takamura T, Yokoo T, Omori Y, Furukawa T, Iwata T, Nakano T (2018) Compound heterozygous splice site variants in the SCLT1 gene highlight an additional candidate locus for Senior-Løken syndrome. Scientific reports 8 (1):16733. doi:10.1038/s41598-018-35152-6

102. Osterberg L, Levan K, Partheen K, Delle U, Olsson B, Sundfeldt K, Horvath G (2009) Potential predictive markers of chemotherapy resistance in stage III ovarian serous carcinomas. BMC cancer 9:368. doi:10.1186/1471-2407-9-368

103. Räschle M, Smeenk G, Hansen RK, Temu T, Oka Y, Hein MY, Nagaraj N, Long DT, Walter JC, Hofmann K, Storchova Z, Cox J, Bekker-Jensen S, Mailand N, Mann M (2015) DNA repair. Proteomics reveals dynamic assembly of repair complexes during bypass of DNA cross-links. Science (New York, NY) 348 (6234):1253671. doi:10.1126/science.1253671

104. Calpena E, Cuellar A, Bala K, Swagemakers SMA, Koelling N, McGowan SJ, Phipps JM, Balasubramanian M, Cunningham ML, Douzgou S, Lattanzi W, Morton JEV, Shears D, Weber A, Wilson LC, Lord H, Lester T, Johnson D, Wall SA, Twigg SRF, Mathijssen IMJ, Boardman-Pretty F, Boyadjiev SA, Wilkie AOM (2020) SMAD6 variants in craniosynostosis: genotype and phenotype evaluation. Genetics in medicine : official journal of the American College of Medical Genetics 22 (9):1498-1506. doi:10.1038/s41436-020-0817-2

105. Lan Y, Sullivan PM, Hu F (2019) SMCR8 negatively regulates AKT and MTORC1 signaling to modulate lysosome biogenesis and tissue homeostasis. Autophagy 15 (5):871-885. doi:10.1080/15548627.2019.1569914

106. Katoh Y, Katoh M (2005) Hedgehog signaling pathway and gastric cancer. Cancer biology & therapy 4 (10):1050-1054. doi:10.4161/cbt.4.10.2184

107. Ji C, Tang M, Zeidler C, Höhfeld J, Johnson GV (2019) BAG3 and SYNPO (synaptopodin) facilitate phospho-MAPT/Tau degradation via autophagy in neuronal processes. Autophagy 15 (7):1199-1213. doi:10.1080/15548627.2019.1580096

108. Manshouri R, Coyaud E, Kundu ST, Peng DH, Stratton SA, Alton K, Bajaj R, Fradette JJ, Minelli R, Peoples MD, Carugo A, Chen F, Bristow C, Kovacs JJ, Barton MC, Heffernan T, Creighton CJ, Raught B, Gibbons DL (2019) ZEB1/NuRD complex suppresses TBC1D2b to stimulate E-cadherin internalization and promote metastasis in lung cancer. Nature communications 10 (1):5125. doi:10.1038/s41467-019-12832-z

109. Toyofuku T, Morimoto K, Sasawatari S, Kumanogoh A (2015) Leucine-Rich Repeat Kinase 1 Regulates Autophagy through Turning On TBC1D2-Dependent Rab7 Inactivation. Molecular and cellular biology 35 (17):3044-3058. doi:10.1128/mcb.00085-15

110. Hirano S, Uemura T, Annoh H, Fujita N, Waguri S, Itoh T, Fukuda M (2016) Differing susceptibility to autophagic degradation of two LC3-binding proteins: SQSTM1/p62 and TBC1D25/OATL1. Autophagy 12 (2):312-326. doi:10.1080/15548627.2015.1124223

111. Guo S, Liu Y, Gao L, Xiao F, Shen J, Xing S, Yang F, Zhang W, Shi Q, Li Y, Zhao L (2020) TBC1D25 Regulates Cardiac Remodeling Through TAK1 Signaling Pathway. International journal of biological sciences 16 (8):1335-1348. doi:10.7150/ijbs.41130

112. Çalışkan M, Chong JX, Uricchio L, Anderson R, Chen P, Sougnez C, Garimella K, Gabriel SB, dePristo MA, Shakir K, Matern D, Das S, Waggoner D, Nicolae DL, Ober C (2011) Exome sequencing reveals a novel mutation for autosomal recessive non-syndromic mental retardation in the TECR gene on chromosome 19p13. Human molecular genetics 20 (7):1285-1289. doi:10.1093/hmg/ddq569

113. Dion SP, Béliveau F, Morency LP, Désilets A, Najmanovich R, Leduc R (2018) Functional diversity of TMPRSS6 isoforms and variants expressed in hepatocellular carcinoma cell lines. Scientific reports 8 (1):12562. doi:10.1038/s41598-018-30618-z

114. Rome KS, Stein SJ, Kurachi M, Petrovic J, Schwartz GW, Mack EA, Uljon S, Wu WW, DeHart AG, McClory SE, Xu L, Gimotty PA, Blacklow SC, Faryabi RB, Wherry EJ, Jordan MS, Pear WS (2020) Trib1 regulates T cell differentiation during chronic infection by restraining the effector program. The Journal of experimental medicine 217 (5). doi:10.1084/jem.20190888

115. Nadolni W, Zierler S (2018) The Channel-Kinase TRPM7 as Novel Regulator of Immune System Homeostasis. Cells 7 (8). doi:10.3390/cells7080109

116. Yee NS, Kazi AA, Yee RK (2014) Cellular and Developmental Biology of TRPM7 Channel-Kinase: Implicated Roles in Cancer. Cells 3 (3):751-777. doi:10.3390/cells3030751

117. Lo CH, Lin IH, Yang TT, Huang YC, Tanos BE, Chou PC, Chang CW, Tsay YG, Liao JC, Wang WJ (2019) Phosphorylation of CEP83 by TTBK2 is necessary for cilia initiation. The Journal of cell biology 218 (10):3489-3505. doi:10.1083/jcb.201811142

118. Zheng J, Liu X, Xue Y, Gong W, Ma J, Xi Z, Que Z, Liu Y (2017) TTBK2 circular RNA promotes glioma malignancy by regulating miR-217/HNF1β/Derlin-1 pathway. Journal of hematology & oncology 10 (1):52. doi:10.1186/s13045-017-0422-2

119. Nasoohi S, Ismael S, Ishrat T (2018) Thioredoxin-Interacting Protein (TXNIP) in Cerebrovascular and Neurodegenerative Diseases: Regulation and Implication. Molecular neurobiology 55 (10):7900-7920. doi:10.1007/s12035-018-0917-z

120. Kumar N, Leonzino M, Hancock-Cerutti W, Horenkamp FA, Li P, Lees JA, Wheeler H, Reinisch KM, De Camilli P (2018) VPS13A and VPS13C are lipid transport proteins differentially localized at ER contact sites. The Journal of cell biology 217 (10):3625-3639. doi:10.1083/jcb.201807019

121. Kanca O, Andrews JC, Lee PT, Patel C, Braddock SR, Slavotinek AM, Cohen JS, Gubbels CS, Aldinger KA, Williams J, Indaram M, Fatemi A, Yu TW, Agrawal PB, Vezina G, Simons C, Crawford J, Lau CC, Chung WK, Markello TC, Dobyns WB, Adams DR, Gahl WA, Wangler MF, Yamamoto S, Bellen HJ, Malicdan MCV (2019) De Novo Variants in WDR37 Are Associated with Epilepsy, Colobomas, Dysmorphism, Developmental Delay, Intellectual Disability, and Cerebellar Hypoplasia. American journal of human genetics 105 (2):413-424. doi:10.1016/j.ajhg.2019.06.014

122. Kim Y, Kim W, Song Y, Kim JR, Cho K, Moon H, Ro SW, Seo E, Ryu YM, Myung SJ, Jho EH (2017) Deubiquitinase YOD1 potentiates YAP/TAZ activities through enhancing ITCH stability. Proceedings of the National Academy of Sciences of the United States of America 114 (18):4691-4696. doi:10.1073/pnas.1620306114

123. Schimmack G, Schorpp K, Kutzner K, Gehring T, Brenke JK, Hadian K, Krappmann D (2017) YOD1/TRAF6 association balances p62-dependent IL-1 signaling to NF-κB. eLife 6. doi:10.7554/eLife.22416

124. Vaquerizas JM, Kummerfeld SK, Teichmann SA, Luscombe NM (2009) A census of human transcription factors: function, expression and evolution. Nature reviews Genetics 10 (4):252-263. doi:10.1038/nrg2538

125. Vernet N, Mahadevaiah SK, de Rooij DG, Burgoyne PS, Ellis PJI (2016) Zfy genes are required for efficient meiotic sex chromosome inactivation (MSCI) in spermatocytes. Human Molecular Genetics 25 (24):5300-5310. doi:10.1093/hmg/ddw344

126. Razzaghi H, Santorico SA, Kamboh MI (2012) Population-Based Resequencing of LIPG and ZNF202 Genes in Subjects with Extreme HDL Levels. Frontiers in genetics 3:89. doi:10.3389/fgene.2012.00089

127. Gupta AR, Westphal A, Yang DYJ, Sullivan CAW, Eilbott J, Zaidi S, Voos A, Vander Wyk BC, Ventola P, Waqar Z, Fernandez TV, Ercan-Sencicek AG, Walker MF, Choi M, Schneider A, Hedderly T, Baird G, Friedman H, Cordeaux C, Ristow A, Shic F, Volkmar FR, Pelphrey KA (2017) Neurogenetic analysis of childhood disintegrative disorder. Molecular autism 8:19. doi:10.1186/s13229-017-0133-0

128. Gaudet P, Livstone MS, Lewis SE, Thomas PD (2011) Phylogenetic-based propagation of functional annotations within the Gene Ontology consortium. Briefings in bioinformatics 12 (5):449-462. doi:10.1093/bib/bbr042

129. Liu X, Zhao X, Gou C (2019) Identification of novel methylated DNA marker ZNF569 for head and neck squamous cell carcinoma. Journal of Cancer 10 (10):2250-2260. doi:10.7150/jca.31156

130. Li HD, Chen X, Xu JJ, Du XS, Yang Y, Li JJ, Yang XJ, Huang HM, Li XF, Wu MF, Zhang C, Zhang C, Li Z, Wang H, Meng XM, Huang C, Li J (2020) DNMT3b-mediated methylation of ZSWIM3 enhances inflammation in alcohol-induced liver injury via regulating TRAF2-mediated NF-κB pathway. Clinical science (London, England : 1979) 134 (14):1935-1956. doi:10.1042/cs20200031

131. Sundaramoorthy S, Ryu MS, Lim IK (2013) B-cell translocation gene 2 mediates crosstalk between PI3K/Akt1 and NFκB pathways which enhances transcription of MnSOD by accelerating IκBα degradation in normal and cancer cells. Cell communication and signaling : CCS 11:69. doi:10.1186/1478-811x-11-69

132. Agresta L, Hoebe KHN, Janssen EM (2018) The Emerging Role of CD244 Signaling in Immune Cells of the Tumor Microenvironment. Frontiers in immunology 9:2809. doi:10.3389/fimmu.2018.02809

133. Zhang F, Liu X, Chen C, Zhu J, Yu Z, Xie J, Xie L, Bai H, Zhang Y, Fang X, Gu H, Wang C, Weng W, Zhang CC, Chen GQ, Liang A, Zheng J (2017) CD244 maintains the proliferation ability of leukemia initiating cells through SHP-2/p27(kip1) signaling. Haematologica 102 (4):707-718. doi:10.3324/haematol.2016.151555

134. Jiang W, Yu Y, Liu J, Zhao Q, Wang J, Zhang J, Dang X (2019) Downregulation of Cdc6 inhibits tumorigenesis of osteosarcoma in vivo and in vitro. Biomedicine & pharmacotherapy = Biomedecine & pharmacotherapie 115:108949. doi:10.1016/j.biopha.2019.108949

135. Tillgren V, Ho JC, Önnerfjord P, Kalamajski S (2015) The novel small leucine-rich protein chondroadherin-like (CHADL) is expressed in cartilage and modulates chondrocyte differentiation. The Journal of biological chemistry 290 (2):918-925. doi:10.1074/jbc.M114.593541

136. Trivedi G, Inoue D, Chen C, Bitner L, Chung YR, Taylor J, Gönen M, Wess J, Abdel-Wahab O, Zhang L (2019) Muscarinic acetylcholine receptor regulates self-renewal of early erythroid progenitors. Science translational medicine 11 (511). doi:10.1126/scitranslmed.aaw3781

137. Owusu-Ansah KG, Song G, Chen R, Edoo MIA, Li J, Chen B, Wu J, Zhou L, Xie H, Jiang D, Zheng S (2019) COL6A1 promotes metastasis and predicts poor prognosis in patients with pancreatic cancer. International journal of oncology 55 (2):391-404. doi:10.3892/ijo.2019.4825

138. Deng L, Ren R, Liu Z, Song M, Li J, Wu Z, Ren X, Fu L, Li W, Zhang W, Guillen P, Izpisua Belmonte JC, Chan P, Qu J, Liu GH (2019) Stabilizing heterochromatin by DGCR8 alleviates senescence and osteoarthritis. Nature communications 10 (1):3329. doi:10.1038/s41467-019-10831-8

139. Zhang F, Ruan X, Ma J, Liu X, Zheng J, Liu Y, Liu L, Shen S, Shao L, Wang D, Yang C, Cai H, Li Z, Feng Z, Xue Y (2020) DGCR8/ZFAT-AS1 Promotes CDX2 Transcription in a PRC2 Complex-Dependent Manner to Facilitate the Malignant Biological Behavior of Glioma Cells. Molecular therapy : the journal of the American Society of Gene Therapy 28 (2):613-630. doi:10.1016/j.ymthe.2019.11.015

140. Wang H, Zhong C, Yang R, Yin Y, Tan R, Gao L, Gao C, Cui Y, Pu D, Wu J (2020) Hfm1 participates in Golgi-associated spindle assembly and division in mouse oocyte meiosis. Cell death & disease 11 (6):490. doi:10.1038/s41419-020-2697-4

141. Lai NS, Yu HC, Huang KY, Tung CH, Huang HB, Lu MC (2018) Decreased T cell expression of H/ACA box small nucleolar RNA 12 promotes lupus pathogenesis in patients with systemic lupus erythematosus. Lupus 27 (9):1499-1508. doi:10.1177/0961203318778362

142. Li R, Yin YH, Jin J, Liu X, Zhang MY, Yang YE, Qu YQ (2020) Integrative analysis of DNA methylation-driven genes for the prognosis of lung squamous cell carcinoma using MethylMix. International journal of medical sciences 17 (6):773-786. doi:10.7150/ijms.43272

143. Dudem S, Large RJ, Kulkarni S, McClafferty H, Tikhonova IG, Sergeant GP, Thornbury KD, Shipston MJ, Perrino BA, Hollywood MA (2020) LINGO1 is a regulatory subunit of large conductance, Ca(2+)-activated potassium channels. Proceedings of the National Academy of Sciences of the United States of America 117 (4):2194-2200. doi:10.1073/pnas.1916715117

144. Meza-Zepeda LA, Forus A, Lygren B, Dahlberg AB, Godager LH, South AP, Marenholz I, Lioumi M, Flørenes VA, Maelandsmo GM, Serra M, Mischke D, Nizetic D, Ragoussis J, Tarkkanen M, Nesland JM, Knuutila S, Myklebost O (2002) Positional cloning identifies a novel cyclophilin as a candidate amplified oncogene in 1q21. Oncogene 21 (14):2261-2269. doi:10.1038/sj.onc.1205339

145. Myhre JL, Hills JA, Jean F, Pilgrim DB (2014) Unc45b is essential for early myofibrillogenesis and costamere formation in zebrafish. Developmental biology 390 (1):26-40. doi:10.1016/j.ydbio.2014.02.022
